# Exploring novel of 1,2,4-triazolo[4,3-*a*]quinoxaline sulfonamide regioisomers as anti-diabetic and anti-Alzheimer agents with* in-silico* molecular docking simulation

**DOI:** 10.1038/s41598-025-03139-9

**Published:** 2025-06-03

**Authors:** Moustafa S. Abusaif, Ahmed M. Sh El-Sharief, Yehia A. Mohamed, Yousry A. Ammar, Mostafa A. Ismail, Wael M. Aboulthana, Mohamed S. A. El-Gaby, Ahmed Ragab

**Affiliations:** 1 Chemistry Department, Faculty of Science (boys), Al-Azhar University, Nasr City, 11884 Cairo Egypt; 2 Chemistry Department, Faculty of Science, Al-Azhar University, Assiut, 71524 Egypt; 3 Biochemistry Department, Biotechnology Research Institute, National Research Centre, 33 El Buhouth St., Dokki 12622, Cairo, Egypt; 4 Chemistry Department, Faculty of Science, Galala University, Galala City, Suez, 43511 Egypt

**Keywords:** 1,2,4-triazolo[4,3-*a*]quinoxaline, Regioselective synthesis, Α-amylase and α-glucosidase inhibitors, Acetylcholinesterase inhibitors, Molecular docking simulation, Chemical biology, Drug discovery

## Abstract

In this study, a novel series of 1,2,4-triazolo[4,3-*a*]quinoxalines containing a sulfonamide moiety was designed and synthesized through regioselective synthesis from 2 and/ 3-hydrazino-6-(pyrrolidin-1-ylsulfonyl)quinoxaline derivatives **5** and **7**. The structures of two isomers were confirmed and characterized by IR,^1^H NMR,^13^C NMR, and elemental analysis data. The synthesized 1,2,4-triazolo[4,3-*a*]quinoxaline derivatives **8–13** were evaluated for their antidiabetic activities by targeting α-amylase and α-glucosidase, as well as for their anti-Alzheimer activity by targeting acetylcholinesterase (AChE) at a concentration of 100 µM. Structure-activity relationship (SAR) analysis was conducted for all analogs, emphasizing the nature of the substituent groups at position one of the triazole nucleus and the positioning of the sulfonamide moiety. For α-amylase and α-glucosidase activity, the designed compounds exhibited moderate to good activity, with inhibitory percentage values ranging from 21.85 ± 0.01% to 64.70 ± 0.02% and from 23.93 ± 0.01% to 75.36 ± 0.01%, respectively. The *N*-allyl-[1,2,4]triazolo[4,3-*a*]quinoxalin-1-amine derivative **10a** demonstrated the most significant inhibitory activity, with percentages of 64.70 ± 0.02% and 75.36 ± 0.01% against α-amylase and α-glucosidase, respectively, in comparison to acarbose (IP = 67.33 ± 0.01% and 57.79 ± 0.01%). Furthermore, the 1,2,4-triazolo[4,3-*a*]quinoxaline derivatives **8–13** exhibited low to moderate inhibitory percentages against the acetylcholinesterase enzyme, except for the 1-methyl-[1,2,4]triazolo[4,3-*a*]quinoxaline derivative **11b** which demonstrated the highest inhibitory percentage of 44.78 ± 0.01%, compared to donepezil (IP = 67.27 ± 0.60%). Moreover, the promising derivative **10a** demonstrated exceptional inhibitory activity, exhibiting IC_50_ values of 3.46 ± 0.06 µM and 6.89 ± 0.09 µM against α-glucosidase and α-amylase, respectively, when compared to acarbose, which has IC_50_ values of 4.27 ± 0.06 µM and 5.90 ± 0.09 µM. Finally, molecular docking simulations were performed for compound **10a** within α-amylase (PDB: 2QV4) and α-glucosidase (PDB: 3W37), while compound **11b** was analyzed within acetylcholinesterase (AChE) (PDB: 4EY7) to assess binding affinity and to explore the binding interactions with the active sites of the enzymes.

**Supplementary Information:**

The online version contains supplementary material available at 10.1038/s41598-025-03139-9.

## Introduction

Diabetes mellitus (hyperglycemia) is a long-term metabolic problem that causes blood sugar levels to be too high because of insulin deficiency (type 1) or insulin resistance (type 2). It is a significant threat to people all over the world^1^. A higher probability of cancer, kidney failure, blindness, and amputation^2^, as well as a heightened susceptibility to bone fractures^3^, are all associated with diabetes mellitus^4^. Diabetes is associated with a complex cascade of numerous metabolic and signalling pathways in its pathophysiology^5^. When blood sugar levels are high, auto-oxidative glycosylation occurs. It sets off protein kinase C and improves the polyol cascade. These activities generate ROS (reactive oxygen species) and RNS (reactive nitrogen species), causing oxidative stress^6,7^. It is imperative to develop antidiabetic medications that effectively address the significant risks associated with diabetes mellitus while minimizing adverse effects^8^. Two critical anti-diabetic pharmaceuticals approved by the Food and Drug Administration (FDA) that regulate hyperglycemia are acarbose and miglitol, which function by inhibiting the carbohydrate-hydrolyzing enzymes α-amylase and α-glucosidase^9,10^. Despite their demonstrated efficacy, these medications can elicit substantial adverse side effects, particularly gastrointestinal issues attributed to acarbose and miglitol^11,12^. However, the long-term use of these hypoglycemic agents has been associated with adverse and often unavoidable side effects, such as congestive heart failure, inflammation, cellular apoptosis, pancreatitis, and gastrointestinal discomfort.

On the other hand, Alzheimer’s disease (AD) is detected by the presence of dementia, which usually starts with mild difficulties in recognizing things and remembering information^13^. The cholinergic system in AD is particularly prone to synapse loss, notably in cortical regions that are linked to memory and executive function^14^. Recent research has indicated that the primary factor contributing to the decline of cognitive abilities in individuals with Alzheimer’s disease (AD) is a gradual decrease in cholinergic neurotransmission in several parts of the human brain, including the cortex^15^. A acetylcholinesterase (AChE) and Butyrylcholinesterase (BChE) are hydrolytic enzymes that cleave acetylcholine (ACh) into choline and acetate in the synaptic cleft to stop its effects. Both enzymes are valid targets for improving the cholinergic deficit that is believed to be the cause of the cognitive, behavioral, and global functional deterioration seen in Alzheimer’s disease^16^.

**Fig. 1:**
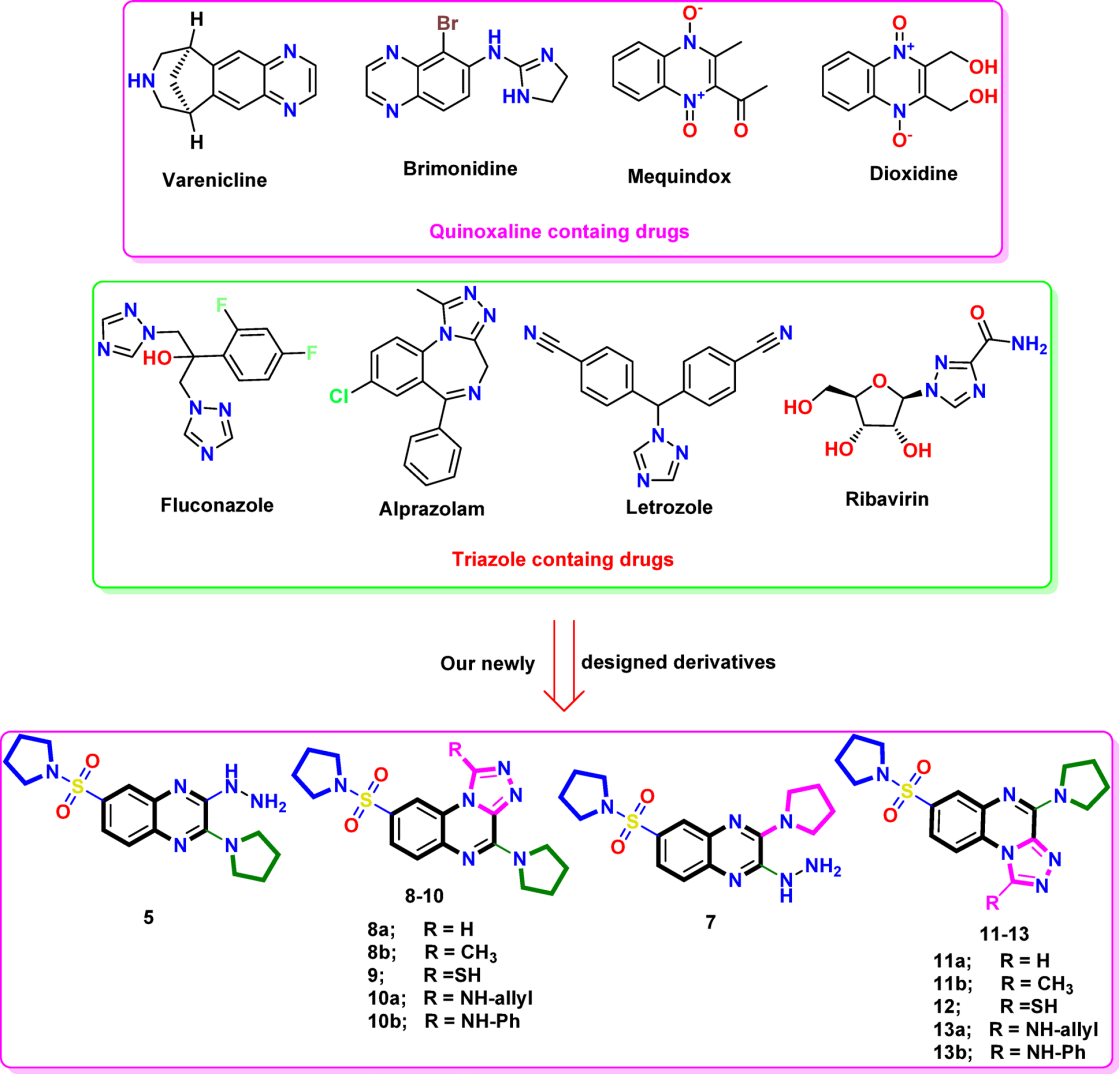
The rational study focused on quinoxaline and triazole containing drugs, including our newly designed triazole-quinoxaline derivatives.

Therefore, therapeutic approaches for Alzheimer’s disease (AD) were established in the form of inhibitors of AChE (acetylcholinesterase) and BChE (butyrylcholinesterase). Scientists have given Alzheimer’s disease (AD) the nickname “Type-3-Diabetes” due to its molecular and cellular similarities with insulin resistance, memory problems, and cognitive decline in older individuals^17^. Therefore, among the many physiologically active quinoxaline compounds are antibiotics with activity against a variety of transplantable malignancies, including levomycin, actinoleutin, and echinomycin^18^. Figure 1 depicts three clinical examples of medications that include quinoxaline. These drugs include Varenicline, which aids in smoking cessation, Brimonidine, which has antiglaucoma qualities^19^, and Quinacillin, which has antibacterial properties^20^ (Fig. 1). According to the literature, the majority of quinoxaline-related compounds have been found to possess antidiabetic, anticancer, anti-inflammatory, antimalarial, antitubercular, anti-HIV, insecticidal, acetylcholine-esterase, and butyrylcholinesterase inhibitory properties^21-27^. Although these drugs are widely used, they have been associated with a range of adverse effects, such as liver damage, renal failure, diarrhea, bloating, flatulence, pain, and stomach discomfort. Medicinal chemists are currently focused on designing, synthesizing, and developing new α-glucosidase inhibitors with fewer side effects^28,29^.

The triazole moiety is essential in pharmaceutical chemistry. Selective triazoles play a crucial role in the drug development process for heterocyclic bioactive compounds exhibiting a broad spectrum of activities^30^. When this ring is associated with other heterocyclic rings, it may lead to enhanced efficacy against α-glucosidase. Over the past two decades, researchers have synthesized numerous indole derivatives to discover novel molecules^31^. Also, the class of organic compounds known as substituted 1,2,4-triazoles is significant due to their broad pharmacological activities, which include antibacterial^32,33^, antifungal^34^, antimycobacterial^35^, anti-inflammatory^32,36^, and anticancer^37^ properties. Fluconazole^38^, Alprazolam, Letrozole, and Ribavirin are widely used medications in the class of 1,2,4-triazoles^39^. These drugs are recognized for their fluoro- and trifluoromethyl-substituted properties. On the other hand, sulfonamides are well recognized for their significant therapeutic and pharmacological importance. The sulfonamide moiety (–SO_2_N) is a pharmacophore that exhibits a diverse array of actions, such as antibacterial, antimalarial, anti-HIV, high ceiling diuretic, antithyroid, anticancer effects^40-42^, and insulin-releasing antidiabetic activity, which is particularly significant^43^. Pyrrolidine is classified as a nitrogenous heterocyclic compound that serves as a privileged scaffold in medicinal chemistry due to its biological activity, structural versatility, and pharmacokinetic properties^44^. Furthermore, its presence in numerous bioactive synthetic drugs, alkaloids, and natural products, as well as its role as a pharmaceutical intermediate, underscores its significance in drug development and discovery^10^. This importance may be attributed to its lipophilic properties, which contribute to membrane permeability and enhanced metabolic stability. The pyrrolidine scaffold has demonstrated a wide range of biological activities, including anticancer, antimicrobial, pesticide, antiviral, anti-diabetic, and anti-inflammatory effects^45-47^.

Based on the aforementioned critical features and building upon our prior research in the design and synthesis of novel bioactive agents from heterocyclic compounds^48-55^, this study was designed to synthesize new regioisomer sulfonamide-quinoxaline derivatives incorporating a novel hybrid core, specifically 1,2,4-triazole. The designed derivatives were evaluated for their antidiabetic properties against α-amylase and α-glucosidase, as well as for their potential as anti-Alzheimer agents against acetylcholinesterase to be used as dual-target drug therapies. Additionally, the structure-activity relationship (SAR) study provided detailed insights into the effects of different substituents at the N1 position of triazole and the placement of the pyrrolidin-1-ylsulfonyl moiety. Finally, molecular docking simulations were conducted for the most active derivatives within the active sites of three enzymes: α-amylase (PDB: 2QV4), α-glucosidase (PDB: 3W37), and acetylcholinesterase (ACHE) (PDB: 4EY7). These simulations aimed to elucidate the binding modes and types of interactions, as well as to assess the binding affinity of each derivative in comparison to positive control drugs.

## Results and discussion

### Chemistry

**Scheme 1:**
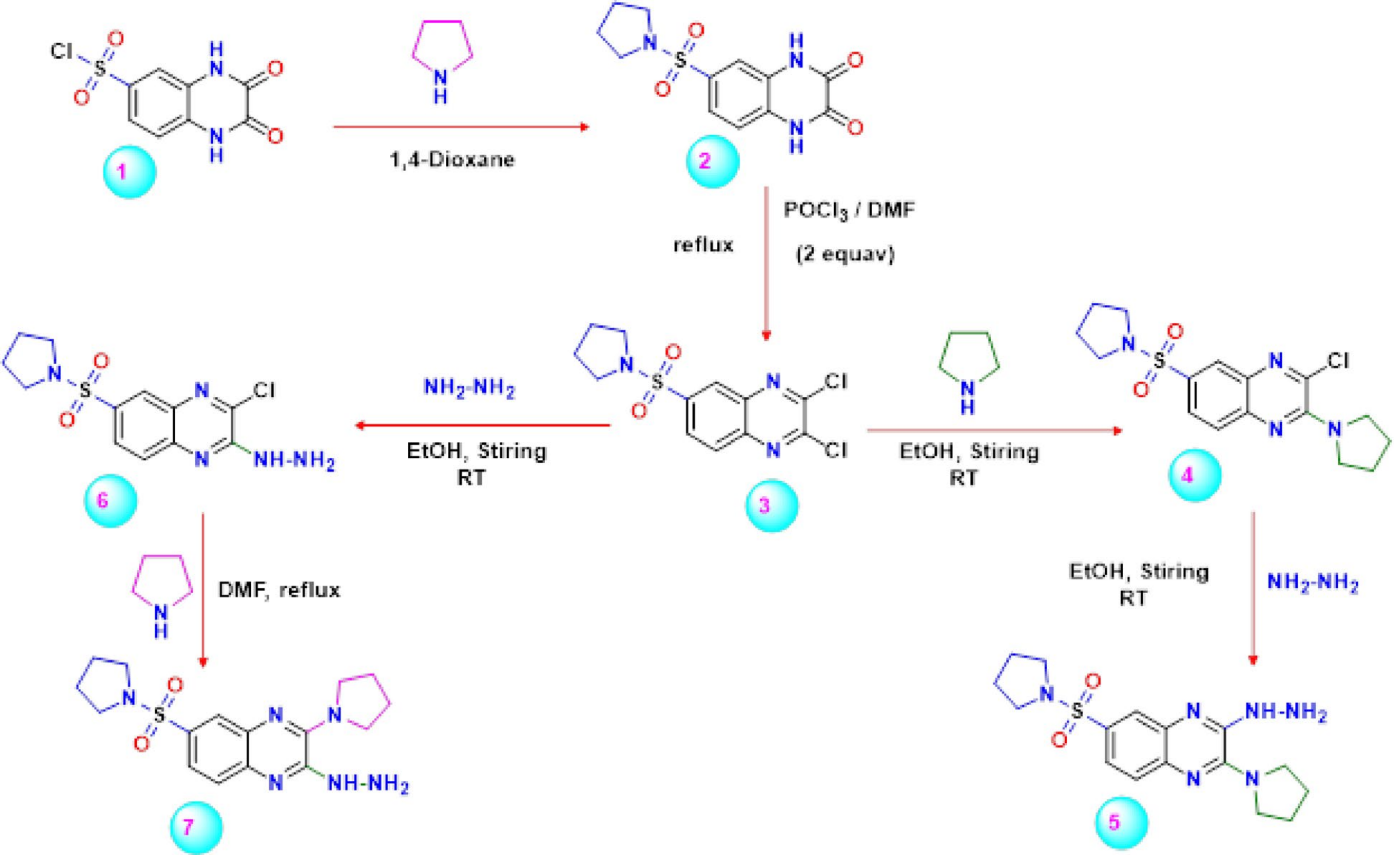
Synthetic strategy for synthesis of two isomer 2,3-hydrazino-6-(pyrrolidin-1-ylsulfonyl)quinoxaline derivatives 5 and 7.

For over a century, nucleophilic substitution processes in aromatic carbon have been the focus of several research articles, which are of tremendous interest due to their potential for both academic and industrial synthetic applications^56,57^. The two reactive chlorine atoms in 2,3-dichloroquionoxaline **3** are vulnerable to nucleophilic displacement reactions by a wide variety of nucleophiles that react sequentially. Also, the functionalization of 2,3-dichloroquinoxaline with various nitrogen nucleophiles generated a large number of novel 2,3-disubstituted quinoxalines and condensed quinoxalines. Therefore, the authors examined the feasibility of substituting different nucleophiles for the active chlorine atom in 2,3-dichloroquinoxaline derivative **3** in the presence of an electron-withdrawing substituent at position-6 of the quinoxaline moiety. As mentioned in our previous work^58-61^, the key intermediate 2,3-dichloro-6-(pyrrolidin-1-ylsulfonyl)quinoxaline (**3**) was obtained through the reaction of sulfonyl chloride derivative **1** with pyrrolidine in refluxing 1,4-dioxane to produce the corresponding 6-(pyrrolidin-1-ylsulfonyl)-1,4-dihydroquinoxaline-2,3-dione **2** in good yield, then reacting with two equivalents of phosphorus oxychloride in dimethylformamide (Scheme 1)^62^. Initially, the reactivity of the 2,3-dichloroquinoxaline derivative **3** towards some nucleophiles like pyrrolidine and hydrazine in positions **C**_**2**_ & **C**_**3**_ were examined. Thus, when the 2,3-dichloroquinoxaline derivative **3** was subjected to react with pyrrolidine at room temperature in ethanol, it formed a yellow product with interesting structure 4, where the pyrrolidine nucleus entered in position **C**_**2**_ of quinoxaline. On the other hand, treating the starting material **3** with hydrazine at room temperature in ethanol formed orange crystals with interesting isomer structure 6 at **C**_**2**_ of 6-sulfonyl quinoxaline (Scheme 1). The infrared spectrum of compound **4** showed an absorption band at 3040 cm^− 1^ for the CH-arom group, at 2965, 2835 cm^− 1^ for the CH-aliph group, 1639 cm^− 1^ for the C = N group, and 1324, 1141 cm^− 1^ for the SO_2_ group. The^1^H NMR spectrum of the same compound demonstrated two multiples at δ 1.93–2.03 and 3.73–3.75 ppm ascribed to the pyrrolidine ring. The signals at *δ* 7.73 (d, 1 H, *J* = 8.0 Hz), 7.93 (d, 1 H, J = 8.0 Hz), and 8.07 (s, 1 H) attributed to C_7_, C_8_, and C_5_ of the quinoxaline ring, respectively. Furthermore, their^13^C NMR spectrum revealed the presence of two signals at *δ* 25.28 (4CH_2_) and 50.17 (4CH_2_), indicating the existence of the two pyrrolidine moieties as well as signals at *δ* 125.42, 127.90, 129.10, 130.29, 131.77, 135.93, and 144.59 ppm, corresponding to different kinds of carbon atom. The most downfield signal appeared at *δ* 160.57 ppm, delivered to the C = N group. On the other hand, the^1^ H NMR spectrum of the other isomer **6** revealed the expected signals, two singlet signals exchangeable with D_2_O indicating the presence of NH and NH_2_ groups, as well as the presence of the following signals in the^13^C NMR spectrum at *δ* 25.57 (2CH_2_, pyrrolidine), 49.12 (2 N-CH_2_, pyrrolidine), 125.21, 126.36, 127.77, 129.14, 133.32, 137.99, 143.79, and 153.77 ppm (N = C).

**Scheme 2:**
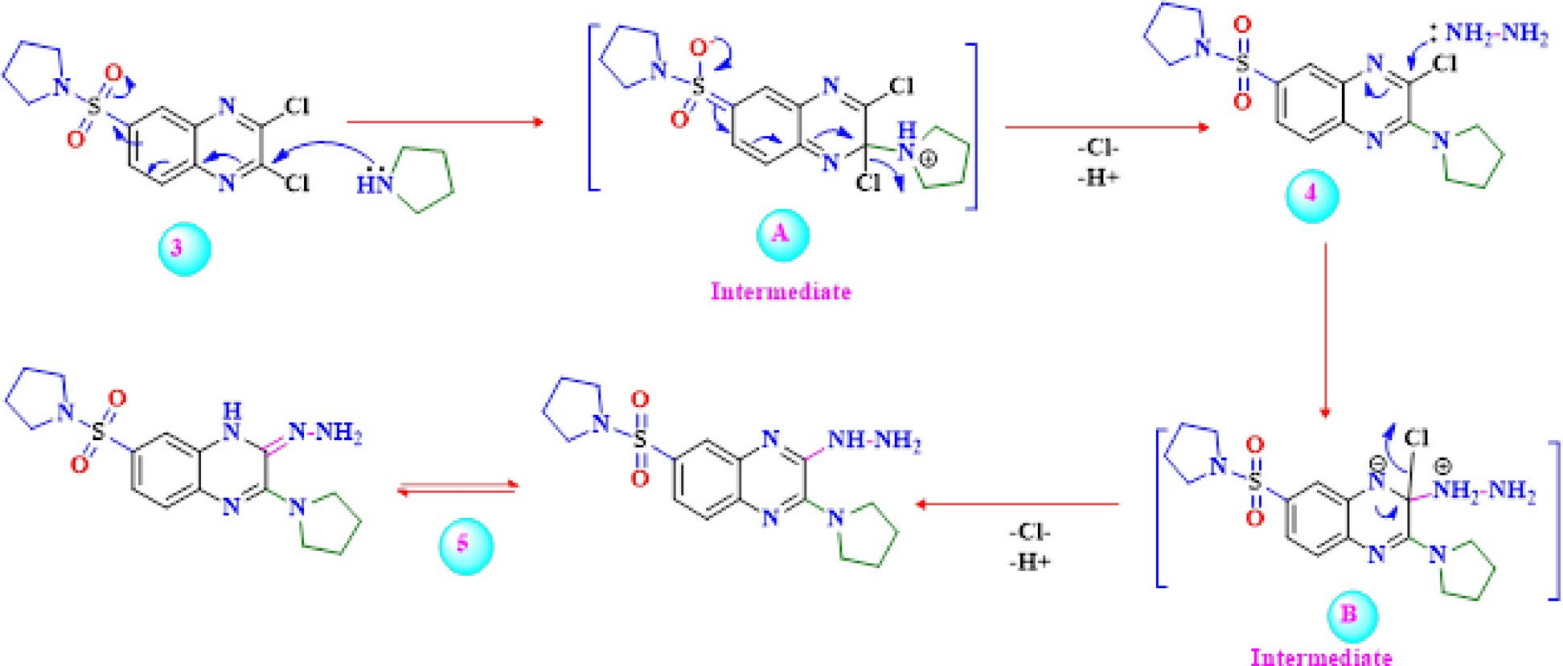
Mechanismatic pathway for formation of 3-hydrazino-quinoxaline derivative** 5 **

Based on all of this data, the chlorine atom in position**-2** in compound **3** is most affected by the electron-withdrawing effect of the sulfonyl group. It has the potential to undergo facile nucleophilic displacement. A proposed mechanism for forming 3-chloro-2-(pyrrolidin-1-yl)-6-(pyrrolidin-1-ylsulfonyl)quinoxaline **4** as an example, is depicted in Scheme 2. The formation of compound **4** is assumed to occur through the nucleophilic addition of pyrrolidine to the carbon atom of the cyclic imine group in compound **3** at position-2 to generate the non-isolable intermediate **A**, followed by the elimination of a hydrogen chloride molecule. Continuously, the replacement of the second chlorine at position-3 of compound **4** with hydrazine was carried out in ethanol at ambient temperature to provide 3-hydrazino-2-(pyrrolidin-1-yl)-6-(pyrrolidin-1-ylsulfonyl)quinoxaline **5** in moderate yield (**67%**) (Scheme 2). On the other hand, the other isomer of 2-hydrazino-3-(pyrrolidin-1-yl)-6-(pyrrolidin-1-ylsulfonyl)quinoxaline **7** was obtained by substituting the second chlorine atom at position-3 in the 3-chloro-quinoxaline derivative **6** with pyrrolidine in ethanol at ambient temperature (Scheme 2). The IR spectrum exhibited bands related to NH and NH_2_ signals at 3499, 3315, and 3185 cm^− 1^, providing evidence for the formation of compound **5**. Also, the^1^H NMR spectrum of **5** showed a multiplet at *δ* 1.60–1.63 ppm corresponding to the four CH_2_ groups, a triplet at *δ* 3.07 ppm equivalent to the four CH_2_ groups, a broad at *δ* 7.00 ppm D_2_O exchangeable for the NH and NH_2_ groups, as well as the signals at *δ* 7.35 (d, 2 H, *J* = 8.4 Hz, H**7**.quinox, H**8**.quinox) and 7.53 (s, 1 H, H**5**.quinox) were observed. Moreover, the 1 H NMR spectrum of product **7** contained two singlets at *δ* 1.64 and 3.17 ppm, which are assignable to two pyrrolidine moieties, as well as two broad bands at *δ* 7.31 and 7.43 ppm, assignable to NH_2_ and NH protons, respectively. In addition, the^13^C NMR spectrum of the isomer **5** showed the existence of seven signals at *δ* 125.13, 128.02, 129.62, 131.35, 133.55, 138.05, and 147.21 ppm corresponding to aromatic carbons, two signals at *δ* 26.56 and 54.76 for pyrrolidine carbons, and a signal at *δ* 156.60 related to the C = N group. The addition-elimination mechanism illustrated in Scheme 2is believed to represent the mechanism by which compound **5** arises from the reaction of 3-chloro-quinoxaline derivative **7** with hydrazine.

**Scheme 3:**
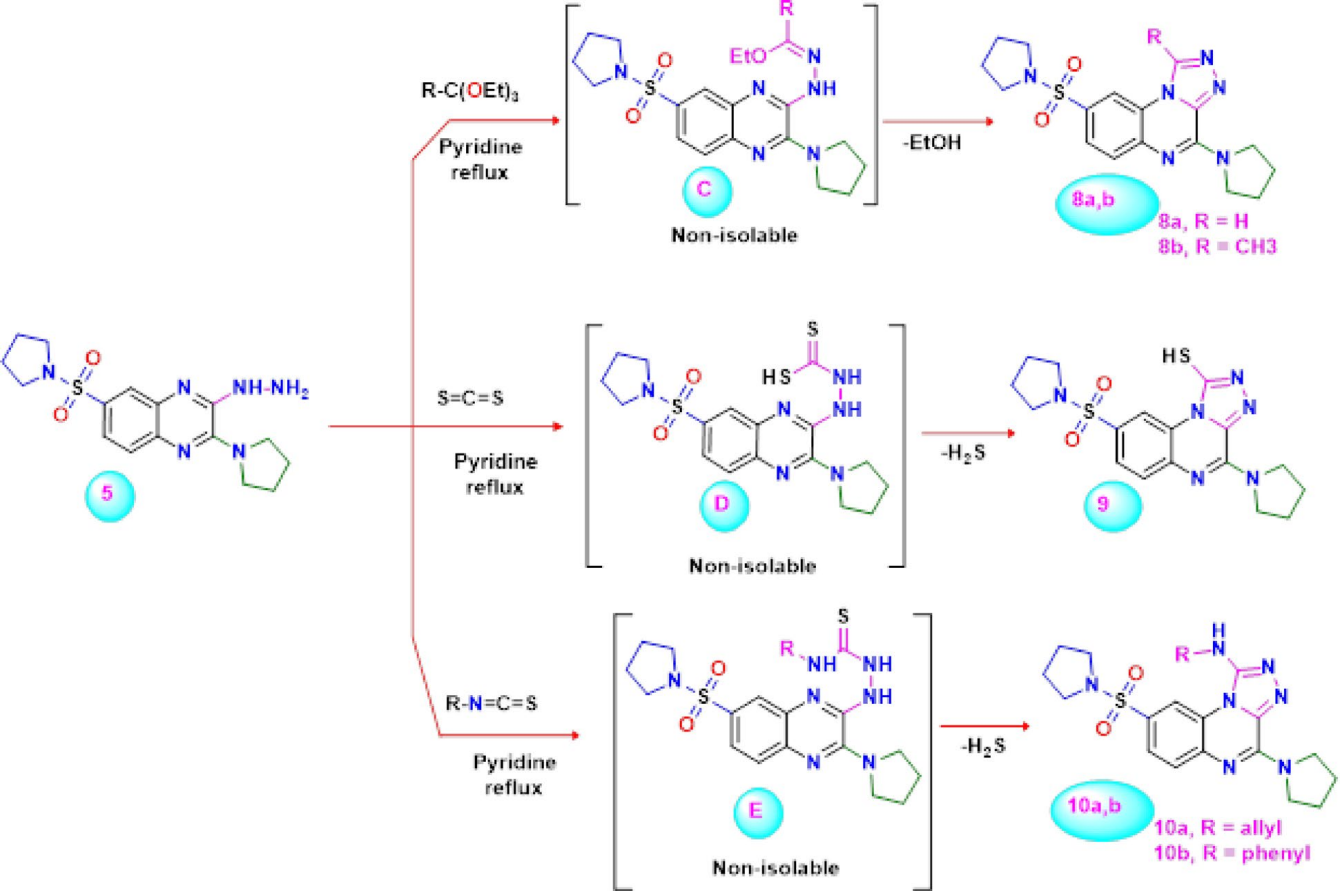
Synthesis of novel 4-(pyrrolidin-1-yl)-8-(pyrrolidin-1-ylsulfonyl)-[1,2,4]triazolo[4,3-a]quinoxaline derivatives** 8a**,** b**,** 9**, and** 10a**,** b**

Our current investigation was expanded to look at the reactions that the 3-hydrazino-quinoxaline derivative **5** has with different electrophiles. One of the most effective ways to generate annulated 1,2,4-triazole systems is through the reaction of hetarylhydrazines with orthoesters^63^. Thus, reaction of 3-hydrazino-quinoxaline derivative **5** with triethyl orthoformate or triethyl orthoacetate in refluxing pyridine afforded the desired products **8a**,** b** (Scheme 3). The molecular structure of the isolated product **8a**, as example, was confirmed based on its elemental analysis and spectral data. Also, the infrared spectrum of compound **8a** revealed the absence of the NH and NH_2_ absorption bands as well as the existence of the signature absorption bands for the CH-arom, CH-aliph, and C = N groups at 3039, 2988, and 1633 cm^− 1^, respectively. In addition, the^1^H NMR spectrum of the reaction product **8a** revealed a downfield singlet at *δ* 9.06 ppm assigned to the hydrogen attached at C_1_ of the [1,2,4]triazolo[4,3-*a*]quinoxaline ring. The^13^C NMR spectrum showed the following signals at *δ* 25.29 (4CH_2_.pyrrolidine), 48.52 (4 (N-CH_2_)pyrrolidine), 123.80, 126.00, 126.61, 126.97, 135.93, 139.34, 139.39, 145.03, and 159.48 (C = N). The formation of compound **8a** *via* cyclocondensation is assumed to proceed *via* the initial generation of iminoether intermediate **C** (R = H), which undergoes ring closure by losing the ethanol molecule to triazolo-quinoxaline **8a**.

Similarly, the novel 4-(pyrrolidin-1-yl)-8-(pyrrolidin-1-ylsulfonyl)-[1,2,4]triazolo[4,3-*a*]quinoxaline-1-thiol **9** was obtained in good yield by the ring closure reaction of the 3-hydrazino-quinoxaline **7** with carbon disulfide in refluxing pyridine. The structure of compound **9** was deduced on the basis of elemental analysis and spectral data. Also, the formation of 1,2,4-triazolo[4,3-*a*]quinoxaline-1-thiol **9** is assumed to be formed *via* the addition of the amino group of hydrazine to the activated double bond of carbon disulfide to form the intermediate dithiocarbamic acid **D**, which underwent cyclization with the elimination of hydrogen sulfide under the reaction conditions.

Furthermore, the novel 1,2,4-triazolo[4,3-*a*]quinoxalin-1-amine derivative **10a**,** b** was achieved in a *one-pot* reaction by the addition of 3-hydrazino-quinoxaline derivative **5** to allyl isothiocyanate or phenyl isothiocyanate under reflux in dry pyridine. The analytical and spectral data of the isolated products completely agreed with the structure of triazolo-quinoxaline **10a**,** b**. The infrared spectrum revealed absorption bands at 3228 and 1597 cm^− 1^ attributed to the NH and C = C groups, respectively. Its^1^H NMR spectrum showed two singlets for the protons of the pyrrolidine moieties at *δ* 1.68 and 3.19 ppm, a doublet for the protons of the *sp*^3^ carbon at *δ* 4.14 ppm, as well as multiples for the protons of the *sp*^2^ carbon and the proton of the carbon of methine at *δ* 5.20 and 5.88 ppm, respectively. The remaining signals represent aromatic and NH protons. Also, their^13^C NMR spectrum displayed signals at *δ* 25.67 (pyrrolidine-C), 47.03 (*sp*^3^ carbon), 49.32 (pyrrolidine -C), 102.90 (*sp*^2^ carbon), 117.62 (*sp*^2^ carbon, methine), 125.14, 129.60, 129.90, 130.29, 131.15, 135.61, 139.57, 143.49 ppm for the aromatic carbons and 155.54 related to the N = C.

**Scheme 4:**
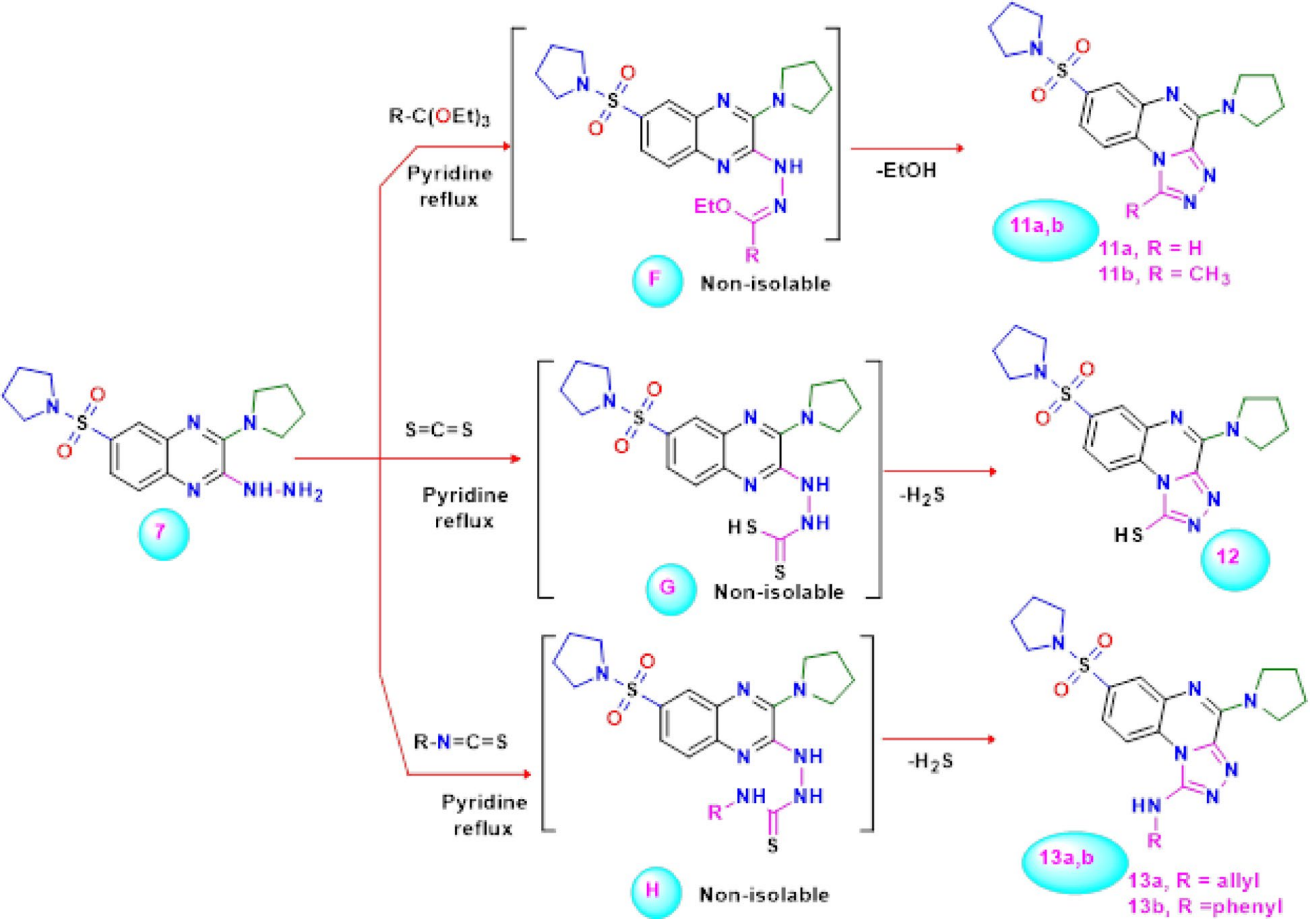
Synthesis of novel 4-(pyrrolidin-1-yl)-7-(pyrrolidin-1-ylsulfonyl)-[1,2,4]triazolo[4,3-a]quinoxaline derivatives** 11a**,** b**,** 12**, and** 13a**,** b**.

Furthermore, our current research has been extended to include a study of reactions between diverse electrophiles and the 2-hydrazino-quinoxaline derivative **7**. Additionally, reaction of the 2-hydrazino-quinoxaline derivative **7** with triethyl orthoformate or triethyl orthoacetate in refluxing pyridine gives rise to the products, designated as 4-(pyrrolidin-1-yl)-7-(pyrrolidin-1-ylsulfonyl)-[1,2,4]triazolo[4,3-*a*]quinoxaline **11a** and 1-methyl-4-(pyrrolidin-1-yl)-7-(pyrrolidin-1-ylsulfonyl)-[1,2,4]triazolo[4,3-*a*]quinoxaline **11b ** (Scheme 4). On the basis of analytical and spectral data, the molecular composition of the isolated products was confirmed. The NH and NH_2_ absorption bands disappeared from compound **11a** in infrared spectrum; however, it did have characteristic absorption bands for the CH-arom, CH-aliph, and C = N groups at 3028, 2964 and 1579 cm^-1^, respectively. The downfield singlet at *δ* 8.48 ppm in the^1^H NMR spectrum of the reaction product was attributed to the hydrogen linked at C_1_ of the [1,2,4]triazolo[4,3-*a*]quinoxaline ring. The formation of triazoloquinoxaline **11a** is assumed to proceed *via* the initial generation of iminoether intermediate **F** (R = H), which underwent ring closure by losing ethanol molecule to yield the corresponding **11a**. Similarly, the ring closure reaction of the 2-hydrazino-quinoxaline derivative **7** with carbon disulfide in pyridine at reflux temperature afforded the novel 4-(pyrrolidin-1-yl)-7-(pyrrolidin-1-ylsulfonyl)-[1,2,4]triazolo[4,3-*a*]quinoxaline-1-thiol **12** in good yield (Scheme 4). The triazolo-quinoxaline **12** structure was identified via spectroscopic and elemental evaluations. The formation of 1,2,4-triazolo[4,3-*a*]quinoxaline-1-thiol derivative **12** is assumed to be formed *via* the addition of the amino group of hydrazine to the activated double bond of carbon disulfide to create the intermediate dithiocarbamic acid **G**, which underwent cyclization with the elimination of hydrogen sulfide under the reaction conditions.

Finally, the addition of 2-hydrazino-quinoxaline derivative **7** to allyl isothiocyanate or phenyl isothiocyanate in dry pyridine under reflux led to the generation of the novel [1,2,4]triazolo[4,3-*a*]quinoxalin-1-amine derivative **13a**,** b** in *one pot* (Scheme 4). The microanalytical and spectral data of the isolated products were in complete agreement with the structure of triazolo-quinoxaline. The infrared spectrum of compound **13a** showed absorption bands at 3345 and 1595 cm^-1^ that were attributed to the NH and C = C groups, respectively. The^1^H NMR spectrum showed signals at *δ* 1.69 and 3.17 ppm for pyrrolidine moieties, a singlet signal at *δ* 4.23 ppm for the protons of the *sp*^3^ carbon, and multiple two signals at *δ* 5.28 and 5.88 ppm for the protons of the *sp*^2^ carbon and the proton of the carbon of methine, respectively. The^13^C NMR spectrum additionally revealed signals at *δ* 25.85 (pyrrolidine-CH_2_), 46.47 (*sp*^3^ carbon), 49.02 (pyrrolidine-N-CH_2_), 101.03 (*sp*^2^ carbon), 117.45 (*sp*^2^ carbon, methine), 122.62, 127.10, 128.04, 128.44, 132.12, 134.17, 135.92, 146.48, and 157.62 (N = C). The formation of triazoloquinoxaline **13a** is assumed to proceed via the addition of the amino group of hydrazine derivative **7** to the activated double bond in isothiocyanate **12a** to generate the non-isolable acyclic intermediate thiosemicarbazide **H**, which underwent cyclo-desulfurization under the reaction conditions to yield **13a** that its formation was confirmed by using a wet lead acetate paper which became black during the course reaction, suggesting the generation of hydrogen sulfide gas. Moreover, its^1^H NMR spectrum suggested that in addition to the expected signals, a downfield signal at *δ* 9.92 ppm designated for the NH group was also observed.

### Biological evaluation

####  In-vitro anti-diabetic activity with SAR study

The in vitro antidiabetic activity of the designed 1,2,4-triazoloquinoxaline **5–13** was evaluated by determining the inhibitory activity for α-amylase and α-glucosidase. The results of inhibitory percentage (IP) were tabulated in Table 1. Firstly, the synthesized derivatives were designed to be triazole-quinoxaline containing sulfonamide in two isomerism forms to determine which isomer exhibited the most activity. The two isomers containing the same functional groups (pyrrolidin-1-yl), substituted triazole, and hydrazine fragments, and changed in the position of the sulfonyl group as 6-(pyrrolidin-1-ylsulfonyl) for compounds (**5** and **7**) as starting material, as well as the desired products 8-(pyrrolidin-1-ylsulfonyl) for compounds **8–10** and 7-(pyrrolidin-1-ylsulfonyl) for compounds **11–13**.

**Table 1:** In vitro anti-diabetic and anti-Alzheimer activities of the designed hydazino-quinoxaline derivatives (5 and 7) and 1,2,4-triazolo-quinoxaline derivatives** 8-13**.

Compounds No.	The in vitro inhibitory percentage (IP ± SE) of the designed two isomers sulfonamide quinoxaline derivatives at 100 µM		
	Anti-diabetic activity ^a^		Anti-Alzheimer activity ^a^
	α-amylase	α-glucosidase	AChE
5	34.81 ± 0.01	37.60 ± 0.01	36.66 ± 0.01
7	36.17 ± 0.01	37.60 ± 0.01	38.61 ± 0.02
8a	50.46 ± 0.01	59.98 ± 0.01	17.16 ± 0.01
8b	18.42 ± 0.01	21.90 ± 0.03	14.01 ± 0.00
9	30.77 ± 0.01	39.49 ± 0.04	41.20 ± 0.01
10a	**64.70 ± 0.02**	**75.36 ± 0.01**	19.26 ± 0.01
10b	23.45 ± 0.01	25.75 ± 0.01	13.99 ± 0.01
11a	22.28 ± 0.00	24.07 ± 0.01	12.61 ± 0.01
11b	36.85 ± 0.01	39.64 ± 0.01	**44.78 ± 0.01**
12	21.85 ± 0.01	23.93 ± 0.01	12.44 ± 0.01
13a	21.94 ± 0.00	24.08 ± 0.00	12.42 ± 0.03
13b	21.90 ± 0.01	24.06 ± 0.00	12.61 ± 0.01
Acarbose	**67.33 ± 0.01**	**57.79 ± 0.01**	-
Donepezil	-	-	**67.27 ± 0.60**

^a^ Values were calculated from three replicates and expressed as mean ± SE

Significant values are in bold.

The best inhibitory activity of the designed derivatives compared to positive control drug used in this study.

The two starting materials, 3-hydrazino-2-(pyrrolidin-1-yl)quinoxaline derivative **5** and 2-hydrazino-3-(pyrrolidin-1-yl)quinoxaline derivative **7** contains the 6-(pyrrolidin-1-ylsulfonyl) exhibited close inhibitory activity against the tested enzymes. For 3-hydrazino-2-(pyrrolidin-1-yl)quinoxaline derivative **5** showed inhibitory percentage of 34.81 ± 0.01 and 37.60 ± 0.01% against α-amylase and α-glucosidase enzymes, while 2-hydrazino-3-(pyrrolidin-1-yl)quinoxaline derivative exhibited inhibitory percentage of 36.17 ± 0.01 and 37.60 ± 0.01% against α-amylase and α-glucosidase enzymes indicating that these two derivatives have the same effect on α-glucosidase enzyme and very close against amylase.

Generally, for the synthesized 4-(pyrrolidin-1-yl)-[1,2,4]triazolo[4,3-*a*]quinoxaline derivatives **8–13**, we found introducing the sulfonamide group to be 8-sulfonyl position in triazolo-quinoxaline scaffold exhibited better inhibitory activity than 7-sulfonyl that has the same nucleus and substituents, except for 1-methyl-4-(pyrrolidin-1-yl)-7-(pyrrolidin-1-ylsulfonyl)-[1,2,4]triazolo[4,3-*a*]quinoxaline **11b.** The 1,2,4-triazolo[4,3-*a*]quinoxaline **11b** revealed good inhibitory percentage against α-amylase (IP = 36.85 ± 0.01%) and α-glucosidase (IP = 39.64 ± 0.01%) compared the second isomer **8b** against α-amylase (IP = 18.42 ± 0.01%) and α-glucosidase (IP = 21.90 ± 0.03%) indicating electron donating group to triazole as methyl group enhancing the activity. On the other hand, replacing the hydrogen or methyl group at position one with thiol group causes enhancing the inhibitory activity in case of 8-sulfonyl-1,2,4triazoloquinoxaline derivative **9** with inhibitory percentage of against α-amylase (IP = 30.77 ± 0.01%) and α-glucosidase (IP = 39.49 ± 0.04%) rather than 8-sulfonyl-1,2,4triazoloquinoxaline derivative **12** with inhibitory percentage against α-amylase (IP = 21.85 ± 0.01%) and α-glucosidase (IP = 23.93 ± 0.01%), but still less than 4-(pyrrolidin-1-yl)-8-(pyrrolidin-1-ylsulfonyl)-[1,2,4]triazolo[4,3-*a*]quinoxaline (**8a**) α-amylase (IP = 50.46 ± 0.01%) and α-glucosidase (IP = 59.98 ± 0.01%). Additionally, the 4-(pyrrolidin-1-yl)-8-(pyrrolidin-1-ylsulfonyl)-[1,2,4]triazolo[4,3-*a*]quinoxaline (**8a**) exhibited the second most active member among the synthesized derivatives and exhibited that reaction of 3-hydrazino-quinoxaline derivative **5** with triethyl orthoformate and formation of triazolo-quinoxaline is preferred.

Introducing the amino group to position one of the 1,2,4-triazole nuclei enhances the activity in the case of the 8-sulfonyl group than the 7-sulfonyl group. Moreover, the presence of allyl group attached to the amino group in the case of *N*-allyl-4-(pyrrolidin-1-yl)-8-(pyrrolidin-1-ylsulfonyl)-[1,2,4]triazolo[4,3-*a*]quinoxalin-1-amine (**10a**) exhibited the best inhibitory activity with inhibitory percentage of 64.70 ± 0.02 and 75.36 ± 0.01% against α-amylase and α-glucosidase, respectively and compared to the designed derivatives. The *N*-allyl-4-(pyrrolidin-1-yl)-8-(pyrrolidin-1-ylsulfonyl)-[1,2,4]triazolo[4,3-*a*]quinoxalin-1-amine (**10a**) showed super inhibitory activity against α-glucosidase with inhibitory percentage value of 75.36 ± 0.01% compared to acarbose (IP = 57.79 ± 0.01%) with nearly 1.3-folds higher than positive control. On the other hand, *N*-allyl-4-(pyrrolidin-1-yl)- 1,2,4-triazolo[4,3-*a*]quinoxalin-1-amine derivative **10a** revealed good inhibitory activity with IP value of 64.70 ± 0.02% with a slightly lower activity than acarbose (IP = 67.33 ± 0.01%) against α-amylase. Replacing the allyl group with more hydrophobic moiety as phenyl group causes dropping in the activity as represented in *N*-phenyl-4-(pyrrolidin-1-yl)-8-(pyrrolidin-1-ylsulfonyl)-[1,2,4]triazolo[4,3-a]quinoxalin-1-amine (**10b**), where the inhibitory percentage values of 23.45 ± 0.01 for α-amylase and 25.75 ± 0.01% for α-glucosidase. Conversely, introducing the *N*-substituted-7-(pyrrolidin-1-ylsulfonyl)-triazolo[4,3-*a*]quinoxalin-1-aminederivatives **13a**,** b** don’t enhance the anti-diabetic activity against the two tested enzymes.

Our research was extended to evaluate the half maximal inhibitory concentration (IC_50_) for the most promising candidate **10a** and acarbose against α-amylase and α-glucosidase. The most active candidate, 1,2,4-triazolo[4,3-*a*]quinoxaline derivative **10a**, exhibited remarkable inhibitory activity with IC_50_ value of 3.46 ± 0.06 µM against α-glucosidase, in comparison to acarbose (IC_50_ = 4.27 ± 0.06 µM), representing approximately 123.4% of acarbose’s activity. Conversely, the most active derivative **10a** demonstrated favorable IC_50_ value of 6.89 ± 0.09 µM against α-amylase in comparison to acarbose (IC_50_ = 5.90 ± 0.09 µM), which represents approximately 85.63% of acarbose’s activity (Table 2).

**Table 2:** The median inhibitory concentrations IC50 (µM) of most active** 10a** and acarbose against the activities of α-amylase and α-glucosidase enzymes.

Compounds No.	The in vitro median inhibitory concentrations (IC_50_ ± SE) (µM)	
	Anti-diabetic activity ^a^	
	α-amylase	α-glucosidase
10a	6.89 ± 0.09	3.46 ± 0.06
Acarbose	5.90 ± 0.09	4.27 ± 0.06

^a^ Values were calculated from three replicates and expressed as mean ± SE

In conclusion, it can be confirmed that the 1,2,4-triazolo-quinoxaline demonstrates anti-diabetic activity by inhibiting α-amylase and α-glucosidase, with varying percentages of inhibition influenced by the substituents on the triazole nucleus, particularly at position one. Furthermore, the presence of the sulfonyl group serves as a significant contributor to the biological activity observed in the two isomers. Notably, the isomers 8-(pyrrolidin-1-ylsulfonyl)triazolo-quinoxaline derivatives **8–10** exhibited greater activity compared to the 8-(pyrrolidin-1-ylsulfonyl)[1,2,4]-triazolo-quinoxaline derivatives **11–13** indicating the introduction of hydrophobic pyrrolidinyl at position two as electron donating group and opposite to sulfonamide moiety at position eight is preferred. Moreover, the most promising candidate, **10a**, demonstrated a selective inhibition profile favoring α-glucosidase inhibition over α-amylase inhibition. This selectivity may represent a therapeutic advantage by presenting a more favorable safety and tolerability profile by mitigating gastrointestinal side effects, including flatulence, abdominal discomfort, and diarrhea, where these adverse effects primarily result from the accumulation of undigested carbohydrates in the colon, where they are fermented by gut microbiota, leading to gas production.

####  In-vitro anti-Alzheimer activity

**Fig. 2:**
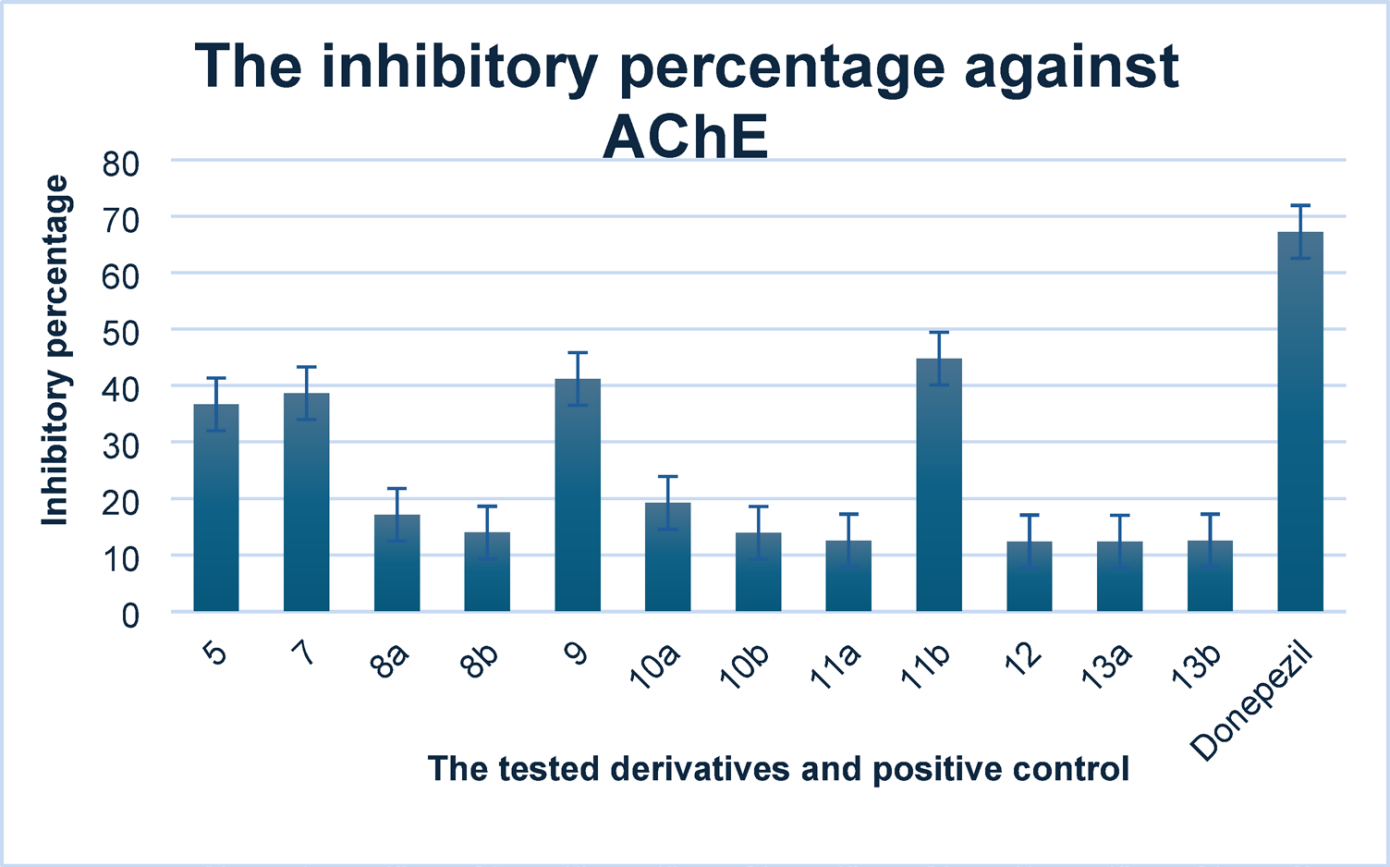
Graph represented the inhibitory percentage of the designed sulfonamide-quinoxaline against acetylcholinesterase (AChE).

Our work further examined the inhibitory effects of the designed two isomers sulfonamide-quinoxaline derivatives **5**,**7** and **8–13** on acetylcholinesterase (AChE), a target for Alzheimer’s disease, by comparing its inhibitory percentage to that of donepezil, which served as a positive control. Initially, the 3-hydrazino-6-sulfonyl quinoxaline derivative **5** and 2-hydrazinyl-6-sulfonyl quinoxaline derivative **7** showed inhibitory percentage values of 36.66 ± 0.01 and 38.61 ± 0.02%, respectively against acetylcholinesterase enzyme with slight differences between the two isomers (Table 1; Fig. 2). The results of the inhibitory activity of 1,2,4-triazolo-quinoxaline derivatives **8–13** can be represented as **11b** > **9** > **10a** > **8a** > **8b** > **10b** > **13b** = **11a** > **12** > **13a**, indicating cyclization of 3-hydrazino-quinoxaline derivative **5** to afford 8-(pyrrolidin-1-ylsulfonyl)-[1,2,4]triazolo[4,3-*a*]quinoxaline derivatives **8–10** formed most active derivatives than the other isomer 2-hydrazino-quinoxaline derivative **7** that afford 7-(pyrrolidin-1-ylsulfonyl)-[1,2,4]triazolo[4,3-*a*]quinoxaline derivatives **11–13**, except for triazolo-quinoxaline derivative **11b** that contain methyl group at C1 of [1,2,4]triazolo[4,3-*a*]quinoxaline derivative. The Structure-Activity Relationship (SAR) study of the most promising derivative, compound **11b**, can be elucidated further. Compound **11b** is characterized by the presence of a methyl group at the C1 position, in contrast to compound **9**, which incorporates a thiol group. The methyl group enhances hydrophobic interactions without introducing polar or reactive electronic effects, thereby likely improving both binding affinity and stability. Conversely, the thiol group in compound **9** may induce electronic repulsion or unfavorable interactions within the enzyme’s binding pocket, potentially compromising its inhibitory efficacy. Furthermore, in compound **11b**, the sulfonyl-pyrrolidine moiety at the 7-position likely aligns more favorably within the active site of acetylcholinesterase (AChE) compared to its placement at the 8-position, as seen in compound **9**. The proximity of the thiol in compound **9** may lead to steric clashes that distort the binding conformation or restrict access to critical residues.

The most active 1-methyl-4-(pyrrolidin-1-yl)-7-(pyrrolidin-1-ylsulfonyl)-[1,2,4]triazolo[4,3-*a*]quinoxaline (**11b**) revealed the highest inhibitory percentage with value of 44.78 ± 0.01% compared to all the designed derivatives and the second member is [1,2,4]triazolo[4,3-*a*]quinoxaline derivative **9** with IP = 41.20 ± 0.01%, but both of them still showed lower inhibitory percentage for donepezil as positive control drug (IP = 67.27 ± 0.60%). The other 1,2,4-triazolo[4,3-*a*]quinoxaline derivatives **8–13** demonstrated low to moderate inhibitory percentage with values ranging from 12.42 ± 0.03 to 17.16 ± 0.01% compared to 3-hydrazino-quinoxaline derivatives **5** (IP = 36.66 ± 0.01%) and **7** (IP = 38.61 ± 0.02%). Moreover, modification of the parent compound **11b** through the substitution of the C1 methyl group with bulkier substituents, specifically, *N*-allyl-1-amine in derivative **13a** and *N*-phenyl-1-amine in derivative **13b**, resulted in a notable decrease in inhibitory potency. Compounds **13a** and **13b** demonstrated reduced acetylcholinesterase inhibition, with IC₅₀ values recorded at 12.42 ± 0.03 µM and 12.61 ± 0.01%, respectively. This reduction in activity indicates that the introduction of larger substituents at this position may induce steric hindrance or negatively influence binding affinity at the active site of the enzyme. This observation underscores the sensitivity of enzyme interactions to subtle structural alterations within the quinoxaline framework.

In conclusion, the moderate activity exhibited by the designed derivatives against acetylcholinesterase suggests the necessity for further biological evaluations in subsequent research. This will facilitate exploring and assessing additional targets, including assays for Aβ aggregation inhibition and antioxidant studies.

###  In-silico toxicity prediction

The in-silico toxicity prediction is a crucial component of pharmaceutical research, enabling the early identification of potential toxicophoric compounds and reducing the risk of expensive late-stage failures. The most active derivatives of [1,2,4]triazolo[4,3-*a*]quinoxaline, specifically **10a** and **11b**, were evaluated for their toxicity using the virtual laboratory ProTox 3.0, as previously described^64,65^. The toxicity predictions for the most active derivatives **10a** and **11b** were conducted in comparison to the positive control drugs Acarbose and Donepezil as described in Table 3). The most promising derivatives, **10a** and **11b**, were predicted to be inactive concerning hepatotoxicity, cardiotoxicity, mutagenicity, ecotoxicity, nephrotoxicity, immunotoxicity, cytotoxicity, and nutritional toxicity. In contrast, the positive control Acarbose was predicted to have active properties related to hepatotoxicity, nephrotoxicity, cardiotoxicity, and immunotoxicity, with probability values ranging from 0.60 to 0.99. Conversely, Donepezil was expected to have a toxicity profile concerning cardiotoxicity, immunotoxicity, cytotoxicity, and ecotoxicity, with probability values of 0.50, 0.95, 0.63, and 0.56, respectively.

**Table 3:** In-silico toxicity prediction of most active [1,2,4]triazolo[4,3-a]quinoxaline derivatives 10a and 11b compared to positive control drugs (Acarbose and Donepezil).

Tested items	In-silico toxicity prediction of most active [1,2,4]triazolo[4,3-a]quinoxaline derivatives 10a and 11b compared to positive control drugs			
	10a	11b	Acarbose	Donepezil
LD_50_ (mg/kg)	465	250	24000	505
Toxicity class	4	3	6	4
Hepatotoxicity	Inactive 0.63	Inactive 0.69	Active 0.65	Inactive 0.98
Nephrotoxicity	Inactive 0.72	Inactive 0.77	Active 0.80	Inactive 0.67
Cardiotoxicity	Inactive 0.90	Inactive 0.92	Active 0.60	Active 0.50
Immunotoxicity	Inactive 0.95	Inactive 0.83	Active 0.99	Active 0.95
Mutagenicity	Inactive 0.70	Inactive 0.70	Inactive 0.76	Inactive 0.53
Cytotoxicity	Inactive 0.53	Inactive 0.59	Inactive 0.70	Active 0.63
Ecotoxicity	Inactive 0.61	Inactive 0.61	Inactive 0.66	Active 0.56
Nutritional toxicity	Inactive 0.62	Inactive 0.58	Inactive 0.52	Inactive 0.53

Finally, based on the obtained data, the most promising [1,2,4]triazolo[4,3-a]quinoxaline.

Furthermore, the most promising [1,2,4]triazolo[4,3-*a*]quinoxaline derivatives **10a** and **11b** were predicted to have LD_50_ values of 465 mg/kg and 250 mg/kg, respectively, indicating that these compounds belong to toxicity classes 4 and 3. In contrast, Acarbose belongs to class 6 with an LD_50_ of 24,000 mg/kg, while Donepezil is classified as class 4 (LD_50_ = 505 mg/kg).

Finally, based on the obtained data, the most promising [1,2,4]triazolo[4,3-*a*]quinoxaline derivatives **10a**jiand **11b** were predicted to demonstrate a more favorable safety profile in comparison to the positive control drugs acarbose and donepezil.

###  Molecular docking simulation

The molecular docking simulation was conducted to evaluate the binding affinities for the most active derivatives, *N*-allyl-4-(pyrrolidin-1-yl)-8-(pyrrolidin-1-ylsulfonyl)-[1,2,4]triazolo[4,3-*a*]quinoxalin-1-amine (**10a**) and acarbose on α-amylase (PDB: 2QV4) and α-glucosidase (PDB: 3W37) as anti-diabetic agents. Additionally, the highly active compound, 1-methyl-4-(pyrrolidin-1-yl)-7-(pyrrolidin-1-ylsulfonyl)-[1,2,4]triazolo[4,3-*a*]quinoxaline (**11b**) was docked within the active site of acetylcholinesterase (ACHE) (PDB: 4EY7), utilizing donepezil as a positive control.

**Fig. 3:**
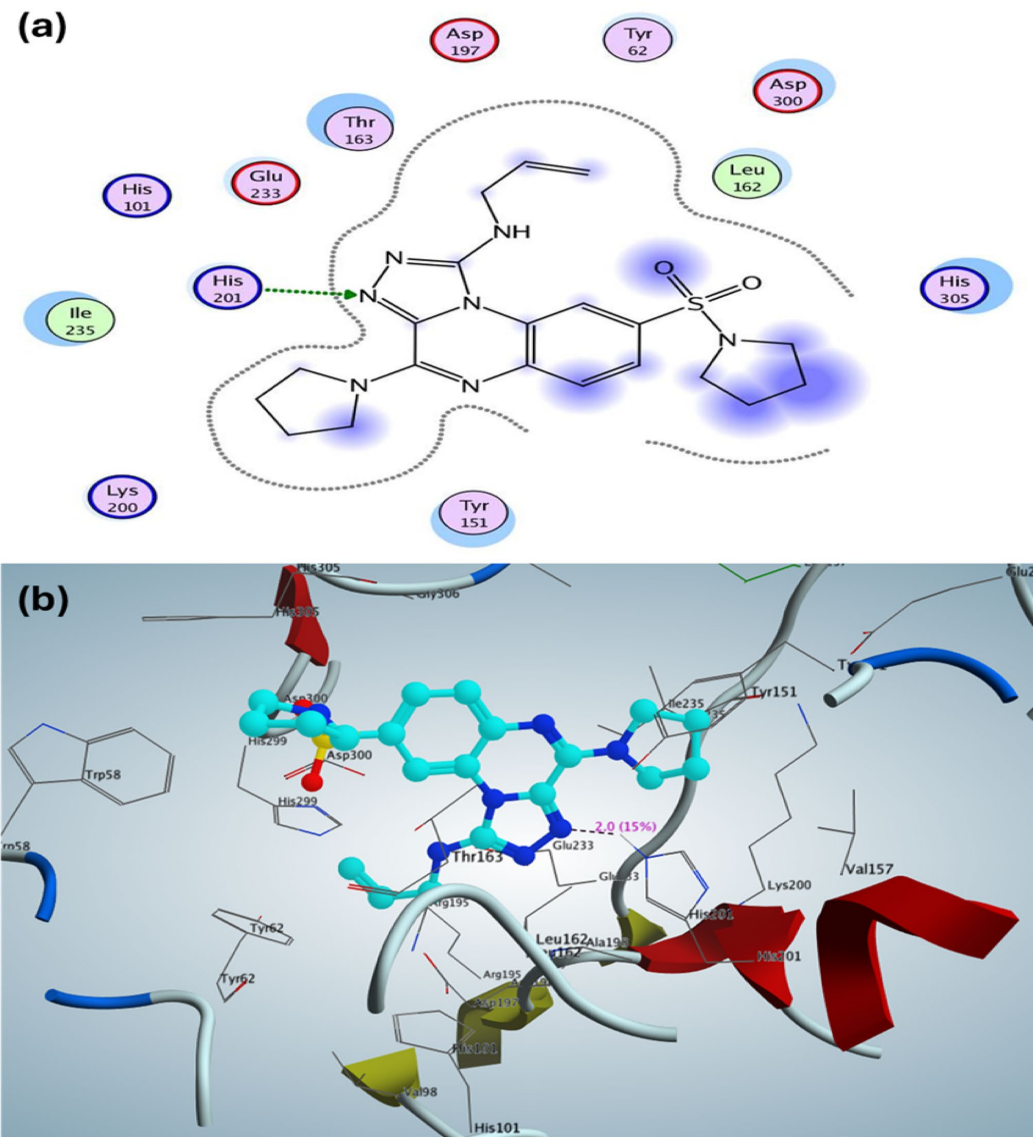
Graph represented the inhibitory percentage of the designed sulfonamide-quinoxaline against acetylcholinesterase (AChEGraph represented the inhibitory percentage of the designed sulfonamide-quinoxaline against acetylcholinesterase (AChE2D and 3D structure of the most active N-allyl-[1,2,4]triazolo[4,3-a]quinoxalin-1-aminederivative 10a inside the active site of α-amylase (PDB: 2QV4).

For α-amylase (PDB: 2QV4), the most active *N*-allyl-4-(pyrrolidin-1-yl)-8-(pyrrolidin-1-ylsulfonyl)-[1,2,4]triazolo[4,3-*a*]quinoxalin-1-amine (**10a**) exhibited a binding affinity (S) of -10.43 kcal/mol. This binding was characterized by the formation of one hydrogen bond between the side chain of His201 and the N_3_ atom of the triazole moiety, with a bond length of 2.0 Å and a bond strength of 15%. The *N*-allyl-[1,2,4]triazolo[4,3-*a*]quinoxalin-1-aminederivative **10a** also demonstrated hydrophobic interactions within the binding pocket, facilitated by the 4-pyrrolidine-1-yl, 8-(pyrrolidin-1-ylsulfonyl), and phenyl groups of quinoxaline, which are represented as blue regions in the 2D structural diagram. The hydrophobic interactions were illustrated with the following amino acid residues: Lys200, Tyr151, His305, Leu162, Asp300, Tyr62, Thr163, Glu233, His101, and Ile235 (Fig. 3a, b). The validation process indicated that the co-crystallized ligand, acarbose, demonstrated a binding affinity (S) of -16.33 kcal/mol with a root mean square deviation (RMSD) of 1.36 Å. Furthermore, acarbose exhibited an inhibitory percentage (IP) of 67.33 ± 0.01%, which correlated with the formation of various hydrogen bonds within the active site, including one hydrogen bond as a backbone acceptor with Thr163, one hydrogen bond as a sidechain donor with His201, and four hydrogen bonds as sidechain acceptors with the residues (two with Asp300, one with His299, and one with Glu233).

**Fig. 4:**
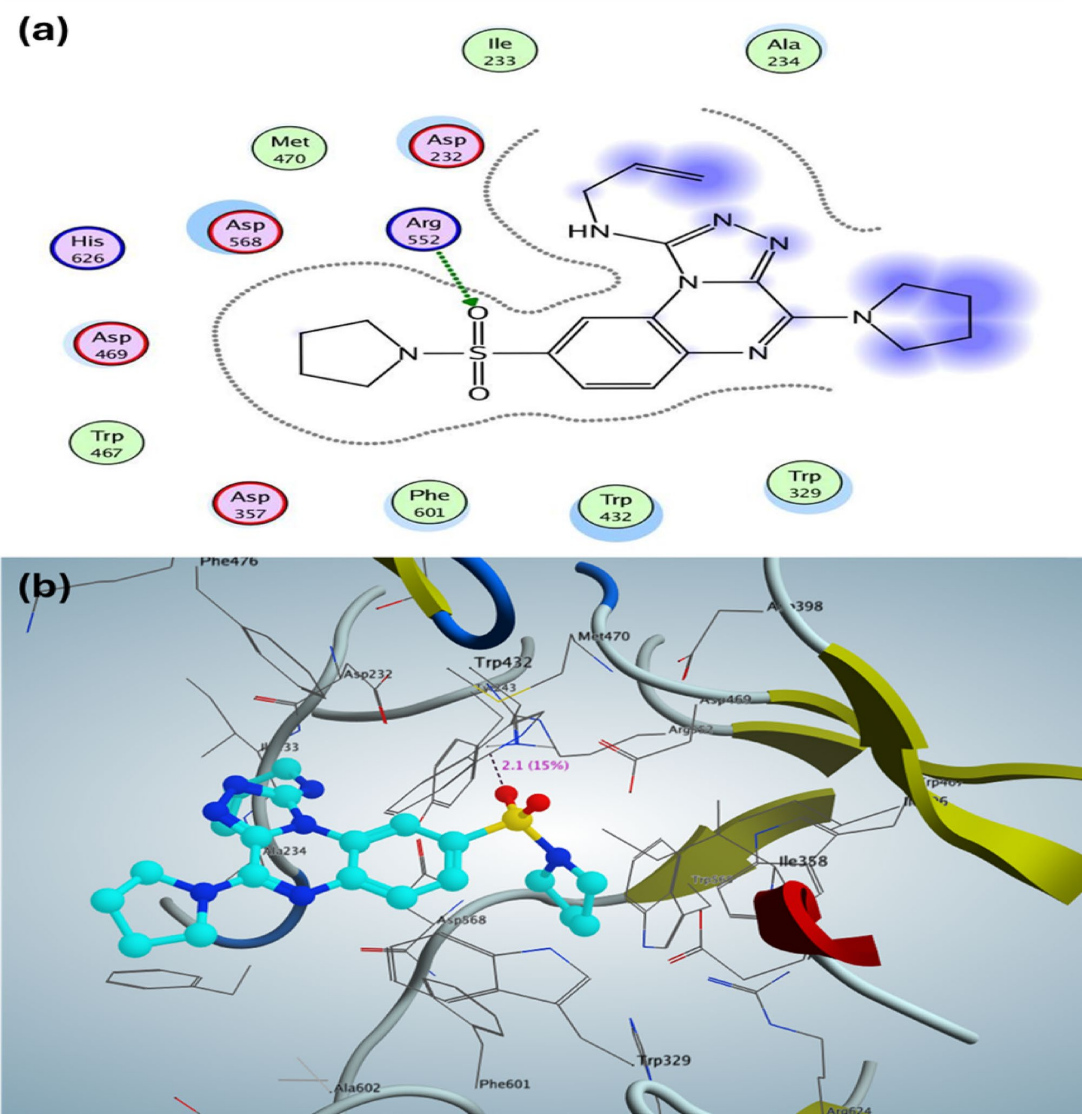
2D and 3D structure of the most active N-allyl-[1,2,4]triazolo[4,3-a]quinoxalin-1-aminederivative** 10a** inside the active site of α-glucosidase (PDB: 3W37).

For α-glucosidase (PDB: 3W37), the *N*-allyl-[1,2,4]triazolo[4,3-*a*]quinoxalin-1-amine derivative **10a** exhibited a binding affinity of S = -10.19 kcal/mol, which involved the formation of one hydrogen bond between the sulfonyl (SO_2_) oxygen and Arg552, characterized by a bond length of 2.1 Å and a bond strength of 15%. Furthermore, the positioning of the *N*-allyl-[1,2,4]triazolo[4,3-*a*]quinoxalin-1-amine derivative **10a** within the active site of the enzyme demonstrated hydrophobic interactions facilitated by three structural fragments: pyrrolidin-1-yl, triazole, and allyl. These fragments interacted with various amino acids, including Trp329, Trp432, Phe601, Asp357, Trp467, Asp469, His626, Asp568, Met470, Asp232, Ile233, and Ala234 (see Fig. 4a, b). In contrast, the validation process indicated that acarbose, utilized as a positive control, displayed a binding affinity of S = -16.82 kcal/mol with a root mean square deviation (RMSD) of 2.357 Å. Acarbose engaged with the binding pocket through the formation of seven hydrogen bond sidechain acceptors involving amino acids Asp232, Asp568, His625, and Asp357, as well as one hydrogen bond sidechain donor interaction with Asp552.

**Fig. 5:**
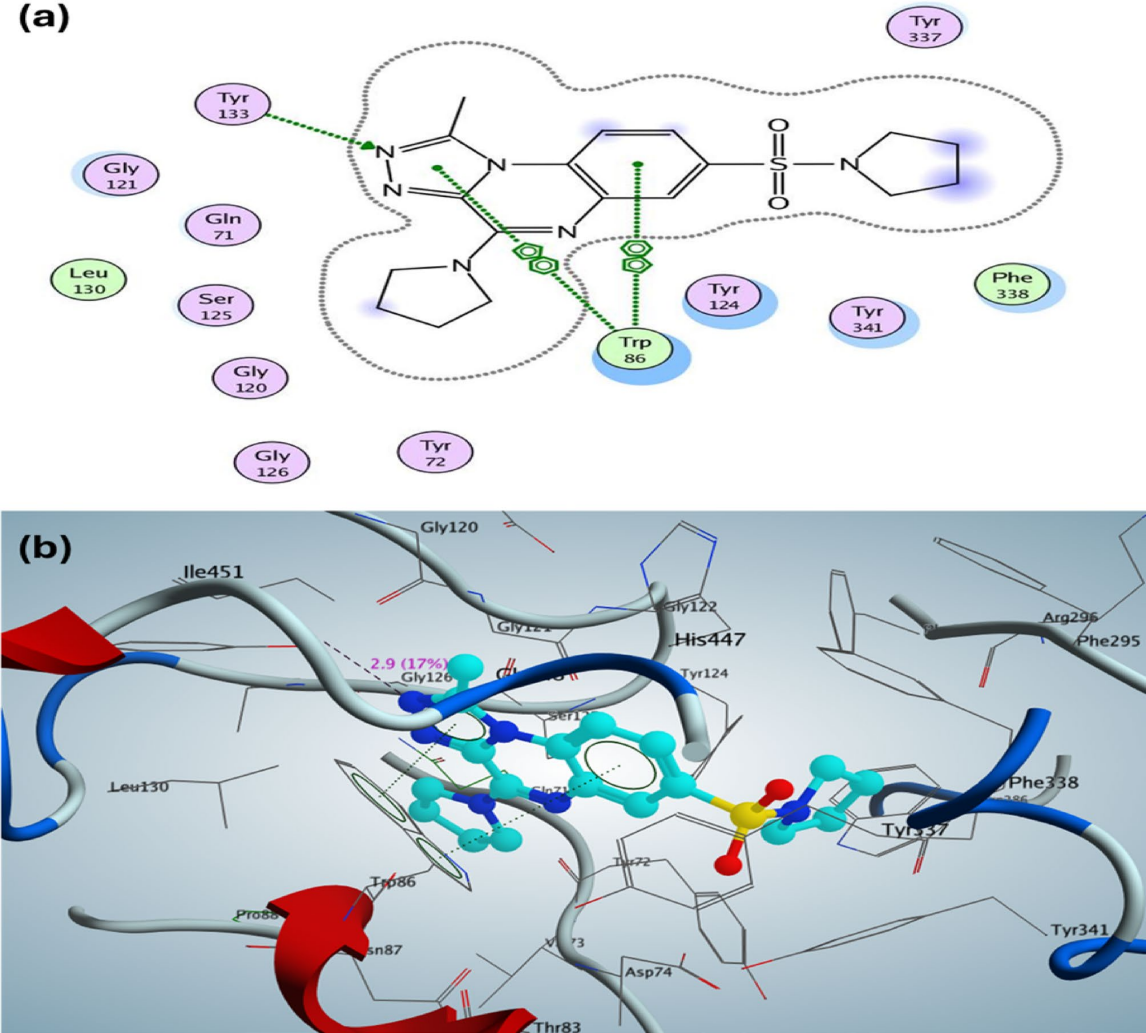
2D and 3D structure of the most active 1-methyl-4-(pyrrolidin-1-yl)-[1,2,4]triazolo[4,3-a]quinoxaline derivative** 11b** inside the active site of acetylcholinesterase (AChE) (PDB: 4EY7).

For acetylcholinesterase (AChE) (PDB: 4EY7), the docking pose of the most active 1-methyl-4-(pyrrolidin-1-yl)-[1,2,4]triazolo[4,3-*a*]quinoxaline derivative **11b** demonstrated a binding affinity (S) of -11.19 kcal/mol, in contrast to donepezil, which exhibited a binding affinity (S) of -11.027 kcal/mol as the co-crystallized ligand. The most active 1,2,4-triazolo[4,3-*a*]quinoxaline derivative **11b** appears to interact with the active site through one hydrogen bond involving a side-chain acceptor between Tyr133 and N_2_ of the triazolo-quinoxaline, with a bond length of 2.9 Å and a strength of 17%. Additionally, two arene-arene interactions were identified between the triazole and the phenyl group of quinoxaline with Trp86, while hydrophobic interactions were observed involving the sulfonyl pyrrolidine, the phenyl group of quinoxaline, and the pyrrolidine nucleus at C4 of the triazolo-quinoxaline scaffold (see Fig. 5a, b).

## Conclusion

In summary, this study presented the design and synthesis of novel 1,2,4-triazolo[4,3-*a*]quinoxaline derivatives featuring pyrrolidin-1-ylsulfonyl as a bioactive fragment, derived from hydrazio-6-(pyrrolidin-1-ylsulfonyl)quinoxaline derivatives **5** and **7**, utilizing a chemically regioselective synthesis approach. The structures of the synthesized 1,2,4-triazolo[4,3-*a*]quinoxaline derivatives were characterized and confirmed through various spectroscopic analyses, achieving favorable yields. The in vitro activities of these derivatives against α-amylase, α-glucosidase, and acetylcholinesterase (AChE) were evaluated and expressed as inhibitory percentages at a concentration of 100 µM. Generally, the 8-(pyrrolidin-1-ylsulfonyl) isomers **8–10** showed higher bio-evaluation than 7-(pyrrolidin-1-ylsulfonyl) isomer **11–13** cross the tested activities, with the exception of compound **11b** against AChE. The *N*-allyl-4-(pyrrolidin-1-yl)-8-(pyrrolidin-1-ylsulfonyl)-[1,2,4]triazolo[4,3-*a*]quinoxalin-1-amine derivative **10a** demonstrated exceptional inhibitory activity against α-glucosidase, with an inhibitory percentage of 75.36 ± 0.01%, surpassing that of acarbose (IP = 57.79 ± 0.01%) by nearly 1.3-fold. Conversely, the *N*-allyl-4-(pyrrolidin-1-yl)-1,2,4-triazolo[4,3-*a*]quinoxalin-1-amine derivative **10a** exhibited good inhibitory activity against α-amylase, with an inhibitory percentage of 64.70 ± 0.02%, which is slightly lower than acarbose (IP = 67.33 ± 0.01%). Furthermore, the tested derivatives demonstrated limited inhibition of acetylcholinesterase (AChE), with low inhibitory percentage values, with the exception of the 1-methyl-[1,2,4]triazolo[4,3-*a*]quinoxaline derivative **11b**, which exhibited an inhibitory percentage of 44.78 ± 0.01% compared to donepezil, the positive control drug (IP = 67.27 ± 0.60%). Additionally, the 1,2,4-triazolo[4,3-*a*]quinoxaline derivative **9** displayed the second most potent activity, with an inhibitory percentage of 41.20 ± 0.01%. By determining the IC_50_ values for most potent derivative **10a** as promising candidate for antidiabetic agent, we showed that it revealed remarkable inhibitory activity, with IC_50_ values of 3.46 ± 0.06 µM against α-glucosidase and 6.89 ± 0.09 µM against α-amylase. In comparison, acarbose has IC_50_ values of 4.27 ± 0.06 µM and 5.90 ± 0.09 µM for the same enzymes, respectively. The molecular docking simulation was conducted to elucidate the potential binding modes and affinities of the most active derivatives within the active sites of the enzymes compared to the positive control drugs acarbose and donepezil.

## Experimental

### Chemistry

### Materials and instrumentation

All reagents and chemicals were ordered from Aldrich Chemicals without further purification, and solvents from Fisher. Melting points (MPs) of all the newly designed compounds were recorded on a digital Gallen Kamp MFB-595 instrument using open capillaries. Within the range of 400–4000 cm^− 1^, IR spectra were calculated using the KBr disc methodology on a Shimadzu 440 spectrophotometer. In NMR spectra (^1^H / ^13^C), chemical shifts were calculated in *δ* ppm relative to TMS as an internal default (= 0 ppm) that obtained on a JOEL spectrometer 500 / 125 MHz using DMSO-*d*_*6*_ as solvents. The data was provided in the following format: chemical shift, multiplicity (br. = broad, m = multiplet, qu = quintet, q = quartet, t = triplet, d = doublet, and s = singlet), coupling constant (*J*) in Hertz (Hz), and integration. Elemental studies were carried out at Cairo University’s Micro Analytical Unit in Cairo. At Al-Azhar University’s Regional Center for Biotechnology, mass spectra were calculated at 70 eV using the DI-50 unit of a Shimadzu GC/MSQP5050A Spectrometer. Additionally, the 2,3-dioxo-1,2,3,4-tetrahydroquinoxaline-6-sulfonyl chloride **1** was prepared and reported according to the literature methods^66,67^, while 6-(pyrrolidin-1-ylsulfonyl)-1,4-dihydroquinoxaline-2,3-dione **2** was prepared and reported according to the literature methods^58,68,69^.

### Synthesis of organic materials

#### Synthesis of 2,3-dichloro-6-(pyrrolidin-1-ylsulfonyl)quinoxaline (3)

The key intermediate was prepared according to our previously work with slightly modification^58^. DMF (2 mL) was added drop by drop to a solution of 6-(pyrrolidin-1-ylsulfonyl)-1,4-dihydroquinoxaline-2,3-dione **2** (4 mmol) and POCl_3_ (20 mmol), the solution was stirred at 80 ºC for 6 h (monitored by TLC). After the reaction is completed, the solution is added portion wise to ice-water and neutralized with ammonia solution 30%. The formed precipitate was collected by filtration and crystallized from CH_3_CN to obtain the dichloro derivatives.

Grey powder (CH_3_CN); 85% yield; m.p. 190–192 ºC; **IR (KBr)**: ν_max_ = 3051(CH_**ar**_), 2970, 2875 (CH_**alip**_), 1615 (C = N), 1336, 1151 (SO_2_) cm^-1^;^1^**H NMR (δ**,** ppm)** = 1.63 (4 H, qu, (CH_2_)_2_-pyrrolidine), 3.26 (4 H, t, N(CH_2_)_2_-pyrrolidine), 8.22 (d, 1 H, *J* = 8.0 Hz, H_**7**_.quinox), 8.28 (d, 1 H, *J* = 8.0 Hz, H_**8**_.quinox), 8.42 (s, 1 H, H_**5**_.quinox)^13^. **C NMR (δ**,** ppm)** = 25.10 ((CH_2_)_2_-pyrrolidine), 48.31 (N(CH_2_)_2_-pyrrolidine), 125.99, 127.68, 130.03, 138.99, 139.53 (Ar.Cs), 141.78 (C-SO_2_), 147.09 (N = C-Cl), 147.77 (N = C-Cl); **Anal. Calcd** for **C**_**12**_**H**_**11**_**ClN**_**3**_**O**_**2**_**S** (332.20): C, 43.39; H, 3.34; N, 12.65; Found: C, 43.46; H, 3.58; N, 12.41.

#### Synthesis of 3-chloro-2-(pyrrolidin-1-yl)-6-(pyrrolidin-1-ylsulfonyl)quinoxaline (4)

To a solution of 2,3-dichloroquinoxaline sulfonamide derivatives **3** (1 mmol) and a secondary amine, such as pyrrolidine (1.5 mmol) in acetonitrile was stirred for 7 h until the complete consumption of the starting materials (monitored by TLC). After evaporation of the solvent, the resulting precipitate was washed with ethanol to remove the excess of secondary amine; it did not require any further purification.

Pale-yellow powder (CH_3_CN); 75% yield; m.p. 214–216 ºC; **IR (KBr)**: ν_max_ = 3040 (CH_**ar**_), 2965, 2835 (CH_**alip**_), 1639 (C = N), 1324, 1141 (SO_2_) cm^− 1^;^1^**H NMR (δ**,** ppm)** = 1.93–2.03 (8 H, m, (CH_2_)_4_-pyrrolidine), 3.73–3.75 (8 H, m, N(CH_2_)_4_-pyrrolidine), 7.73 (d, 1 H, *J* = 10.0 Hz, H_**7**_.quinox), 7.93 (d, 1 H, *J* = 10.0 Hz, H_**8**_.quinox), 8.07 (s, 1 H, H_**5**_.quinox);^13^**C NMR (δ**,** ppm)** = 25.28 ((CH_2_)_2_-pyrrolidine), 50.17 (N(CH_2_)_2_-pyrrolidine), 125.42, 127.90, 129.10, 130.29, 131.77, 135.93 (Ar.Cs), 144.59 (C-Cl), 160.57 (N = C-N); **Anal. Calcd** for **C**_**16**_**H**_**19**_**ClN**_**4**_**O**_**2**_**S** (366.86): C, 52.38; H, 5.22; N, 15.27; Found: C, 52.40; H, 5.20; N, 15.30.

#### Synthesis of 3-hydrazino-2-(pyrrolidin-1-yl)-6-(pyrrolidin-1-ylsulfonyl)quinoxaline (5)

To a mixture of compound **4** (1 mmol) in absolute ethanol and the appropriate hydrazine hydrate (100%) (1 mmol) was heated under reflux for 3 h at 120 ^O^C, until the reaction completed (monitored by TLC), the solid product was precipitated on hot, collected, washed with ethanol and then recrystallized from acetonitrile to produce the following pure compound.

Orange powder (CH_3_CN); 67% yield; m.p. 250–252 ºC; **IR (KBr)**: ν_max_ = 3499, 3315, 3185 (NH_2_, NH), 2950 (br.CH_**alip**_), 1606 (C = N), 1325, 1136 (SO_2_);^1^**H NMR (*δ***, **ppm)** = 1.60–1.63 (8 H, m, (CH_2_)_4_-pyrrolidine), 3.07 (8 H, t, N(CH_2_)_4_-pyrrolidine), 7.00 (br. s, 3 H, NH_2_ + NH, exchangeable with D_2_O), 7.35 (d, 2 H, *J* = 8.4 Hz, H_**7**_.quinox, H_**8**_.quinox), 7.53 (s, 1 H, H_**5**_.quinox);^13^**C NMR (*δ***, **ppm)** 26.56 ((CH_2_)_2_-pyrrolidine), 54.76 (N(CH_2_)_2_-pyrrolidine), 125.13, 128.02, 129.62, 131.35, 133.55, 138.05, 147.21 (Ar.Cs), 156.60 (N = C-NH); **Anal. Calcd** for **C**_**16**_**H**_**22**_**N**_**6**_**O**_**2**_**S** (362.45): C, 53.02; H, 6.12; N, 23.19; Found: C, 53.05; H, 6.14; N, 23.21.

#### Synthesis of 3-chloro-2-hydrazino-6-(pyrrolidin-1-ylsulfonyl)quinoxaline (6)

To a mixture of the starting material **3** (1 mmol) in absolute ethanol and the appropriate hydrazine hydrate (100%) (1 mmol) was added portion-wise for 15 min. Until the addition is completed, the solution was stirred at room temperature for 3 h, (monitored by TLC). After the reaction is completed, the new product was precipitated, collected, washed with ethanol and then recrystallized from 1,4-dioxane to produce the following pure compound.

Light-orange powder (1,4-dioxane); 80% yield; m.p. 200–202 ºC; **IR (KBr)**: ν_max_ = 3429, 3380, 3254 (NH_2_, NH), 3082 (CH_**ar**_), 2990, 2946, 2891 (CH_**alip**_), 1642 (C = N), 1337, 1126 (SO_2_);^1^**H NMR (δ**,** ppm)** = 1.72–1.91 (4 H, m, ((CH_2_)_2_-pyrrolidine), 3.63–3.73 (4 H, m, N(CH_2_)_2_-pyrrolidine), 7.31 (br. s, 2 H, NH_2_, exchangeable with D_2_O), 7.43 (br. s, 1 H, NH, exchangeable with D_2_O), 7.90 (d, 1 H, J = 8.2 Hz, H7.quinox), 7.95–8.02 (m, 2 H, H8.quinox, H5.quinox);^13^**C NMR (δ**,** ppm)** = 25.57 ((CH_2_)_2_-pyrrolidine), 49.12 (N(CH_2_)_2_-pyrrolidine), 125.21, 126.36, 127.77, 129.14, 133.32, 137.99 (Ar.Cs), 143.79 (C-Cl), 153.77 (N = C-NH); **Anal. Calcd** for **C**_**12**_**H**_**14**_**ClN**_**5**_**O**_**2**_**S** (327.79): C, 43.97; H, 4.31; N, 21.37; Found: C, 43.78; H, 4.23; N, 21.59.

#### Synthesis of 2-hydrazino-3-(pyrrolidin-1-yl)-6-(pyrrolidin-1-ylsulfonyl)quinoxaline (7)

To a solution of compound **6** (1 mmol) and a secondary amine (pyrrolidine) (1.5 mmol) in absolute ethanol was heated under reflux for 3 h at 120 ºC, until the reaction completed (monitored by TLC), the solid product was precipitated on hot, collected, washed with ethanol and then recrystallized from acetonitrile to produce the following pure compound.

Deep-orange powder (CH_3_CN); 86% yield; m.p. 244–246 ºC; **IR (KBr)**: ν_max_ = 3422 (br. NH_2_, NH), 3051(CH_**ar**_), 2980, 2759 (CH_**alip**_), 1598 (C = N), 1338, 1132 (SO_2_) cm^-1^;^1^**H NMR (*δ***, **ppm)** = 1.64 (8 H, s, (CH_2_)_4_-pyrrolidine), 3.17 (4 H, s, N(CH_2_)_2_-pyrrolidine), 3.45 (4 H, s, N(CH_2_)_2_-pyrrolidine), 7.31 (br. s, 2 H, NH_2_, exchangeable with D_2_O), 7.43 (br. s, 1 H, NH, exchangeable with D_2_O), 7.64 (d, 1 H, *J* = 8.8 Hz, H_**7**_.quinox), 7.76–7.84 (m, 2 H, H_**8**_.quinox, H_**5**_.quinox);^13^**C NMR (*δ***, **ppm)** = 25.25 ((CH_2_)_2_-pyrrolidine), 50.12 (N(CH_2_)_2_-pyrrolidine), 54.78 (N(CH_2_)_2_-pyrrolidine), 122.44, 125.54, 127.48, 129.21, 129.94, 135.96, 143.01 (Ar.Cs), 160.73 (N = C-NH); **Anal. Calcd** for **C**_**16**_**H**_**22**_**N**_**6**_**O**_**2**_**S** (362.45): C, 53.02; H, 6.12; N, 23.19; Found: C, 53.15; H, 6.18; N, 23.01.

#### Synthesis of 1-substituted-4-(pyrrolidin-1-yl)-8-(pyrrolidin-1-ylsulfonyl)-[1,2,4]triazolo[4,3-a]quinoxaline (8a, b)

To a solution of 3-hydrozienylquinoxaline derivatives **5** (1 mmol) and either triethyl orthoformate (10 mmol) or triethyl orthoacetate (10 mmol) was stirred at 100 ºC for 1 h (monitored by TLC). After the reaction is cooled, the solid product was precipitated, collected, washed ethanol and then recrystallized from acetonitrile to produce the following pure compounds.

#### 4-(Pyrrolidin-1-yl)-8-(pyrrolidin-1-ylsulfonyl)-[1,2,4]triazolo[4,3-*a*]quinoxaline (8a)

Brown powder (CH_3_CN); 79% yield; m.p. 344–346 ºC; **IR (KBr)**: ν_max_ = 3039 (CH_**ar**_), 2988, 2880 (CH_**alip**_), 1633 (C = N), 1334, 1196 (SO_2_) cm^-1^;^1^**H NMR (δ**,** ppm)** = 1.65 (8 H, s, (CH_2_)_4_-pyrrolidine), 3.22 (4 H, s, N(CH_2_)_2_-pyrrolidine), 3.27 (4 H, s, N(CH_2_)_2_-pyrrolidine),8.08 (1 H, d, *J* = 8.8 Hz, H_8_. quinox), 8.65 (1 H, d, *J* = 8.8 Hz, H_7_. quinox), 8.85 (1 H, s, H_5_. quinox), 9.06 (1 H, s, CH. triazole)^13^. **C NMR (δ**,** ppm)** = 25.29 (4CH_2_.pyrrolidine), 48.52 (4 N-CH_2_.pyrrolidine), 123.80, 126.00, 126.61, 126.97, 135.93, 139.34, 139.39, 145.03 (Ar.CS), 159.48 (N = C-N); **Anal. Calcd** for **C**_**17**_**H**_**20**_**N**_**6**_**O**_**2**_**S** (372.45): C, 54.82; H, 5.41; N, 22.56; Found: C, 54.75; H, 5.23; N, 22.68.

#### 1-Methyl-4-(pyrrolidin-1-yl)-8-(pyrrolidin-1-ylsulfonyl)-[1,2,4]triazolo[4,3-*a*]quinoxaline (8b)

Light-brown powder (CH_3_CN); 72% yield; m.p. 310–312 ºC; **IR (KBr)**: ν_max_ = 3049(CH_**ar**_), 2978, 2892 (CH_**alip**_), 1633 (C = N), 1337, 1196 (SO_2_) cm^-1^;^1^**H NMR (*δ***, **ppm)** = 1.63 (8 H, qui, (CH_2_)_4_-pyrrolidine), 2.00 (3 H, s, CH_3_.triazole), 3.09 (4 H, s, N(CH_2_)_2_-pyrrolidine), 3.10 (4 H, t, N(CH_2_)_2_-pyrrolidine), 7.79 (1 H, d, *J* = 6.0 Hz, H_8_. quinox), 8.08 (1 H, d, *J* = 8.8 Hz, H_7_. quinox), 8.50 (1 H, s, H_5_. quinox)^13^. **C NMR (*δ***, **ppm)** = 14.48 (CH_3_. Triazole), 25.32 ((CH_2_)_2_-pyrrolidine), 48.25 (N(CH_2_)_2_-pyrrolidine), 122.78, 125.09, 126.31, 127.41, 130.77, 133.18, 134.96, 143.53, 148.42 (Ar.CS), 157.82 (N = C-N); **Anal. Calcd** for **C**_**18**_**H**_**22**_**N**_**6**_**O**_**2**_**S** (386.47): C, 55.94; H, 5.74; N, 21.75; Found: C, 55.95; H, 5.76; N, 21.77.

#### Synthesis of 4-(pyrrolidin-1-yl)-8-(pyrrolidin-1-ylsulfonyl)-[1,2,4]triazolo[4,3-*a*]quinoxaline-1-thiol (9).

To a solution of compound **5** (1 mmol) and carbon disulphide (1 mmol) in pyridine (15 mL) as solvent and catalyst, the solution mixture was refluxed (until the evolution of H_2_S finished). The reaction mixture was then poured onto cold water, and the solid product precipitated was collected by filtration, dried, and recrystallized from 1,4-dioxane.

Deep-orange powder (1,4-dioxane); 84% yield; m.p. 288–290 ºC; **IR (KBr)**: ν_max_ = 3041 (CH_**ar**_), 2945, 2886 (CH_**alip**_), 1633 (C = N), 1317, 1197 (SO_2_) cm^-1^;^1^**H NMR (*δ***, **ppm)** = 1.69 (8 H, s, (CH_2_)_4_-pyrrolidine), 3.27 (8 H, s, N(CH_2_)_4_-pyrrolidine), 7.90 (1 H, d, *J* = 7.6 Hz, H_8_. quinox), 8.06 (1 H, d, *J* = 9.6 Hz, H_7_. quinox), 8.62 (1 H, s, H_5_. quinox), 11.11 (1 H, s, SH, D_2_O exchangeable)^13^. **C NMR (*δ***, **ppm)** = 25.28 ((CH_2_)_2_-pyrrolidine), 48.42 (N(CH_2_)_2_-pyrrolidine), 124.84, 126.00, 129.04, 134.37, 137.28, 138.02, 149.05 (Ar.Cs), 164.13 (N = C-N), 164.64 (N = C-SH); **Anal. Calcd** for **C**_**17**_**H**_**20**_**N**_**6**_**O**_**2**_**S**_**2**_ (404.51): C, 50.48; H, 4.98; N, 20.78; Found: C, 50.77; H, 4.76; N, 20.60.

#### Synthesis of N-substituted-4-(pyrrolidin-1-yl)-8-(pyrrolidin-1-ylsulfonyl)-[1,2,4]triazolo[4,3-***a***]quinoxalin-1-amine (10a, b).

To a solution of compound **5** (1 mmol) and derivatives of isothiocyanate (1 mmol) namely, ally isothiocyanate and / or phenyl isothiocyanate in pyridine (15 mL) as solvent and catalyst, the solution mixture was refluxed (until the evolution of H_2_S finished). The reaction mixture was then poured onto cold water, and the solid product precipitated was collected by filtration, dried, and recrystallized from 1,4-dioxane.

#### ***N***-Allyl-4-(pyrrolidin-1-yl)-8-(pyrrolidin-1-ylsulfonyl)-[1,2,4]triazolo[4,3-***a***]quinoxalin-1-amine (10a)

Brown powder (1,4-dioxane); 80% yield; m.p. 305–307 ºC; **IR (KBr)**: ν_max_ = 3041(CH_**ar**_), 2945, 2922 (CH_**alip**_), 1634 (C = N), 1331, 1197 (SO_2_) cm^-1^;^1^**H NMR (δ**,** ppm)** = 1.68 (8 H, s, (CH_2_)_4_-pyrrolidine), 3.19 (8 H, s, N(CH_2_)_4_-pyrrolidine), 4.14 (2 H, d, CH_2_-CH.allyl), 5.20 (2 H, m, CH_2_ = CH-allyl), 5.88 (1 H, s, CH = CH_2_.allyl), 6.09 (1 H, s, NH, D_2_O exchangeable), 7.62 (1 H, d, *J* = 7.2 Hz, H_8_. quinox), 8.06–8.21 (1 H, m, H_7_. quinox), 8.70 (1 H, s, H_5_. quinox)^13^. **C NMR (δ**,** ppm)** = 25.67 ((CH_2_)_2_-pyrrolidine), 47.03 (CH_2_-CH.allyl), 49.32(N(CH_2_)_2_-pyrrolidine), 102.90 (CH_2_ = CH. allyl), 117.62 (CH_2_ = CH. allyl), 125.14, 129.60, 129.90, 130.29, 131.15, 135.61, 139.57, 143.49 (Ar.Cs), 155.54 (N = C-N); **Anal. Calcd** for **C**_**20**_**H**_**25**_**N**_**7**_**O**_**2**_**S** (427.53): C, 56.19; H, 5.89; N, 22.93; Found: C, 56.10; H, 5.99; N, 22.75.

#### ***N***-Phenyl-4-(pyrrolidin-1-yl)-8-(pyrrolidin-1-ylsulfonyl)-[1,2,4]triazolo[4,3-***a***]quinoxalin-1-amine (10b)

Brown powder (1,4-dioxane); 85% yield; m.p. 319–320 ºC; **IR (KBr)**: ν_max_ = 3028(CH_**ar**_), 2945, 2885 (CH_**alip**_), 1597 (C = N), 1338, 1197 (SO_2_) cm^-1^;^1^**H NMR (δ**,** ppm)** = 1.69 (8 H, s, (CH_2_)_4_-pyrrolidine), 3.27 (8 H, s, N(CH_2_)_4_-pyrrolidine), 6.92 (1 H, t, *J* = 7.2 Hz, ph-H), 7.28 (2 H, t, *J* = 8.8 Hz, ph-H), 7.39–7.41 (2 H, m, ph-H), 7.55 (1 H, d, *J* = 8.0 Hz, H_8_. quinox), 7.78 (1 H, dd, *J* = 7.6, 2.0 Hz, H_7_. quinox), 8.05 (1 H, d, *J* = 8.8 Hz, H_5_. quinox), 11.24(1 H, s, NH, D_2_O exchangeable*).*^13^**C NMR (δ**,** ppm)** = 25.36 ((CH_2_)_2_-pyrrolidine), 48.50 (N(CH_2_)_2_-pyrrolidine), 115.37, 117.13, 121.48, 124.44, 126.44, 128.90, 129.04, 129.87, 134.37, 136.79, 137.30, 141.67, 149.93, 156.14 (Ar.Cs), 164.34 (N = C-N); **Anal. Calcd** for **C**_**23**_**H**_**25**_**N**_**7**_**O**_**2**_**S** (463.56): C, 59.59; H, 5.44; N, 21.15; Found: C, 59.50; H, 5.26; N, 21.10.

#### Synthesis of 1-substituted-4-(pyrrolidin-1-yl)-7-(pyrrolidin-1-ylsulfonyl)-[1,2,4]triazolo[4,3-a]quinoxaline (11a, b)

To a solution of compound **7** (1 mmol) and either triethyl orthoformate (10 mmol) or triethyl orthoacetate (10 mmol) was stirred at 100 ºC for 1 h (monitored by TLC). After the reaction is cooled, the solid product was precipitated, collected, washed ethanol and then recrystallized from acetonitrile to produce the following pure compounds.

#### 4-(Pyrrolidin-1-yl)-7-(pyrrolidin-1-ylsulfonyl)-[1,2,4]triazolo[4,3-*a*]quinoxaline (11a)

Brown powder (CH_3_CN); 69% yield; m.p. 336–338 ºC; **IR (KBr)**: ν_max_ = 3028(CH_**ar**_), 2964, 2878 (CH_**alip**_), 1597 (C = N), 1335, 1198 (SO_2_) cm^-1^ ;^1^**H NMR (δ**,** ppm)** = 1.68 (8 H, s, (CH_2_)_4_-pyrrolidine), 3.17–3.23 (8 H, m, N(CH_2_)_4_-pyrrolidine), 7.75 (1 H, d, *J* = 6.4 Hz, H_8_. quinox), 8.20 (1 H, d, *J* = 8.0 Hz, H_7_. quinox), 8.37 (1 H, s, H_5_. quinox), 8.48 (1 H, s, *CH.* Triazole*).*^13^**C NMR (δ**,** ppm)** = 25.25 ((CH_2_)_2_-pyrrolidine), 48.40 (N(CH_2_)_2_-pyrrolidine), 120.94, 125.10, 126.10, 129.76, 139.51, 140.90, 141.83, 146.21 (Ar.Cs), 168.34 (N = C-N); **Anal. Calcd** for **C**_**17**_**H**_**20**_**N**_**6**_**O**_**2**_**S** (372.45): C, 54.82; H, 5.41; N, 22.56; Found: C, 54.65; H, 5.54; N, 22.77.

#### 1-Methyl-4-(pyrrolidin-1-yl)-7-(pyrrolidin-1-ylsulfonyl)-[1,2,4]triazolo[4,3-*a*]quinoxaline (11b)

Light-brown powder (CH_3_CN); 65% yield; m.p. 324–326 ºC; **IR (KBr)**: ν_max_ = 3028(CH_**ar**_), 2968, 2874 (CH_**alip**_), 1597 (C = N), 1337, 1198 (SO_2_) cm^-1^ ;^1^**H NMR (δ**,** ppm)** = 1.63 (8 H, s, (CH_2_)_4_-pyrrolidine), 1.92(3 H, s, CH_3_.triazole), 3.13–3.21 (8 H, m, N(CH_2_)_4_-pyrrolidine), 7.25–7.32 (1 H, m, H_8_. quinox), 7.64 (1 H, d, *J* = 8.4 Hz, H_7_. quinox), 7.72 (1 H, s, H_5_. quinox*).*^13^**C NMR (δ**,** ppm)** = 17.02 (CH_3_.triazole), 25.12 (4CH_2_.pyrrolidine), 48.49 (4 N-CH_2_.pyrrolidine), 122.78, 126.31, 127.41, 133.18, 140.71, 144.11, 147.81, 149.28 (Ar.Cs), 157.82 (N = C-N); **Anal. Calcd** for **C**_**18**_**H**_**22**_**N**_**6**_**O**_**2**_**S** (386.47): C, 55.94; H, 5.74; N, 21.75; Found: C, 55.76; H, 5.85; N, 21.57.

#### Synthesis of 4-(pyrrolidin-1-yl)-7-(pyrrolidin-1-ylsulfonyl)-[1,2,4]triazolo[4,3-***a***]quinoxaline-1-thiol (12)

To a solution of compound **7** (1 mmol) and carbon disulphide (1 mmol) in pyridine (15 mL) as solvent and catalyst, the solution mixture was refluxed (until the evolution of H_2_S finished). The reaction mixture was then poured onto cold water, and the solid product precipitated was collected by filtration, dried, and recrystallized from 1,4-dioxane.

Brown powder (1,4-dioxane); 77% yield; m.p. 310–312 ºC; **IR (KBr)**: ν_max_ = 3028(CH_**ar**_), 2971, 2878 (CH_**alip**_), 1597 (C = N), 1333, 1153 (SO_2_) cm^-1^;^13^**C NMR (δ**,** ppm)** = 25.36 ((CH_2_)_2_-pyrrolidine), 48.50 (N(CH_2_)_2_-pyrrolidine), 122.67, 126.45, 129.04, 134.94, 137.31, 139.21, 147.60 (Ar.Cs), 164.64 (N = C-N), 167.45 (N = C-SH); **Anal. Calcd** for **C**_**17**_**H**_**20**_**N**_**6**_**O**_**2**_**S**_**2**_ (404.51): C, 50.48; H, 4.98; N, 20.78; Found: C, 50.60; H, 4.79; N, 20.81.

#### Synthesis of ***N***-substituted-4-(pyrrolidin-1-yl)-7-(pyrrolidin-1-ylsulfonyl)-[1,2,4]triazolo[4,3-a]quinoxalin-1-amine (13a, b)

To a solution of compound **7** (1 mmol) and derivatives of isothiocyanate (1 mmol) namely, ally isothiocyanate and / or phenyl isothiocyanate in pyridine (15 mL) as solvent and catalyst, the solution mixture was refluxed (until the evolution of H_2_S finished). The reaction mixture was then poured onto cold water, and the solid product precipitated was collected by filtration, dried, and recrystallized from 1,4-dioxane.

#### ***N***-Allyl-4-(pyrrolidin-1-yl)-7-(pyrrolidin-1-ylsulfonyl)-[1,2,4]triazolo[4,3-***a***]quinoxalin-1-amine (13a)

Deep-Brown powder (1,4-dioxane); 73% yield; m.p. 268 − 266 ºC; **IR (KBr)**: ν_max_ = 3345 (NH), 3028 (CH_**ar**_), 2966, 2874 (CH_**alip**_), 1597 (C = N), 1335, 1199 (SO_2_) cm^-1^;^1^**H NMR (δ**,** ppm)** = 1.69 (8 H, s, (CH_2_)_4_-pyrrolidine), 3.17 (8 H, s, N(CH_2_)_4_-pyrrolidine), 4.23 (2 H, s, CH_2_-CH-allyl), 5.28–5.37 (2 H, m,CH_2_ = CH-allyl), 5.88(1 H, s, CH_2_ = CH-allyl), 6.10 (1 H, s, NH, D_2_O exchangeable), 8.06–8.39 (2 H, m, H_8+_H_7_. quinox), 8.70 (1 H, s, H_5_. quinox*).*^13^**C NMR (δ**,** ppm)** = 25.85 (4CH_2_.pyrrolidine), 46.47 (CH_2_-CH-allyl), 49.02 (4 N-CH_2_.pyrrolidine), 101.03 (CH_2_ = CH-allyl), 117.45 (CH_2_ = CH-allyl), 122.62, 127.10, 128.04, 128.44, 132.12, 134.17, 135.92, 146.48 (Ar.Cs), 157.62 (N = C-N); **Anal. Calcd** for **C**_**20**_**H**_**25**_**N**_**7**_**O**_**2**_**S** (427.53): C, 56.19; H, 5.89; N, 22.93; Found: C, 56.30; H, 5.99; N, 22.75.

#### ***N***-Phenyl-4-(pyrrolidin-1-yl)-7-(pyrrolidin-1-ylsulfonyl)-[1,2,4]triazolo[4,3-***a***]quinoxalin-1-amine (13b)

Brown powder (1,4-dioxane); 75% yield; m.p. 290–292 ºC; **IR (KBr)**: ν_max_ = 3346 (NH), 3056 (CH_**ar**_), 2925, 2869 (CH_**alip**_), 1597 (C = N), 1318, 1198 (SO_2_) cm^-1^;^1^**H NMR (*δ***, **ppm)** = 1.67 (8 H, s, (CH_2_)_4_-pyrrolidine), 3.20 (8 H, s, N(CH_2_)_4_-pyrrolidine), 6.93 (1 H, t, *J* = 7.6 Hz, ph-H), 7.11 (2 H, t, *J* = 7.2 Hz, ph-H), 7.31–7.36 (2 H, m, ph-H), 7.44 (1 H, d, *J* = 7.6 Hz, H_8_. quinox), 7.50 (1 H, d, *J* = 8.0 Hz, H_7_. quinox), 8.62 (1 H, s, H_5_. quinox), 9.92 (1 H, s, NH, D_2_O exchangeable*).*^13^**C NMR (*δ***, **ppm)** = 25.25 ((CH_2_)_2_-pyrrolidine), 48.39 (N(CH_2_)_2_-pyrrolidine), 115.44, 117.25, 118.58, 124.01, 124.82, 126.24, 128.27, 128.88, 129.43, 130.39, 137.64, 139.96, 141.68, 149.33 (Ar.Cs), 156.14 (N = C-N); **Anal. Calcd** for **C**_**23**_**H**_**25**_**N**_**7**_**O**_**2**_**S** (463.56): C, 59.59; H, 5.44; N, 21.15; Found: C, 59.39; H, 5.67; N, 21.03.

### Biological evaluation

#### In-vitro anti-diabetic activity

This assay was conducted by calculating the percentage (%) inhibition of α-amylase and α-glucosidase enzymes at a concentration of 100 µM of the tested quinoxaline sulfonamides, by the methodologies established by Wickramaratne and the Pistia-Brueggeman method, as previously described^70,71^. Acarbose was employed as the standard drug. The supplementary information (SI) file provides all procedural steps and detailed information. Additionally, the values of the median inhibitory concentrations for the most active derivative **10a** and acarbose as positive control were calculated from the curve plotted between the percentage of inhibition and a series of concentrations (2, 4, 6.25, 12.5, and 25 µg/mL). (all procedural steps and details are provided in the supplementary information file).

#### In-vitro anti-Alzheimer activity

This assay was conducted by calculating the percent inhibition (%) of the acetylcholinesterase (AChE) enzyme at a concentration of 100 µg/mL of the tested quinoxaline derivatives, following Ellman’s method^72^. Consequently, the active human AChE enzyme hydrolyzes the colorimetric quinoxaline sulfonamides, resulting in the generation of yellow chromophores that are detectable at 412 nm through absorbance measurement. Donepezil was used as the standard drug (all procedural steps and details are provided in the supplementary information file).

### Molecular docking simulation

The molecular docking simulation was performed for the most active derivatives triazolo[4,3-*a*]quinoxalin-1-amine derivative **10a** inside the active site of α-amylase (PDB: 2QV4) and α-glucosidase (PDB: 3W37) as anti-diabetic targets. On the other hand, the most active 1-methyl-[1,2,4]triazolo[4,3-*a*]quinoxaline derivative **11b** on the acetylcholinesterase based on the inhibitory percentage as described on biological evaluation results was docked inside the active site of acetylcholinesterase (ACHE) (PDB: 4EY7). The docking simulation was carried out using Molecular operating Environmental (MOE) 10.2009 ^73–75^. All the crystallography protein structures were downloaded from protein data bank (https://www.rcsb.org/) as pdb file and containing acarbose or donepezil as co-crystallized ligand. The structure of the most active triazolo-quinoxaline 10a and **11b** was built using chembiodraw 14 as described previously^5,76^. The active site of α-amylase (PDB: 2QV4) and α-glucosidase (PDB: 3W37) was generated as described previously and according to standard protocol^9,10^. Initially, the validation process for α-amylase (PDB: 2QV4) exhibited that the acarbose as co-crystallized ligand showed binding affinity S =- 16.33 kcal/mol with RMSD = 1.36 Å through one hydrogen bond backbone acceptor with Thr163, one hydrogen bond sidechain donor with His201, and four hydrogen bond sidechain acceptors with residues (2 x Asp300, His299, and Glu233). On the other hand, for the α-glucosidase (PDB: 3W37) the acarbose as positive control and co-crystallized ligand displayed binding affinity S =- 16.82 kcal/mol with RMSD = 2.357 Å in the validation process. The acarbose bounded to the pocket through seven hydrogen bond sidechain acceptors with amino acids (Asp232, Asp568, His625, and Asp357) and one hydrogen bond sidechain donor with Asp552. For the validation process of acetylcholinesterase (ACHE) (PDB: 4EY7)^14^ the donepezil as co-crystallized ligand exhibited binding affinity S = -11.027 kcal/mol with RMSD = 0.8064 Å through two types of interactions as arene-arene interactions with residues (Trp286 and Trp86) and two arene-cation interaction with residues (Tyr337 and Tyr341).

## Electronic supplementary material

Below is the link to the electronic supplementary material.

Supplementary Material 1

## Data Availability

Data availabilityThe datasets used and/or analyzed during the current study are available from the corresponding author on reasonable request.

## Abbreviations

ROS: Reactive oxygen species
RNS: Reactive nitrogen species
FDA: Food and drug administration
AD: Alzheimer’s disease
AChE: Acetylcholinesterase
BChE: Butyrylcholinesterase
ACh: Acetylcholine
SAR: Structure-activity relationship
IP: Inhibitory percentage
PDB: Protein data bank

## References

[1] Forbes, J. M. & Cooper, M. E. Mechanisms of diabetic complications. *Physiol. Rev.* **93**, 137–188 (2013). 23303908 10.1152/physrev.00045.2011

[2] Girach, A., Manner, D. & Porta, M. Diabetic microvascular complications: can patients at risk be identified? A review. *Int. J. Clin. Pract.* **60**, 1471–1483 (2006). 17073842 10.1111/j.1742-1241.2006.01175.x

[3] Janghorbani, M., Van Dam, R. M., Willett, W. C. & Hu, F. B. Systematic review of type 1 and type 2 diabetes mellitus and risk of fracture. *Am. J. Epidemiol.* **166**, 495–505 (2007). 17575306 10.1093/aje/kwm106

[4] Larsson, S. C., Orsini, N. & Wolk, A. Diabetes mellitus and risk of colorectal cancer: a meta-analysis. *J. Natl. Cancer Inst.* **97**, 1679–1687 (2005). 16288121 10.1093/jnci/dji375

[5] Thabet, H. K. et al. Innovation of 6-sulfonamide-2H-chromene derivatives as antidiabetic agents targeting α-amylase, α-glycosidase, and PPAR-γ inhibitors with in Silico molecular Docking simulation. *RSC Adv.* **14**, 15691–15705 (2024). 38746843 10.1039/d4ra02143f PMC11091863

[6] Ighodaro, O. M. Molecular pathways associated with oxidative stress in diabetes mellitus. *Biomed. Pharmacother*. **108**, 656–662 (2018). 30245465 10.1016/j.biopha.2018.09.058

[7] Giacco, F. & Brownlee, M. Oxidative stress and diabetic complications. *Circ. Res.* **107**, 1058–1070 (2010). 21030723 10.1161/CIRCRESAHA.110.223545 PMC2996922

[8] Gohar, N. A. et al. Fluorinated indeno-quinoxaline bearing thiazole moieties as hypoglycaemic agents targeting α-amylase, and α-glucosidase: synthesis, molecular docking, and ADMET studies. *J. Enzyme Inhib. Med. Chem.* **39**, 2367128 (2024). 38913598 10.1080/14756366.2024.2367128 PMC467095

[9] Khamees Thabet, H. et al. Discovery of new anti-diabetic potential agents based on Paracetamol incorporating sulfa-drugs: design, synthesis, α-amylase, and α-glucosidase inhibitors with molecular Docking simulation. *Eur. J. Med. Chem.* **275**, 116589 (2024). 38878516 10.1016/j.ejmech.2024.116589

[10] Khamees Thabet, H. et al. Unveiling anti-diabetic potential of new thiazole-sulfonamide derivatives: design, synthesis, in vitro bio-evaluation targeting DPP-4, α-glucosidase, and α-amylase with in-silico ADMET and Docking simulation. *Bioorg. Chem.* **151**, 107671 (2024). 39067419 10.1016/j.bioorg.2024.107671

[11] Hollander, P. Safety profile of acarbose, an α-glucosidase inhibitor. *Drugs* **44**, 47–53 (1992). 1280577 10.2165/00003495-199200443-00007

[12] Scott, L. J. & Spencer, C. M. Miglitol: a review of its therapeutic potential in type 2 diabetes mellitus. *Drugs* **59**, 521–549 (2000). 10776834 10.2165/00003495-200059030-00012

[13] Hassan, A. S., Morsy, N. M., Aboulthana, W. M. & Ragab, A. In vitro enzymatic evaluation of some pyrazolo[1,5-a]pyrimidine derivatives: design, synthesis, antioxidant, anti-diabetic, anti-Alzheimer, and anti-arthritic activities with molecular modeling simulation. *Drug Dev. Res.* **84**, 3–24 (2023). 36380556 10.1002/ddr.22008

[14] Ragab, A. et al. Explore new Quinoxaline pharmacophore tethered sulfonamide fragments as in vitro $α$-glucosidase, $α$‐amylase, and acetylcholinesterase inhibitors with ADMET and molecular modeling simulation. *Drug Dev. Res.* **85**, e22216 (2024). 38831547 10.1002/ddr.22216

[15] Greig, N. H., Lahiri, D. K. & Sambamurti, K. Butyrylcholinesterase: an important new target in Alzheimer’s disease therapy. *Int. Psychogeriatr.* **14**, 77–91 (2002). 12636181 10.1017/s1041610203008676

[16] Kandimalla, R., Thirumala, V. & Reddy, P. H. Is Alzheimer’s disease a type 3 diabetes?? A critical appraisal. *Biochim. Biophys. Acta - Mol. Basis Dis.* **1863**, 1078–1089 (2017). 27567931 10.1016/j.bbadis.2016.08.018 PMC5344773

[17] Yang, Y. & Song, W. Molecular links between Alzheimer’s disease and diabetes mellitus. *Neuroscience* **250**, 140–150 (2013). 23867771 10.1016/j.neuroscience.2013.07.009

[18] Escobar-Chavez, J. J. et al. The use of iontophoresis in the administration of nicotine and new non-nicotine drugs through the skin for smoking cessation. *Curr. Drug Discov Technol.* **6**, 171–185 (2009). 19496753 10.2174/157016309789054924

[19] McLAUGHLIN, M. A. & CHIOU, G. C. Y. A synopsis of recent developments in antiglaucoma drugs. *J. Ocul Pharmacol. Ther.* **1**, 101–121 (1985). 10.1089/jop.1985.1.101 3916848

[20] HUGO, W. B. & STRETTON, R. G. Action of Quinacillin on Staphylococcus aureus. *Nature* **202**, 1217 (1964). 14217514 10.1038/2021217a0

[21] Shintre, S. A. et al. Synthesis, in vitro antimicrobial, antioxidant, and antidiabetic activities of thiazolidine–quinoxaline derivatives with amino acid side chains. *Med. Chem. Res.* **26**, 2141–2151 (2017).

[22] Hajri, M. et al. Synthesis and evaluation of in vitro antiproliferative activity of new Ethyl 3-(arylethynyl) quinoxaline-2-carboxylate and pyrido [4, 3-b] quinoxalin-1 (2H)-one derivatives. *Eur. J. Med. Chem.* **124**, 959–966 (2016). 27770736 10.1016/j.ejmech.2016.10.025

[23] Achutha, L., Parameshwar, R., Reddy, B. M. & Babu, V. H. Microwave-assisted synthesis of some quinoxaline-incorporated Schiff bases and their biological evaluation. *J. Chem.* (2013).

[24] Quiliano, M. et al. New hydrazine and Hydrazide Quinoxaline 1, 4-di-N-oxide derivatives: in Silico ADMET, antiplasmodial and antileishmanial activity. *Bioorg. Med. Chem. Lett.* **27**, 1820–1825 (2017). 28291694 10.1016/j.bmcl.2017.02.049

[25] Patel, H. M. et al. Quinoxaline-PABA bipartite hybrid derivatization approach: design and search for antimicrobial agents. *J. Mol. Struct.* **1184**, 562–568 (2019).

[26] Ragab, A. et al. New prospective insecticidal agents based on Thiazolo [4, 5-b] Quinoxaline derivatives against cotton leafworm Spodoptera litura (Fabricius): design, synthesis, toxicological, morphology, histological, and biomedical studies. *Pestic Biochem. Physiol* 105943 (2024). 10.1016/j.pestbp.2024.105943 38879303

[27] Elsisi, D. M. et al. Synthesis and modification of novel thiazole-fused quinoxalines as new insecticidal agents against the cotton leafworm Spodoptera Litura: design, characterization, in vivo bio-evaluation, toxicological effectiveness, and study their mode of action. *RSC Adv.* **15**, 1391–1406 (2025). 39822566 10.1039/d4ra08096c PMC11736854

[28] Ammar, Y. A. et al. Design, green synthesis, and quorum sensing quenching potential of novel 2-oxo-pyridines containing a Thiophene/furan scaffold and targeting a Las R gene on P. aeruginosa. *RSC Adv.* **13**, 27363–27384 (2023). 37711372 10.1039/d3ra04230h PMC10498153

[29] Suwanhom, P. et al. Synthesis, Biological evaluation, and in silico studies of new acetylcholinesterase inhibitors based on quinoxaline scaffold. *Molecules* 26 at (2021). 10.3390/molecules26164895 10.3390/molecules26164895 PMC8400540 34443482

[30] Sharma, P., Gupta, G. D. & Asati, V. Design, synthesis and antidiabetic study of Triazole clubbed Indole derivatives as $α$-glucosidase inhibitors. *Bioorg. Chem.* **139**, 106750 (2023). 37499530 10.1016/j.bioorg.2023.106750

[31] Collin, X., Sauleau, A. & Coulon, J. 1, 2, 4-Triazolo mercapto and aminonitriles as potent antifungal agents. *Bioorg. Med. Chem. Lett.* **13**, 2601–2605 (2003). 12852975 10.1016/s0960-894x(03)00378-0

[32] Ayman, R., Abusaif, M. S., Radwan, A. M., Elmetwally, A. M. & Ragab, A. Development of novel pyrazole, imidazo[1,2-b]pyrazole, and pyrazolo[1,5-a]pyrimidine derivatives as a new class of COX-2 inhibitors with Immunomodulatory potential. *Eur. J. Med. Chem.* **249**, 115138 (2023). 36696764 10.1016/j.ejmech.2023.115138

[33] Ragab, A., Fouad, S. A., Ammar, Y. A., Aboul-Magd, D. S. & Abusaif, M. S. Antibiofilm and anti-quorum-sensing activities of novel pyrazole and pyrazolo[1,5-a]pyrimidine derivatives as carbonic anhydrase I and II inhibitors: design, synthesis, radiosterilization, and molecular docking studies. *Antibiotics* **12** 128 (2023). 10.3390/antibiotics12010128 10.3390/antibiotics12010128 PMC9854762 36671329

[34] Asogwa, F. C. et al. Synthesis, characterization, DFT studies and molecular docking investigation of 2-oxo-ethyl piperidine pentanamide-derived sulfonamides as anti-diabetic agents. *Results Chem.* **4**, 100672 (2022).

[35] Kucukguzel, I., Tatar, E., Kucukguzel, S. G., Rollas, S. & De Clercq, E. Synthesis of some novel thiourea derivatives obtained from 5-[(4-aminophenoxy) methyl]-4-alkyl/aryl-2, 4-dihydro-3H-1, 2, 4-triazole-3-thiones and evaluation as antiviral/anti-HIV and anti-tuberculosis agents. *Eur. J. Med. Chem.* **43**, 381–392 (2008). 17583388 10.1016/j.ejmech.2007.04.010

[36] Palaska, E., Şahin, G., Kelicen, P., Durlu, N. T. & Altinok, G. Synthesis and anti-inflammatory activity of 1-acylthiosemicarbazides, 1, 3, 4-oxadiazoles, 1, 3, 4-thiadiazoles and 1, 2, 4-triazole-3-thiones. *Farm* **57**, 101–107 (2002). 10.1016/s0014-827x(01)01176-4 11902651

[37] Mavrova, A. T., Wesselinova, D., Tsenov, Y. A. & Denkova, P. Synthesis, cytotoxicity and effects of some 1, 2, 4-triazole and 1, 3, 4-thiadiazole derivatives on immunocompetent cells. *Eur. J. Med. Chem.* **44**, 63–69 (2009). 18439727 10.1016/j.ejmech.2008.03.006

[38] Haber, J. Present status and perspectives on antimycotics with systemic effects. *Cas Lek Cesk.* **140**, 596–604 (2001). 11715729

[39] Mahmood, S., Khan, S. G., Rasul, A., Christensen, J. B. & Abourehab, M. A. S. Ultrasound Assisted Synthesis and In Silico Modelling of 1,2,4-Triazole Coupled Acetamide Derivatives of 2-(4-Isobutyl phenyl)propanoic acid as Potential Anticancer Agents. *Molecules* **27** (2022). 10.3390/molecules27227984 10.3390/molecules27227984 PMC9698963 36432091

[40] Nguyen, G. V. et al. Novel sulfonamide-functionalized arylidene indolones as potent $α$-glucosidase inhibitors: synthesis, characterization, and in vitro and in Silico studies. *Mendeleev Commun.* **33**, 543–545 (2023).

[41] Taha, M. et al. Synthesis, characterization, biological evaluation, and kinetic study of Indole base sulfonamide derivatives as acetylcholinesterase inhibitors in search of potent anti-Alzheimer agent. *J. King Saud Univ.* **33**, 101401 (2021).

[42] Mirian, M. et al. Synthesis and cytotoxic evaluation of some novel Sulfonamidederivativesagainst a few human cancer cells. *Iran. J. Pharm. Res. IJPR*. **10**, 741 (2011). 24250409 PMC3813052

[43] Link, J. T. et al. Optimization and metabolic stabilization of a class of nonsteroidal glucocorticoid modulators. *Bioorg. Med. Chem. Lett.* **14**, 4169–4172 (2004). 15261264 10.1016/j.bmcl.2004.06.023

[44] El-Kalyoubi, S. A. et al. One-pot synthesis and molecular modeling studies of new bioactive spiro-oxindoles based on uracil derivatives as SARS-CoV-2 inhibitors targeting RNA polymerase and spike glycoprotein. *Pharmaceuticals*** 15**, (2022). 10.3390/ph15030376 PMC8954694 35337173

[45] Jeelan Basha, N., Basavarajaiah, S. M. & Shyamsunder, K. Therapeutic potential of pyrrole and pyrrolidine analogs: an update. *Mol. Divers.* **26**, 2915–2937 (2022). 35079946 10.1007/s11030-022-10387-8 PMC8788913

[46] Li Petri, G. et al. Pyrrolidine in drug discovery: A versatile scaffold for novel biologically active compounds. *Top. Curr. Chem.* **379**, 34 (2021). 10.1007/s41061-021-00347-5 PMC8352847 34373963

[47] Liao, A. et al. Pyrrole and pyrrolidine analogs: the promising scaffold in discovery of pesticides. *Chin. Chem. Lett.* **36**, 110094 (2025).

[48] Helal, M. H., Abbas, S. Y., Salem, M. A., Farag, A. A. & Ammar, Y. A. Synthesis and characterization of new types of 2-(6-methoxy-2-naphthyl)propionamide derivatives as potential antibacterial and antifungal agents. *Med. Chem. Res.* **22**, 5598–5609 (2013).

[49] Khattab, E. S. A. E. H., Ragab, A., Abol-Ftouh, M. A. & Elhenawy, A. A. Therapeutic strategies for Covid-19 based on molecular docking and dynamic studies to the ACE-2 receptors, Furin, and viral Spike proteins. *J. Biomol. Struct. Dyn.* **40**, 13291–13309 (2022). 34647855 10.1080/07391102.2021.1989036 PMC8544674

[50] Fayed, E. A., Ragab, A., Ezz Eldin, R. R., Bayoumi, A. H. & Ammar, Y. A. In vivo screening and toxicity studies of Indolinone incorporated thiosemicarbazone, thiazole and Piperidinosulfonyl moieties as anticonvulsant agents. *Bioorg. Chem.* **105300**10.1016/j.bioorg.2021.105300 (2021). 10.1016/j.bioorg.2021.105300 34525393

[51] Ali, O. A. A., Ragab, A., Ammar, Y. A. & Abusaif, M. S. Discovery of new thiazolidin-4-one and thiazole nucleus incorporation Sulfaguanidine scaffold as new class of antimicrobial agents: design, synthesis, in Silico ADMET, and Docking simulation. *J. Mol. Struct.* **1334**, 141879 (2025).

[52] Raslan, R. R. et al. Evaluation of the anti-proliferative activity of 2-oxo-pyridine and 1′H-spiro-pyridine derivatives as a new class of EGFRWt and VEGFR-2 inhibitors with apoptotic inducers. *RSC Adv.* **13**, 10440–10458 (2023). 37020892 10.1039/d3ra00887h PMC10069231

[53] Ragab, A. Recent advances in the synthesis, reaction, and bio-evaluation potential of purines as precursor pharmacophores in chemical reactions: a review. *RSC Adv.* **15**, 3607–3645 (2025). 39906628 10.1039/d4ra08271k PMC11793083

[54] Wassel, M. S., Gamal Eldin, M. M., Ragab, W., Elhag Ali, A. A. M., Ammar, A. & G. & Antiviral activity of Adamantane-Pyrazole derivatives against foot and mouth disease virus infection in vivo and in vitro with molecular Docking study. *J. Appl. Vet. Sci.* **5**, 37–46 (2020).

[55] Abdel-Baky, Y. M. et al. Developing a new multi-featured chitosan-quinoline schiff base with potent antibacterial, antioxidant, and antidiabetic activities: design and molecular modeling simulation. *Sci. Rep.* **13**, 22792 (2023). 38123716 10.1038/s41598-023-50130-3 PMC10733428

[56] Ismail, M. M. F. et al. Novel quinoxaline-3-propanamides as VGFR-2 inhibitors and apoptosis inducers. *RSC Adv.* **13**, 31908–31924 (2023). 37915441 10.1039/d3ra05066a PMC10616755

[57] Abusaif, M. S. et al. Effect of different acceptors on N-hexyl carbazole moiety for dye-sensitized solar cells: design, characterization, molecular structure, and DSSC fabrications. *J. Iran. Chem. Soc.* **18**, 949–960 (2021).

[58] El-Gaby, M. S. A., Ammar, Y. A., Ismail, M. A., Ragab, A. & Abusaif, M. S. Synthesis, characterization, and biological target prediction of novel 1,3-dithiolo[4,5- B ]quinoxaline and thiazolo[4,5- B ]quinoxaline derivatives. *Heterocycl Commun* **29**, (2023).

[59] Abusaif, M. S. et al. Novel Water-soluble quinoxaline-2,3-dione-6-sulfohydrazide derivatives as efficient acid corrosion inhibitors: design, characterization, experimental, and theoretical studies. *J. Taiwan. Inst. Chem. Eng.* **153**, 105207 (2023).

[60] Ragab, A. et al. Development of new spiro[1,3]dithiine-4,11′-indeno[1,2-b]quinoxaline derivatives as S. aureus sortase A inhibitors and radiosterilization with molecular modeling simulation. *Bioorg. Chem.* **131**, 106307 (2023). 36481380 10.1016/j.bioorg.2022.106307

[61] Ismail, M. A., Abusaif, M. S., El-Gaby, M. S. A., Ammar, Y. A. & Ragab, A. A new class of anti-proliferative activity and apoptotic inducer with molecular Docking studies for a novel of 1,3-dithiolo[4,5- B ]quinoxaline derivatives hybrid with a sulfonamide moiety. *RSC Adv.* **13**, 12589–12608 (2023). 37101951 10.1039/d3ra01635h PMC10123497

[62] Ali, A. A. et al. Design, characterization, theoretical studies, and dyeing properties of new novel Diazo salicylaldehyde schiff base catalyzed with ceric (IV) ammonium nitrate (CAN) as an eco-friendly catalyst. *Pigment Resin Technol.*10.1108/PRT-12-2022-0141 (2023).

[63] Abd-elmaksoud, G. A. et al. Characterization, DFT computational study, and evaluation the performance of some new N-Amino pyridinone schiff base catalyzed with Ceric(IV) ammonium nitrate (CAN) as corrosion inhibitors in some petroleum applications. *Arab. J. Sci. Eng.*10.1007/s13369-023-08073-4 (2023).

[64] Ragab, A., Ayman, R., Salem, M. A., Ammar, Y. A. & Abusaif, M. S. Unveiling a novel pyrazolopyrimidine scaffold as a dual COX-2/5-LOX inhibitor with Immunomodulatory potential: design, synthesis, target prediction, anti-inflammatory activity, and ADME-T with Docking simulation. *Eur. J. Med. Chem.* **290**, 117499 (2025). 40101450 10.1016/j.ejmech.2025.117499

[65] Mohamed, H. A. et al. Discovery a novel of thiazolo[3,2-a]pyridine and pyrazolo[3,4-d]thiazole derivatives as DNA gyrase inhibitors; design, synthesis, antimicrobial activity, and some in-silico ADMET with molecular Docking study. *J. Mol. Struct.* **1287**, 135671 (2023).

[66] Obafemi, C. A. & Akinpelu, D. A. Synthesis and antimicrobial activity of some 2(1H)-quinoxalinone-6-sulfonyl derivatives. *Phosphorus Sulfur Silicon Relat. Elem.* **180**, 1795–1807 (2005).

[67] El-Din, N. S. Synthesis of some sulfonamide derivatives with potential antibacterial activity. *Chem Heterocycl. Compd* 523–528 (2000).

[68] Ragab, A. et al. Design, synthesis of new novel quinoxalin-2(1H)-one derivatives incorporating hydrazone, hydrazine, and pyrazole moieties as antimicrobial potential with in-silico ADME and molecular Docking simulation. *Arab. J. Chem.* **15**, 103497 (2022).

[69] Ammar, Y. A. et al. Carboxamide appended Quinoline moieties as potential anti-proliferative agents, apoptotic inducers and Pim-1 kinase inhibitors. *Med. Chem. Res.* **30**, 1649–1668 (2021).

[70] Pistia-Brueggeman, G. & Hollingsworth, R. I. A Preparation and screening strategy for glycosidase inhibitors. *Tetrahedron* **57**, 8773–8778 (2001).

[71] Wickramaratne, M. N., Punchihewa, J. C. & Wickramaratne, D. B. M. In-vitro alpha amylase inhibitory activity of the leaf extracts of adenanthera pavonina. *BMC Complement. Altern. Med.* **16**, 466 (2016). 27846876 10.1186/s12906-016-1452-y PMC5109804

[72] Ellman, G. L., Courtney, K. D., Andres, V. & Featherstone, R. M. A new and rapid colorimetric determination of acetylcholinesterase activity. *Biochem. Pharmacol.* **7**, 88–95 (1961). 13726518 10.1016/0006-2952(61)90145-9

[73] Rizk, H. F., El-Borai, M. A., Ragab, A., Ibrahim, S. A. & Sadek, M. E. A novel of Azo-Thiazole moiety alternative for Benzidine-Based pigments: design, synthesis, characterization, biological evaluation, and molecular Docking study. *Polycycl. Aromat. Compd.* **43**, 500–522 (2023).

[74] Ammar, Y. A. et al. Development and radiosterilization of new hydrazono-quinoline hybrids as DNA gyrase and topoisomerase IV inhibitors: antimicrobial and hemolytic activities against uropathogenic isolates with molecular Docking study. *Chem. Biol. Drug Des.* **101**, 245–270 (2023). 36305722 10.1111/cbdd.14154

[75] Abdelgalil, M. M., Ammar, Y. A., Ali, E., Ali, G. A. M., Ragab, A. & A. K. & A novel of Quinoxaline derivatives tagged with pyrrolidinyl scaffold as a new class of antimicrobial agents: design, synthesis, antimicrobial activity, and molecular Docking simulation. *J. Mol. Struct.* **1274**, 134443 (2023).

[76] Thabet, H. K. et al. Discovery of novel 6-(piperidin-1-ylsulfonyl)-2H-chromenes targeting $α$-glucosidase, $α$-amylase, and PPAR-$γ$: design, synthesis, virtual screening, and anti-diabetic activity for type 2 diabetes mellitus. *Comput. Biol. Chem.* **111**, 108097 (2024). 38772048 10.1016/j.compbiolchem.2024.108097

