# Supplementary material for: Exploring novel of 1,2,4-triazolo[4,3-a]quinoxaline sulfonamide regioisomers as anti-diabetic and anti-Alzheimer agents with in-silico molecular docking simulation
Source: Sci Rep. 2025 Jun 3;15:19409. doi: 10.1038/s41598-025-03139-9 (PMC12134304; doi:10.1038/s41598-025-03139-9)
Supplement: Supplementary file 1 — Supplementary Material 1 [file 41598_2025_3139_MOESM1_ESM.pdf]

Exploring novel of 1,2,4-triazolo[4,3-*a*]quinoxaline sulfonamide regioisomers as anti-diabetic and anti-Alzheimer agents with *in-silico* molecular docking simulation

Moustafa S. Abusaif [a], Ahmed M. Sh El-Sharief [a], Yehia A. Mohamed [a], Yousry A. Ammar [a], Mostafa A. Ismail [b], Wael M. Aboulthana [c], Mohamed S. A. El-Gaby [a], Ahmed Ragab [d,a,*]

[a] Department of Chemistry, Faculty of Science (boys), Al-Azhar University, 11884 Nasr City, Cairo-Egypt
[b] Chemistry Department, Faculty of Science, Al-Azhar University, Assiut, 71524, Egypt

[c] Biochemistry Department, Biotechnology Research Institute, National Research Centre, 33 El Buhouth St., Dokki 12622, Cairo, Egypt
[d] Chemistry Department, Faculty of Science, Galala University, Galala City, Suez, 43511, Egypt.

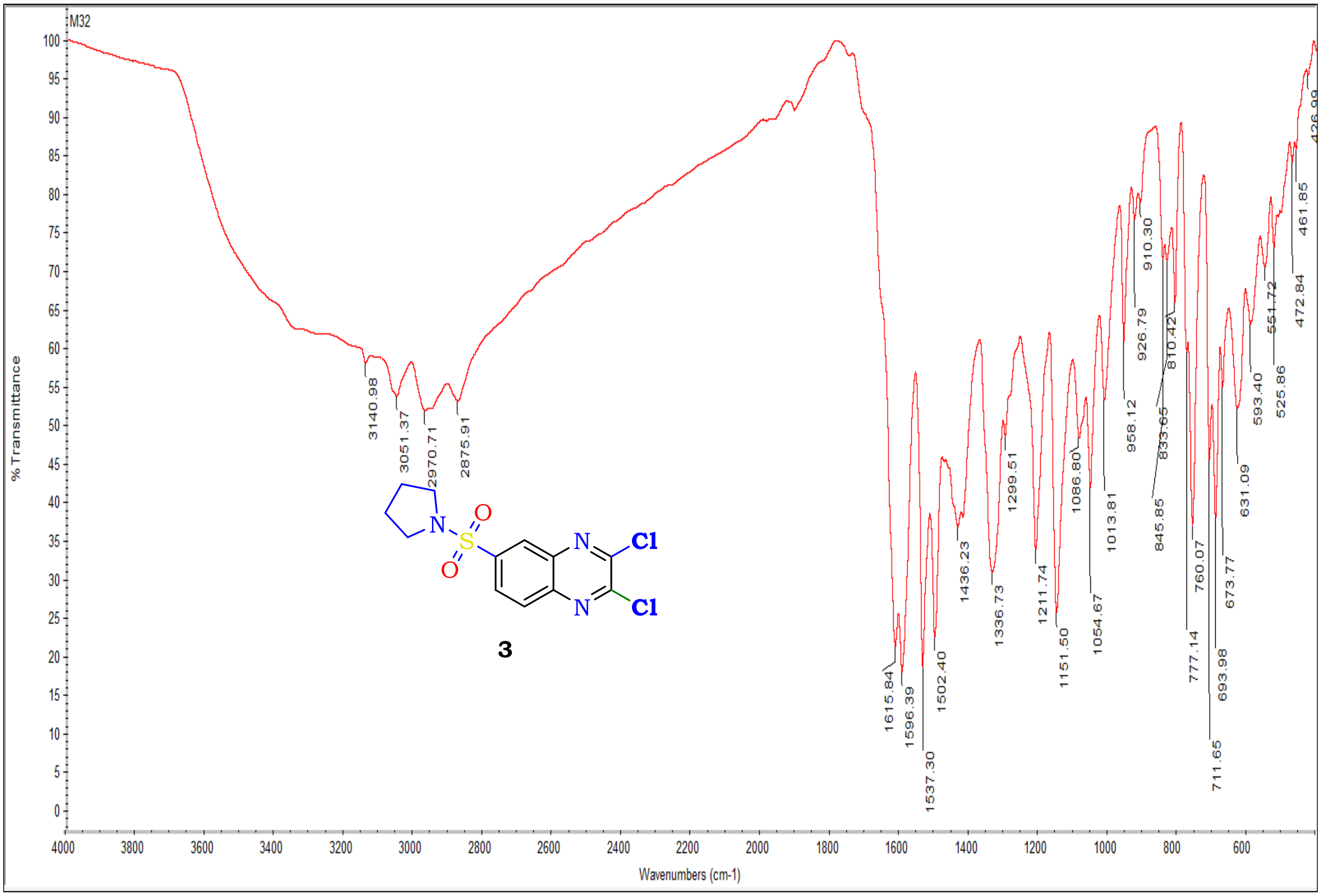

Figure SI1: IR spectrum of compound 3

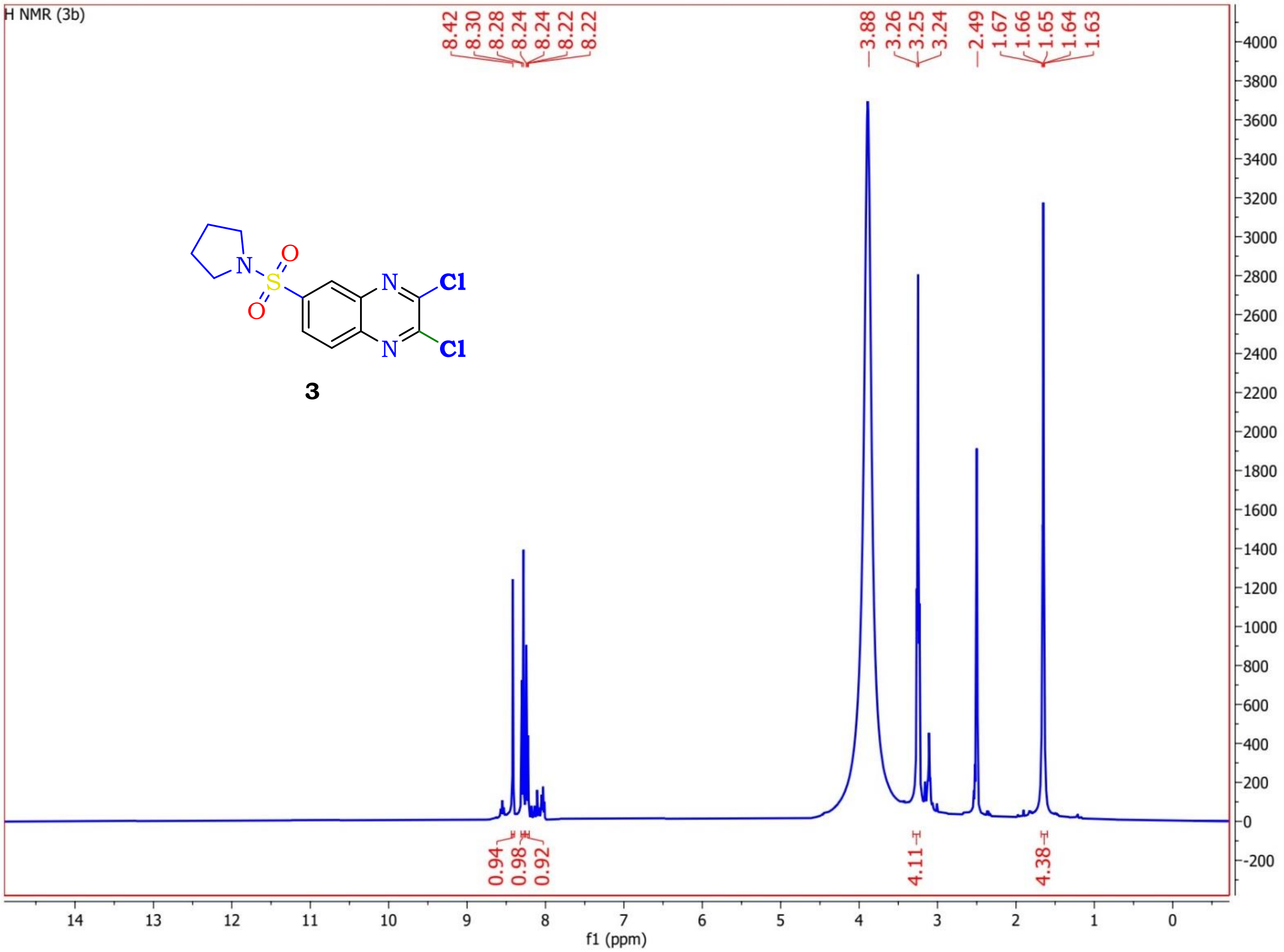

Figure SI2: ^{1}H NMR spectrum of compound **3**

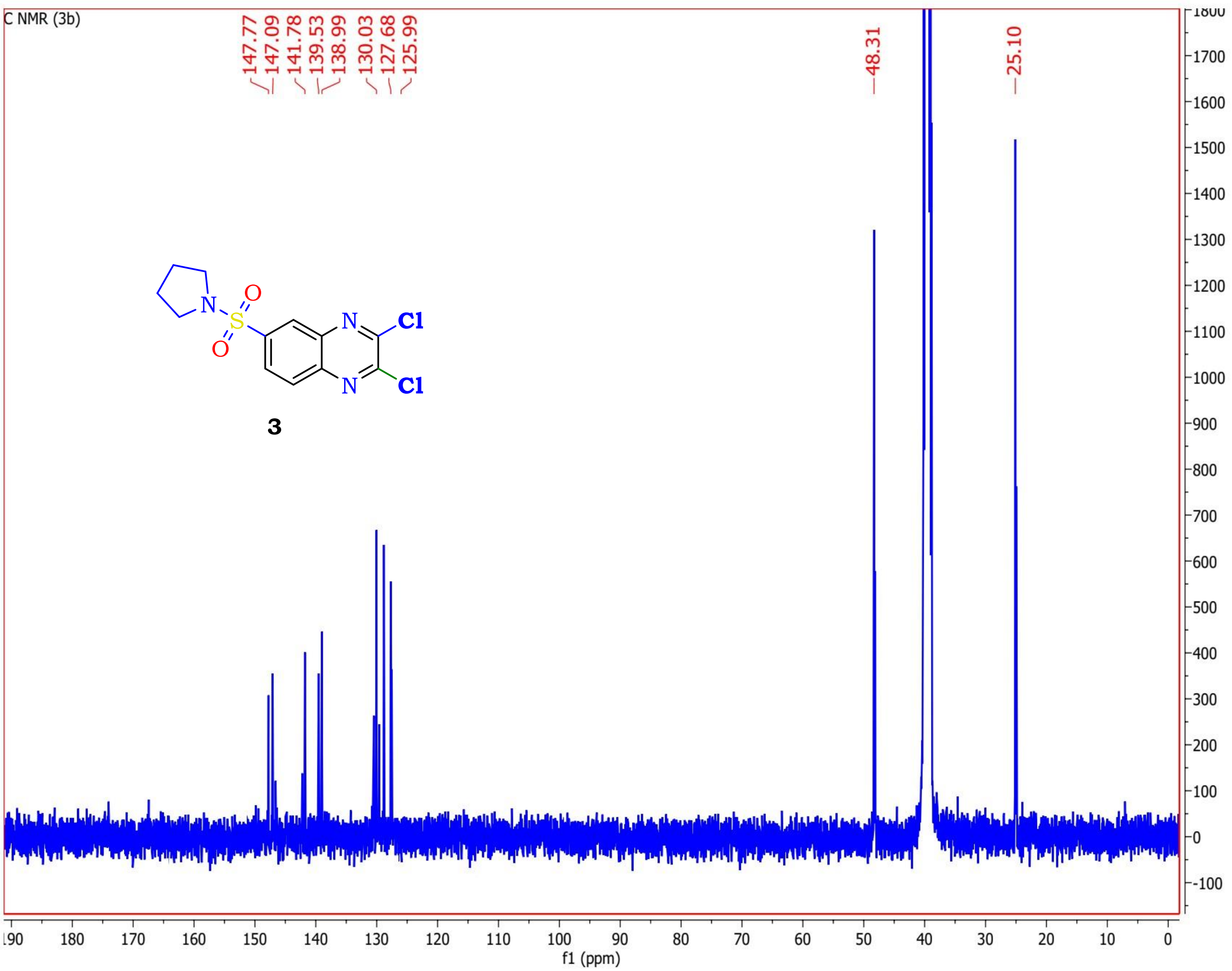

Figure SI3: ^{13}C NMR spectrum of compound **3**

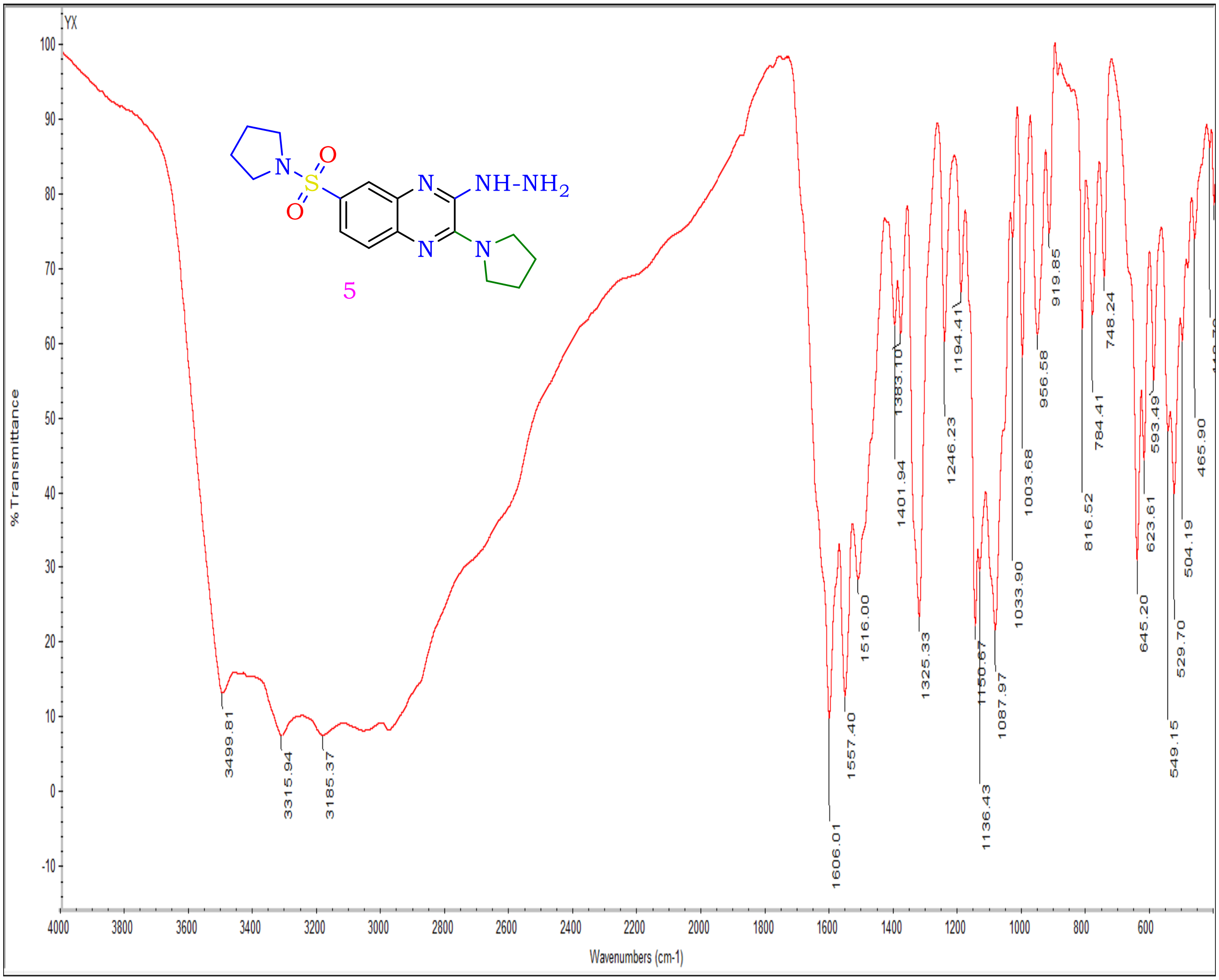

Figure SI4: IR spectrum of compound **4**

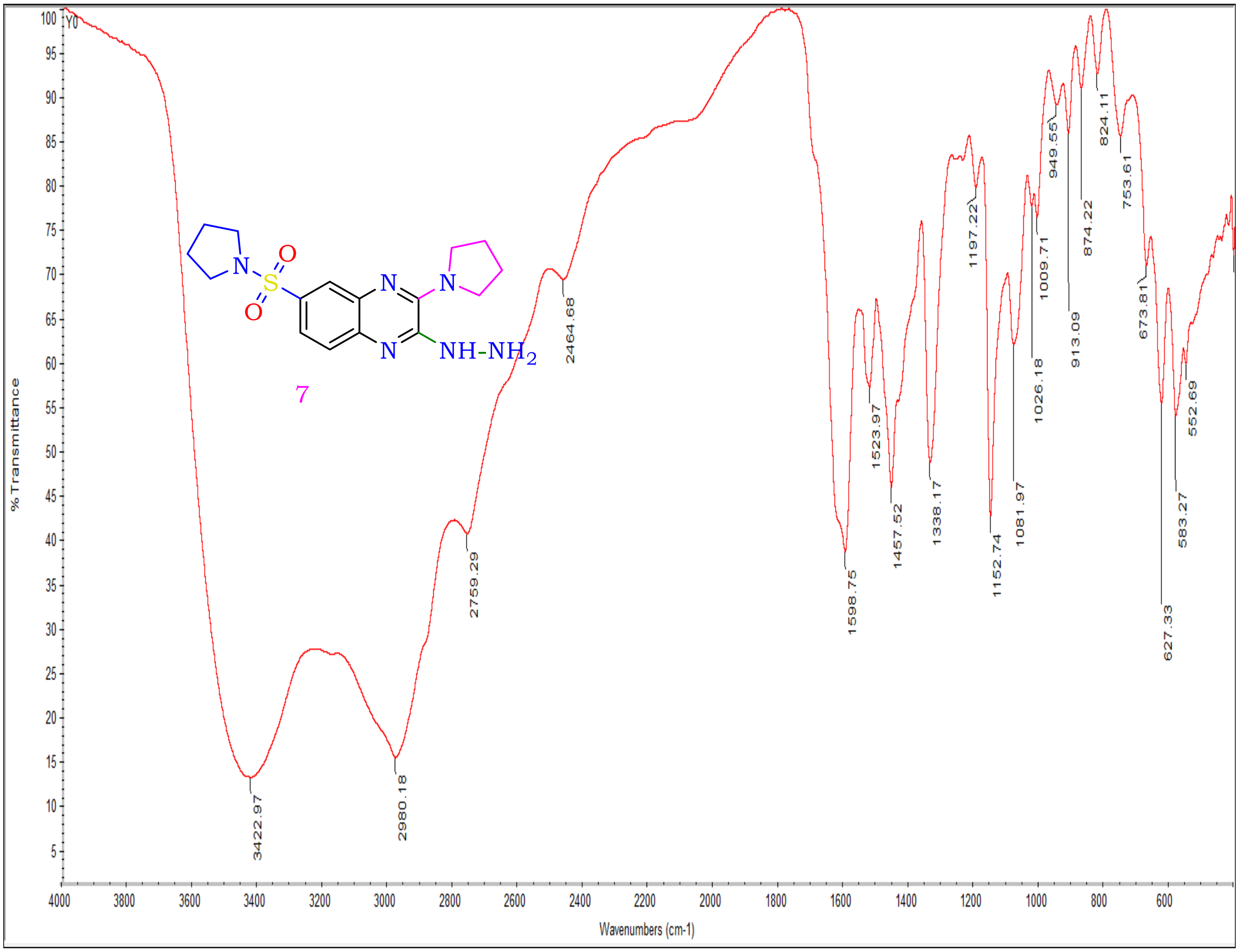

Figure SI5: IR spectrum of compound **7**

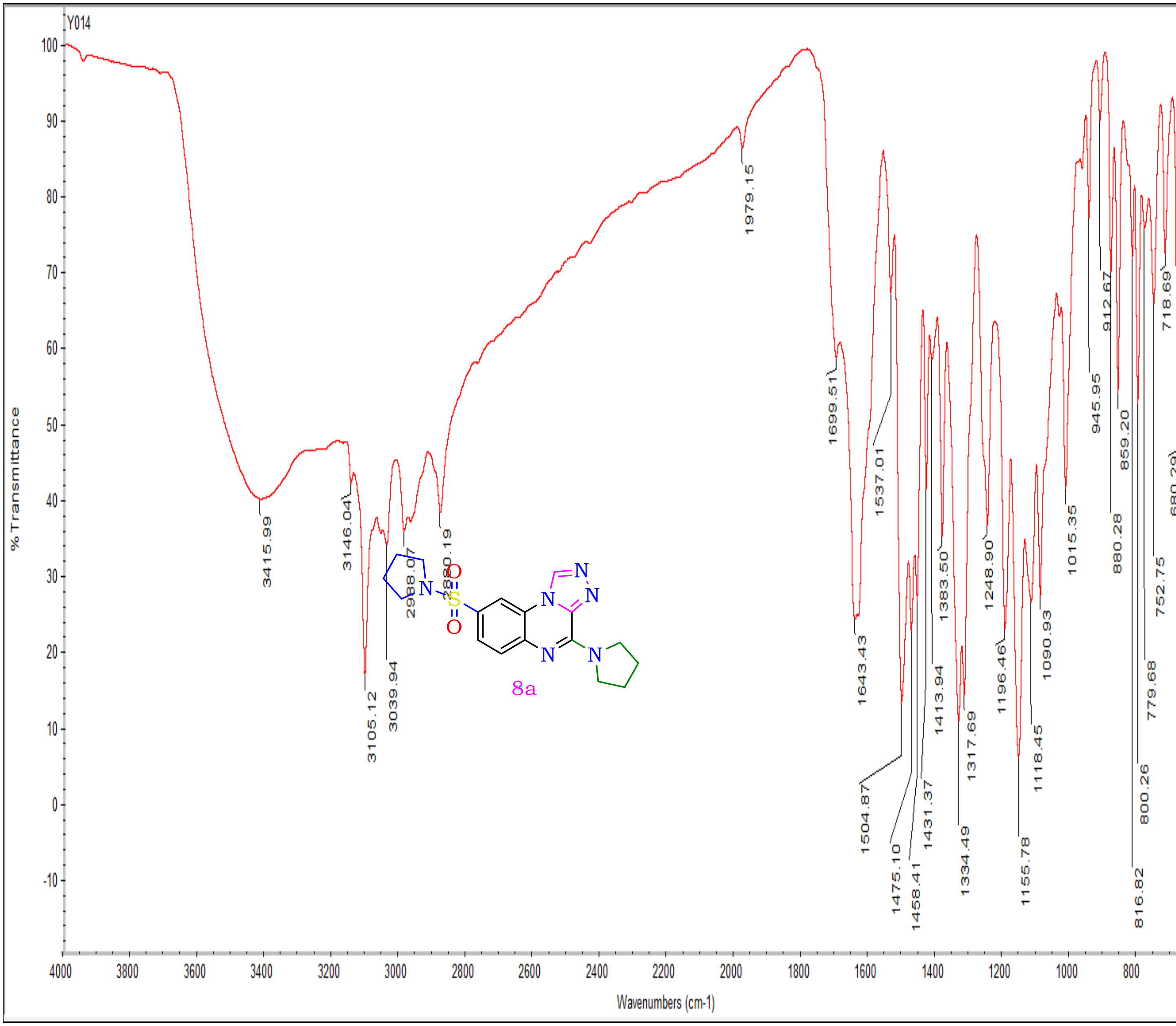

Figure SI6: IR spectrum of compound **8a**

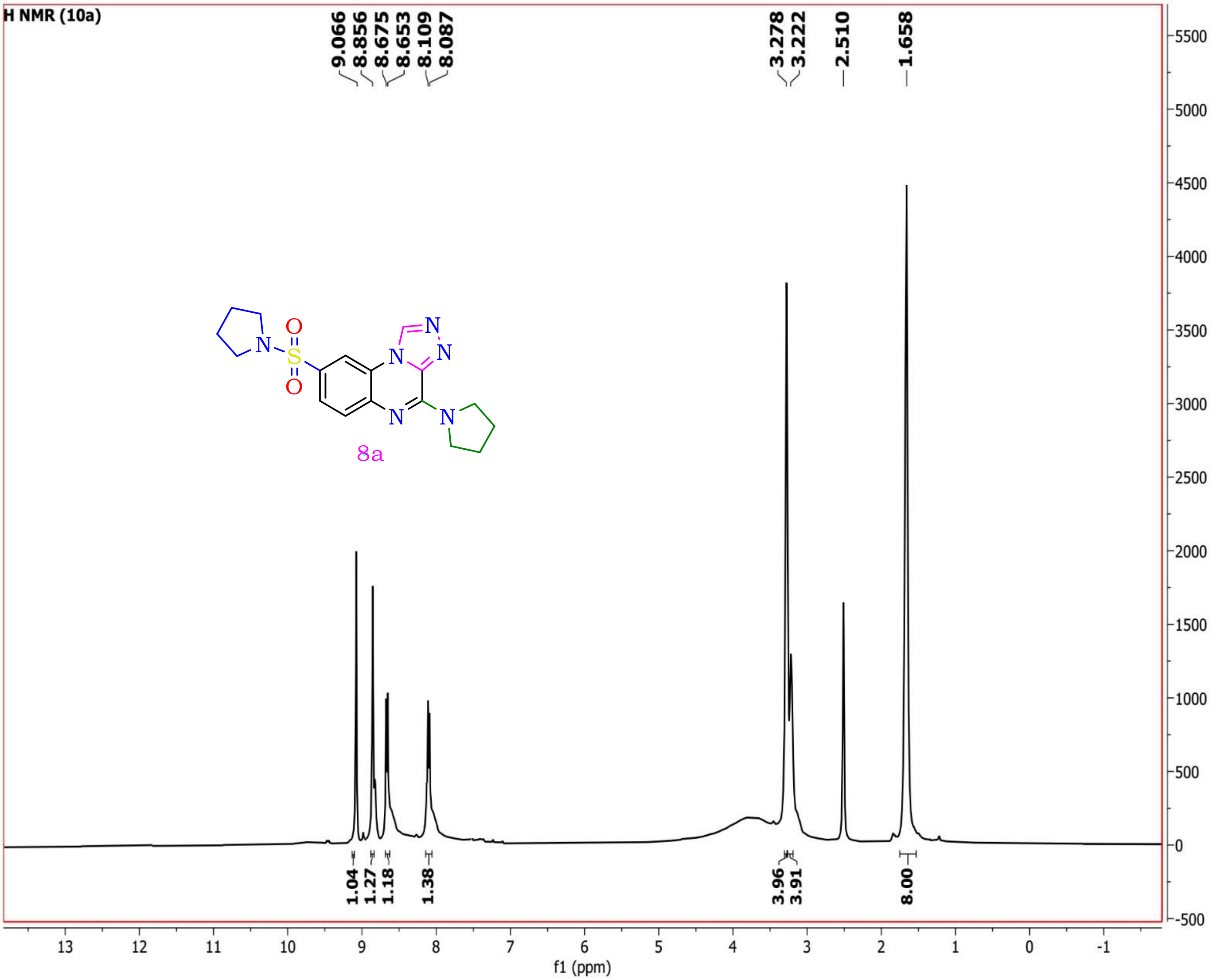

Figure SI7: ^{1}H NMR spectrum of compound **8a**

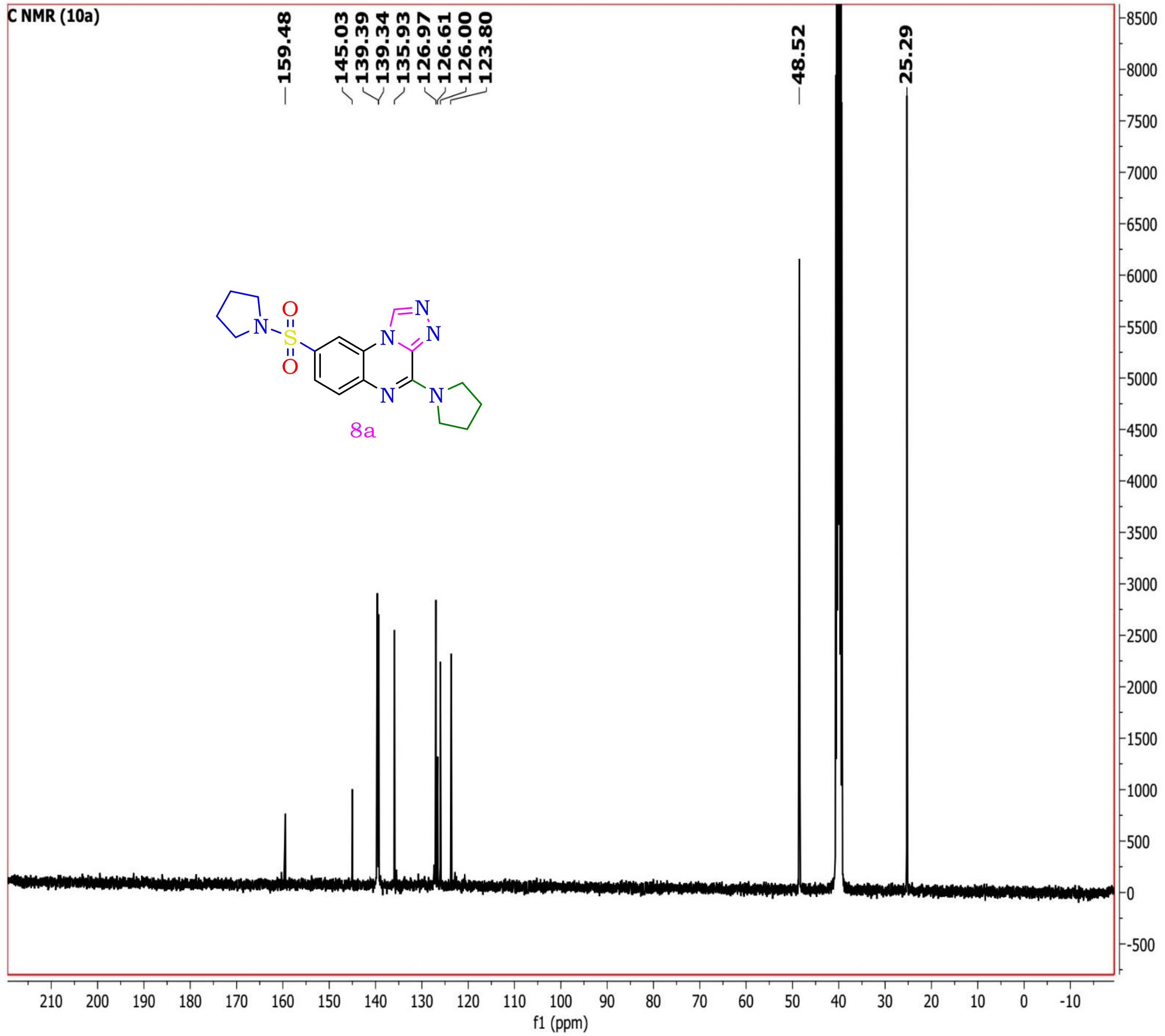

Figure SI8: ^{13}C NMR spectrum of compound **8a**

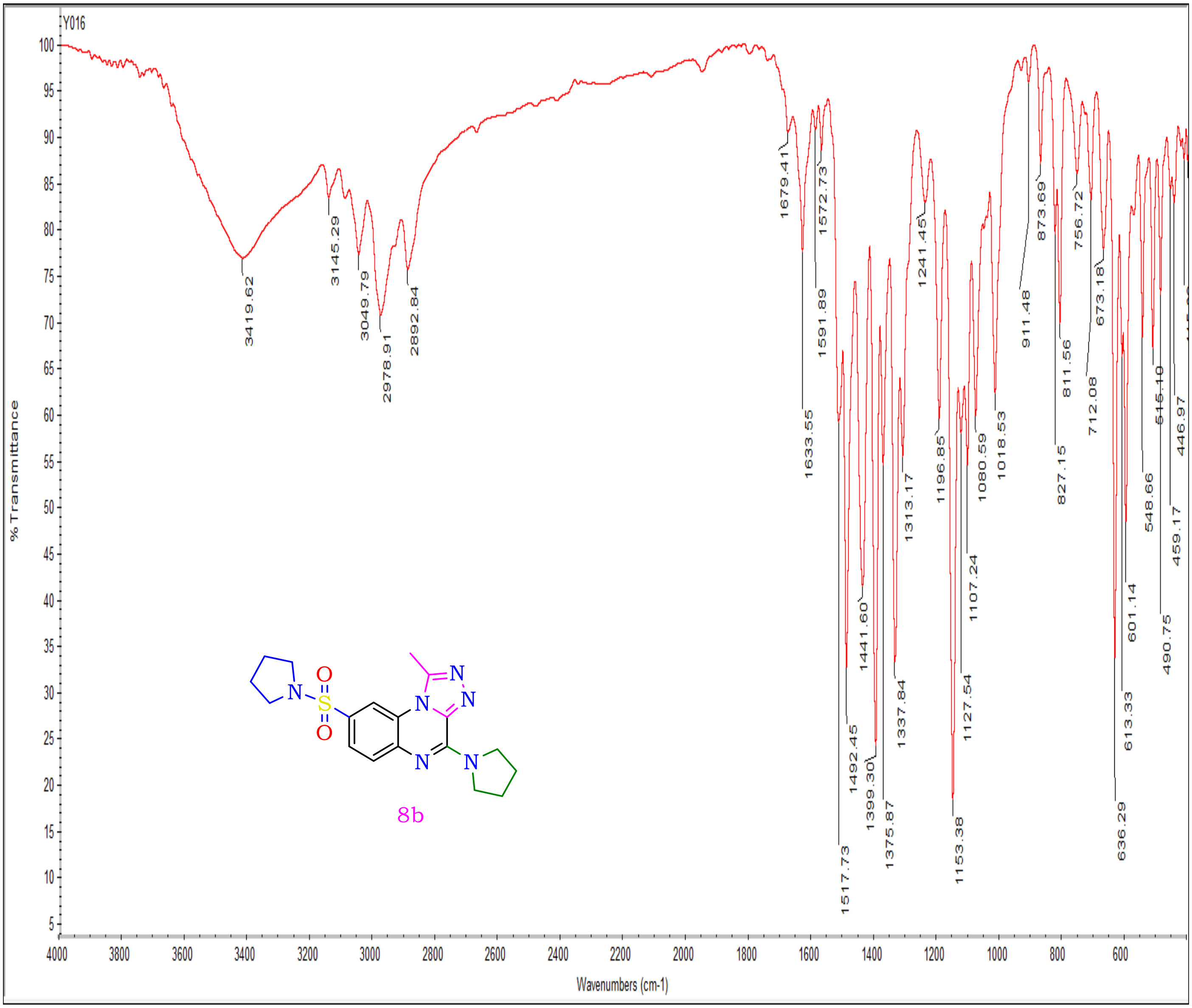

Figure SI9: IR spectrum of compound **8b**

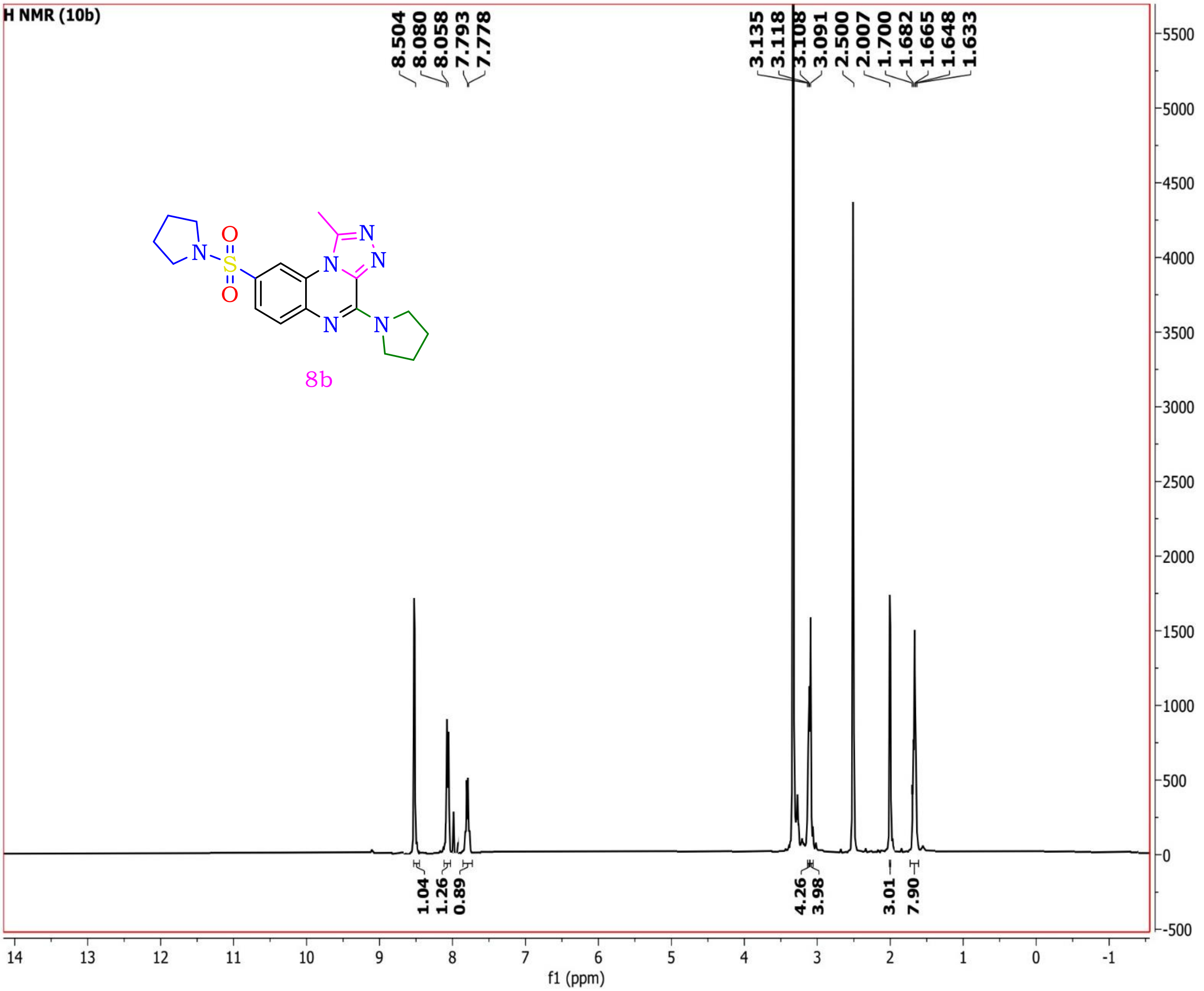

Figure SI10: ^{1}H NMR spectrum of compound **8b**

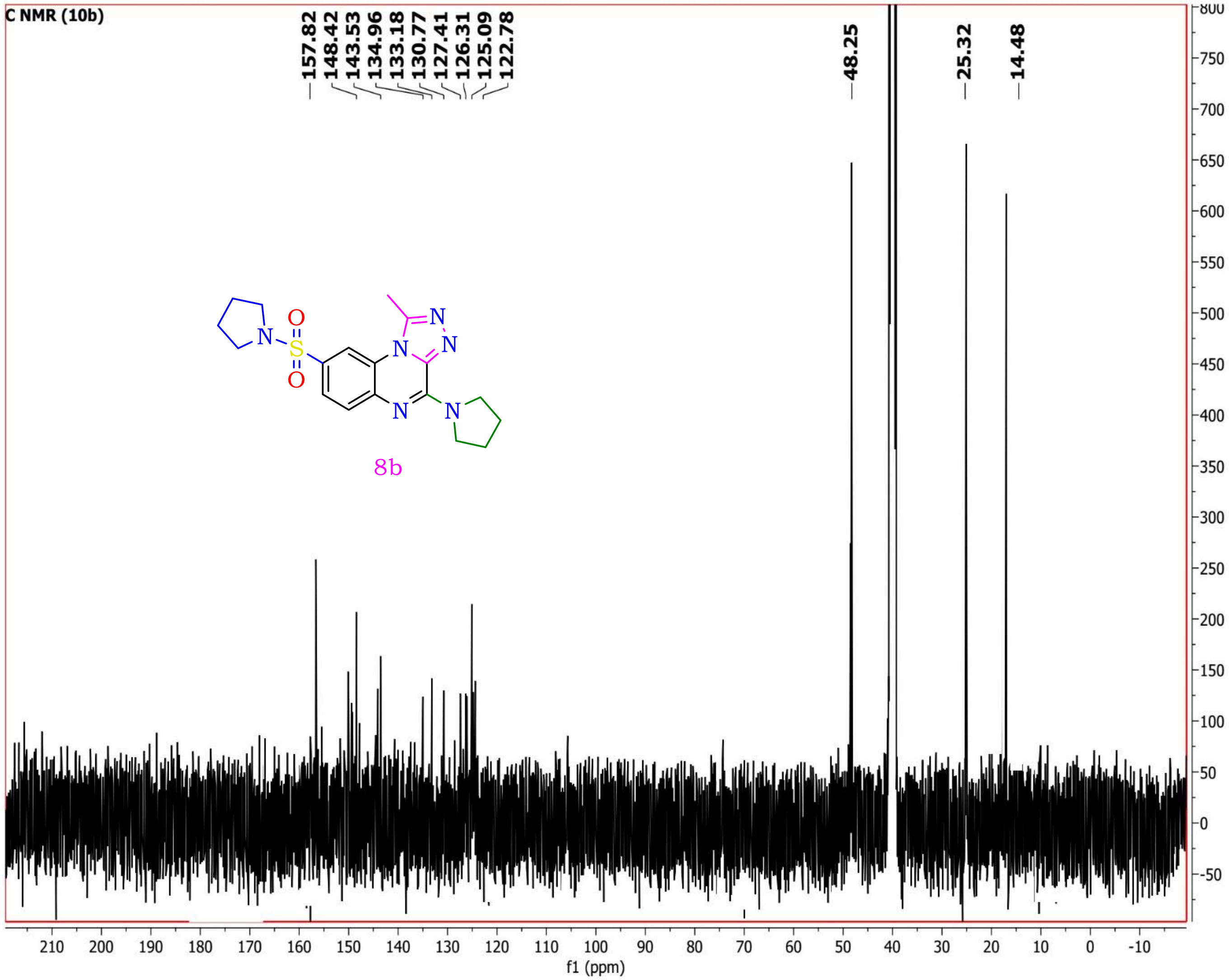

Figure SI1: ^{13}C NMR spectrum of compound **8b**

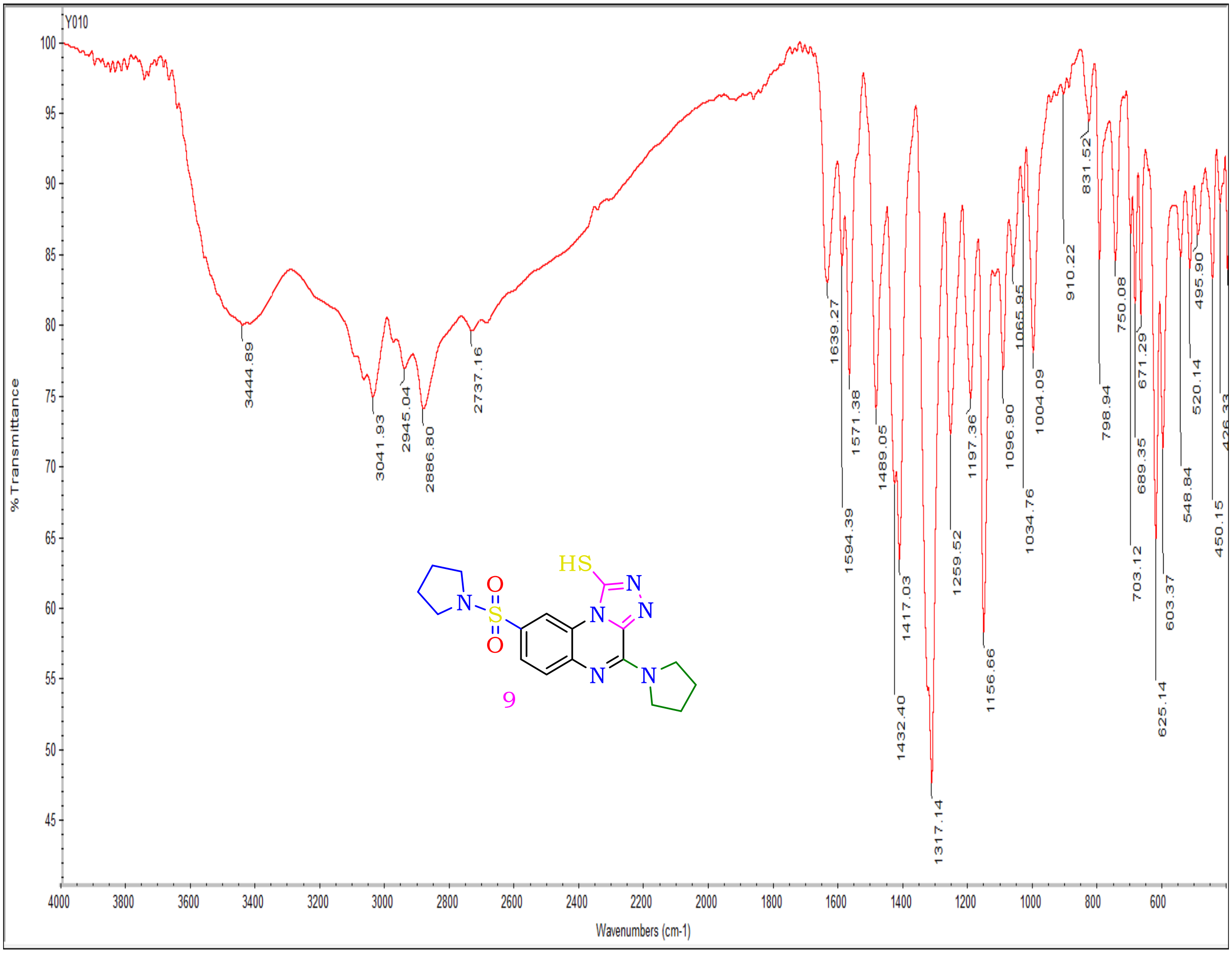

Figure SI12: IR spectrum of compound **9**

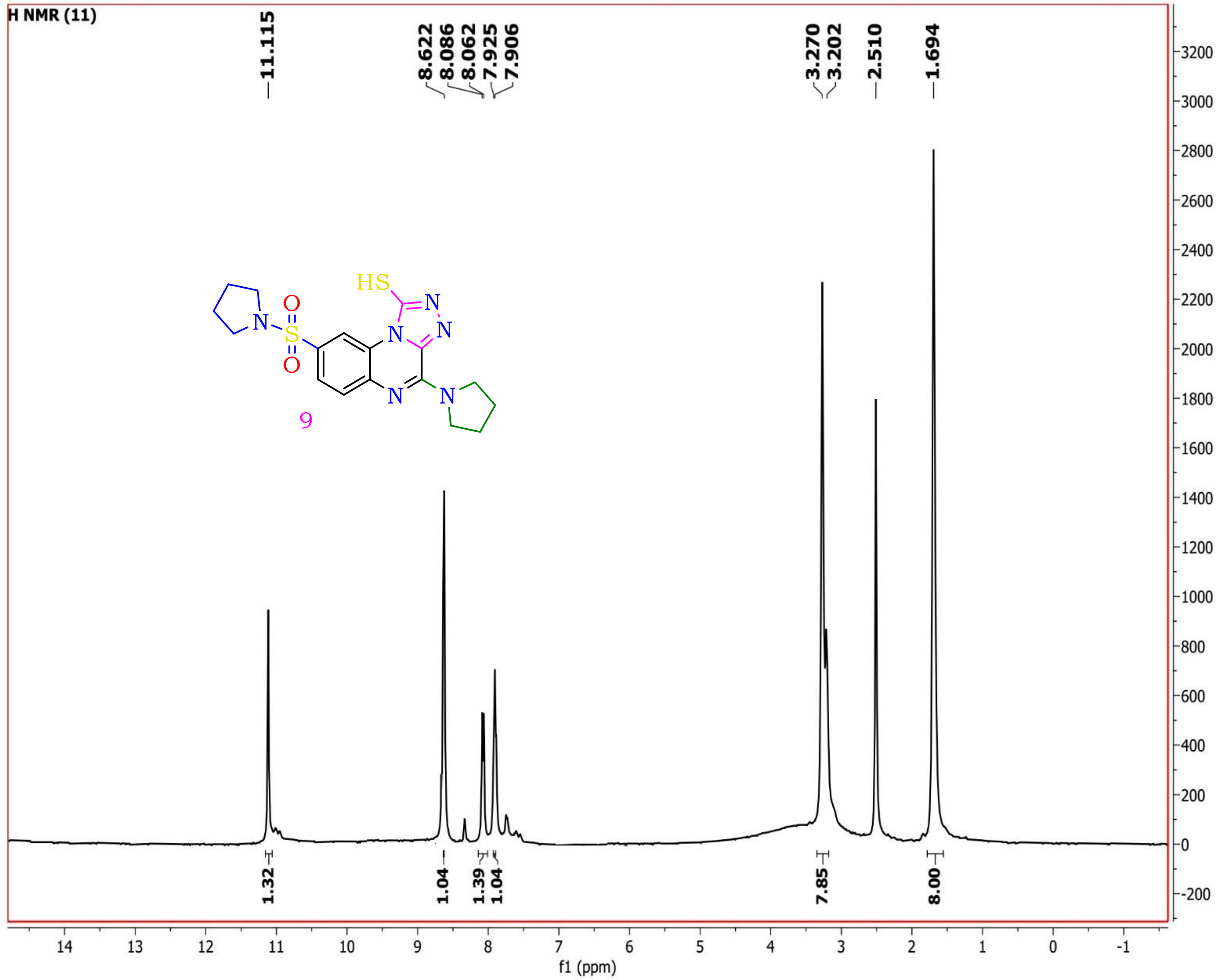

Figure SI13: ^{1}H NMR spectrum of compound **9**

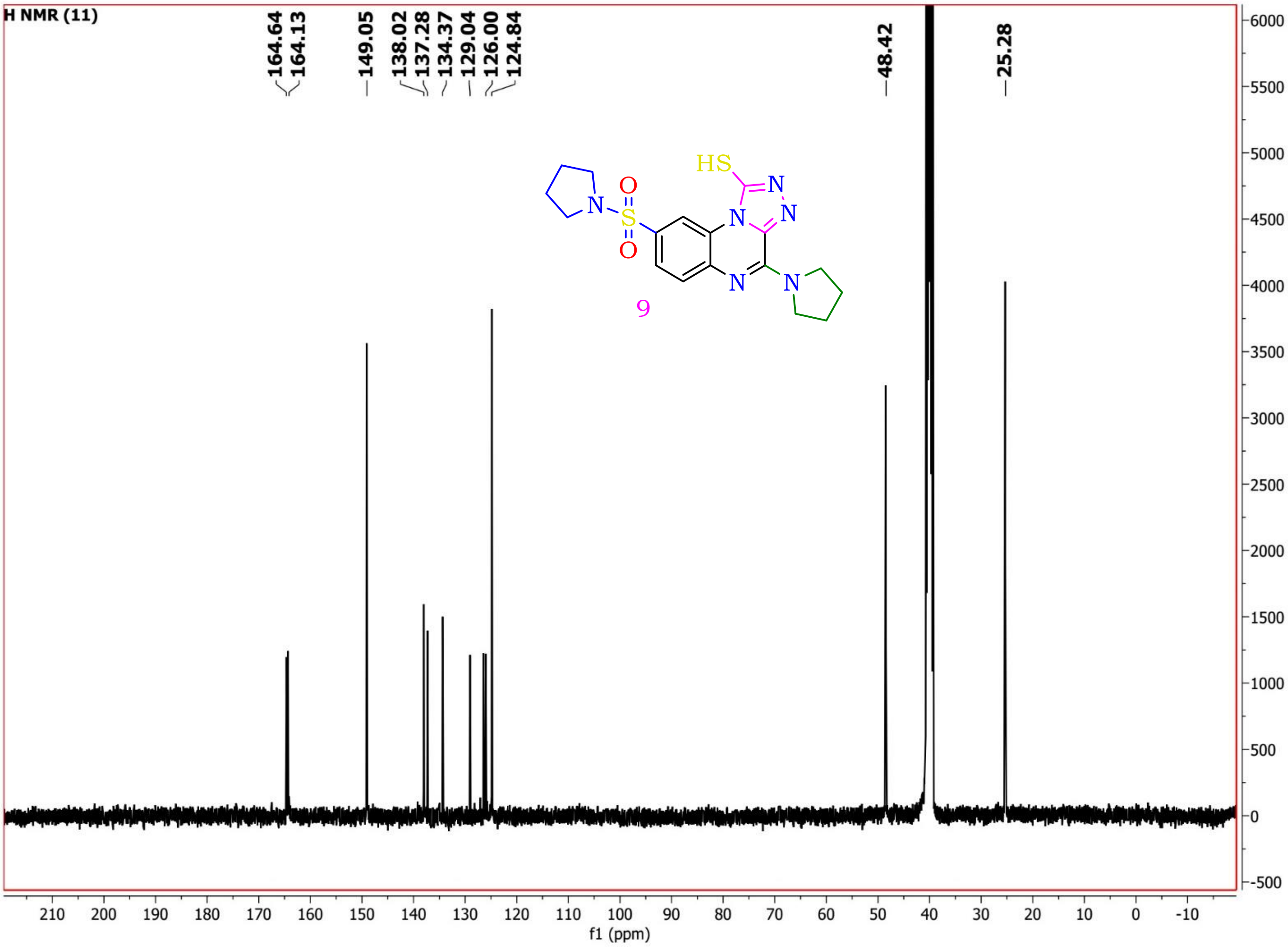

Figure SI14: ^{13}C NMR spectrum of compound **9**

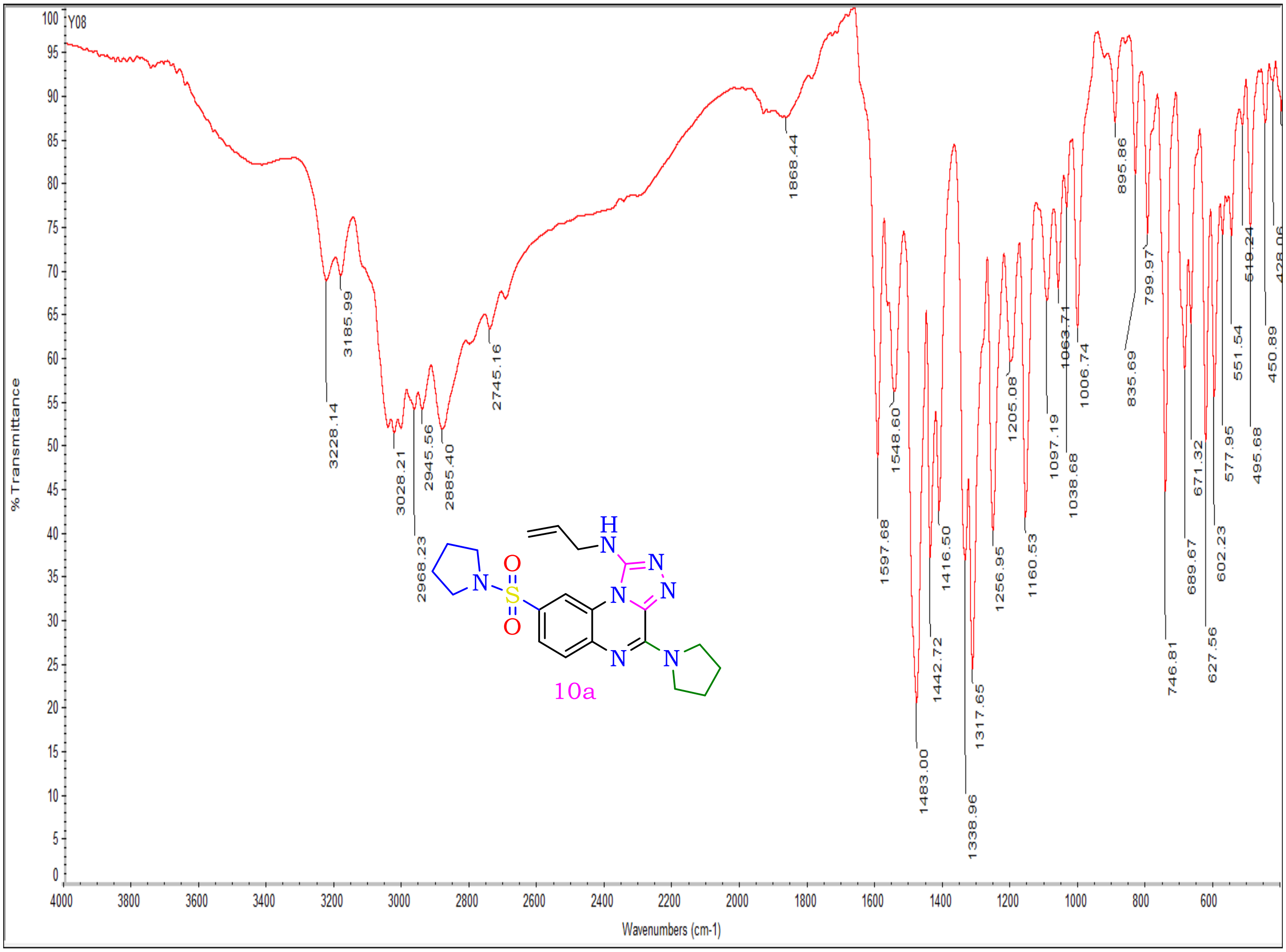

Figure SI15: IR spectrum of compound **10a**

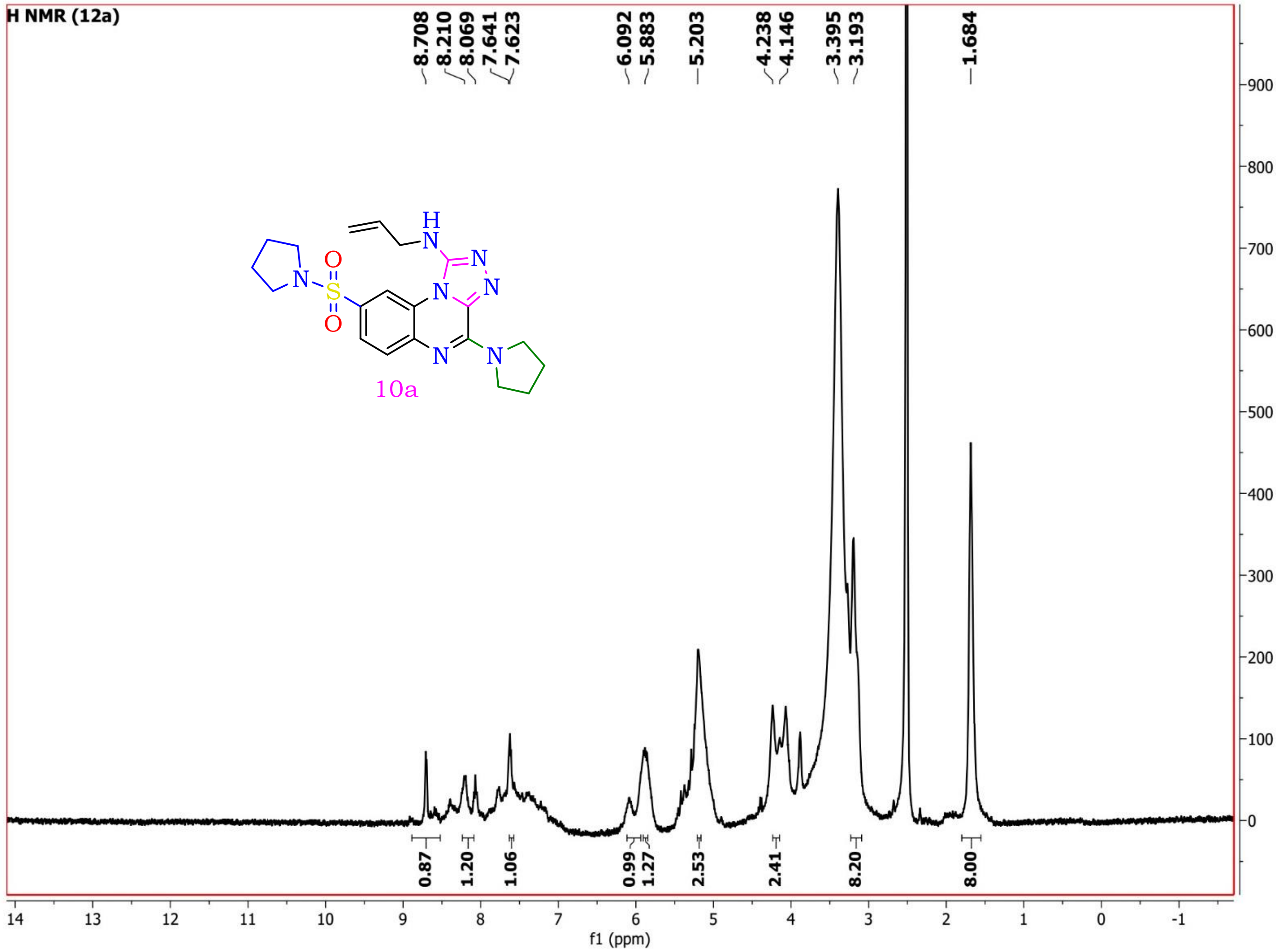

Figure SI16: ^{1}H NMR spectrum of compound **10a**

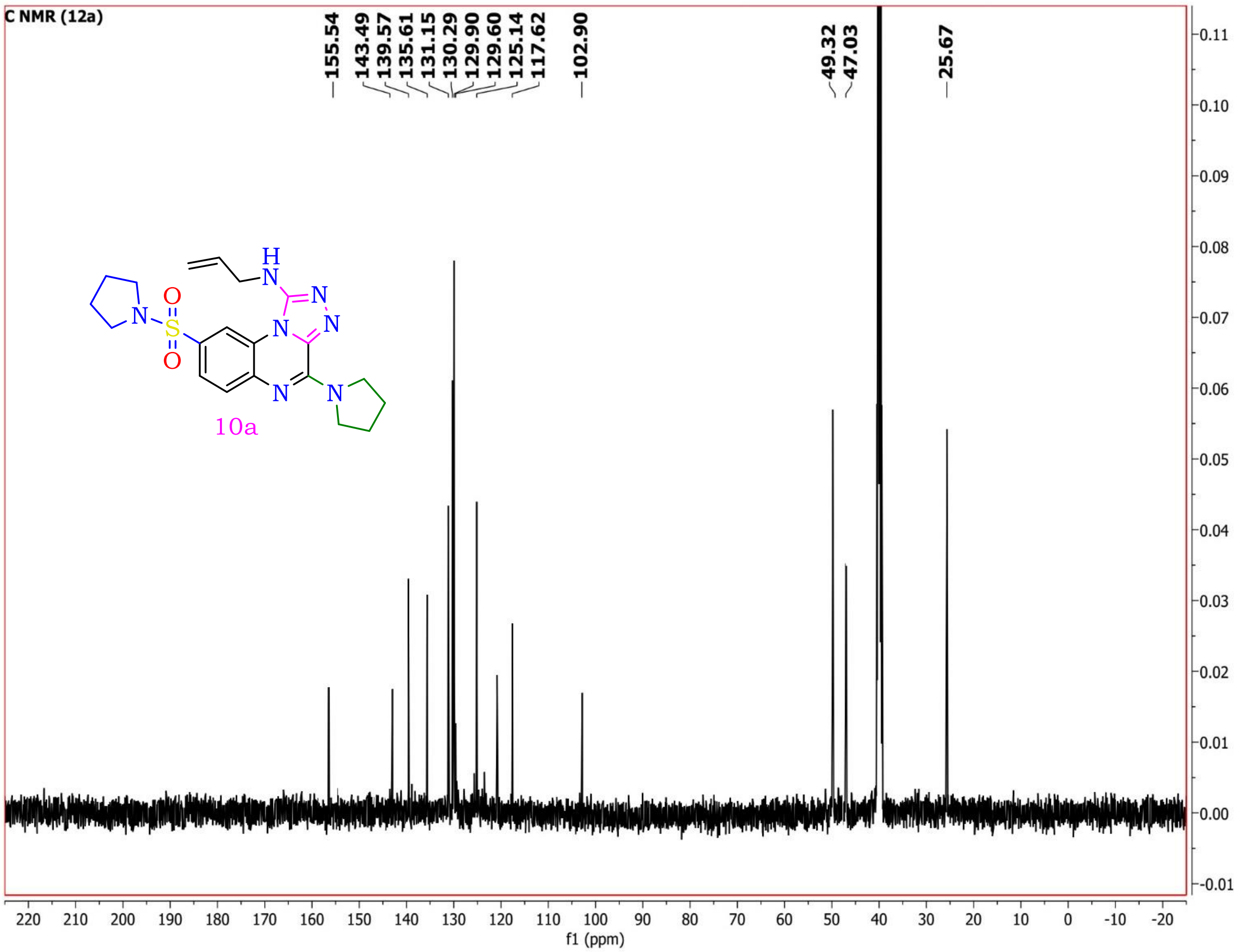

Figure SI17: ^{13}C NMR spectrum of compound **10a**

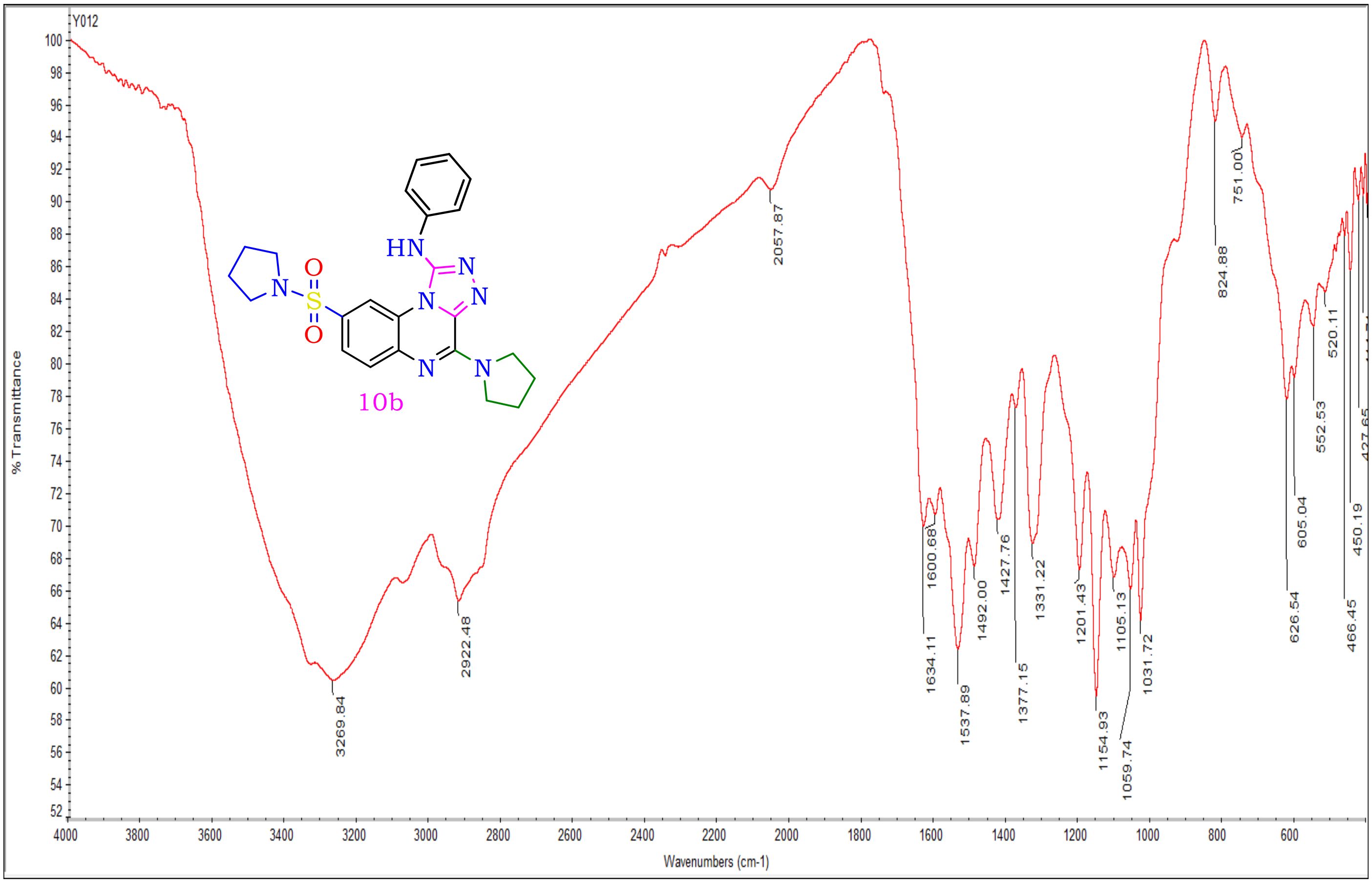

Figure SI18: IR spectrum of compound **10b**

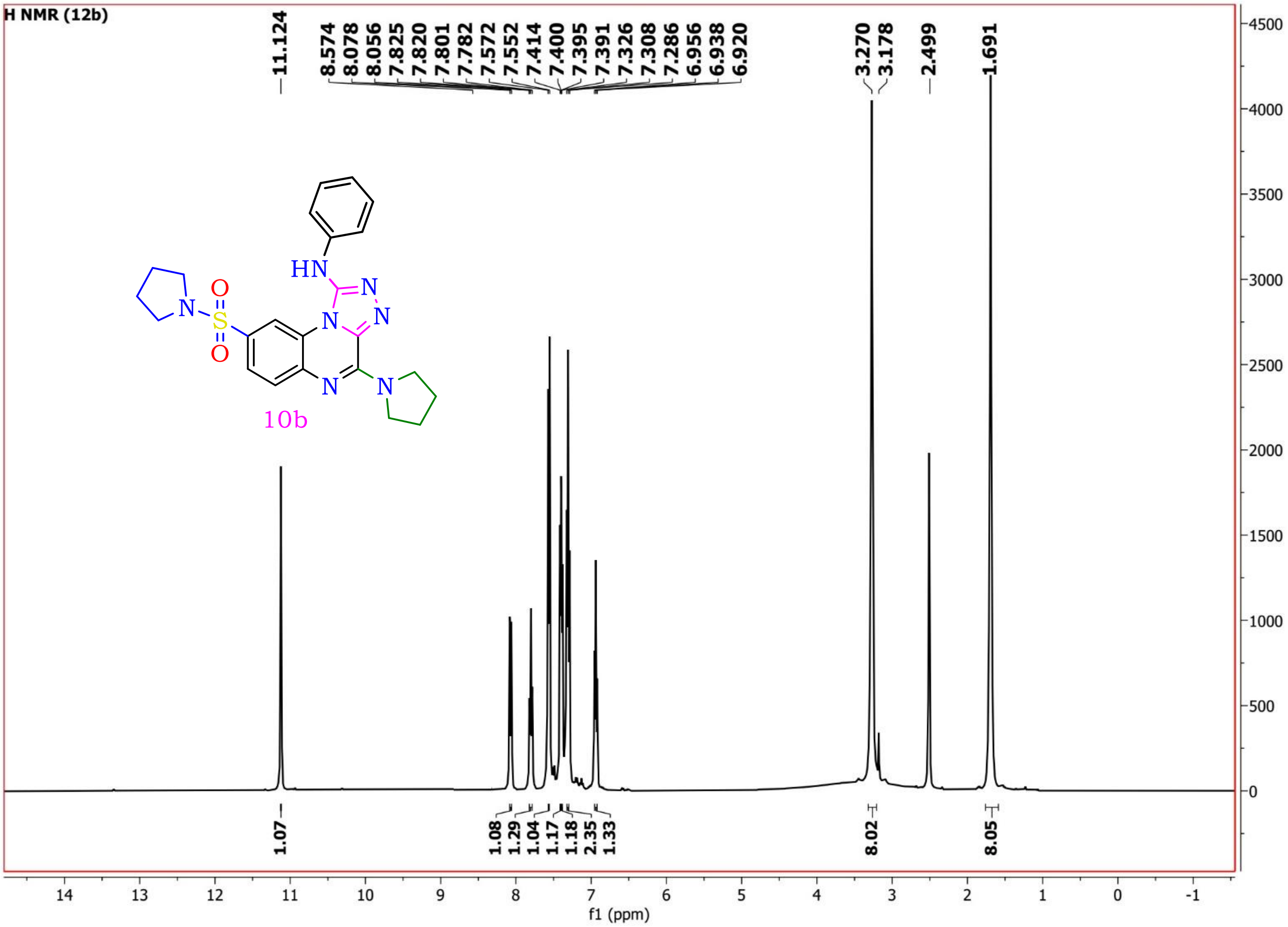

Figure SI19: ^{1}H NMR spectrum of compound **10b**

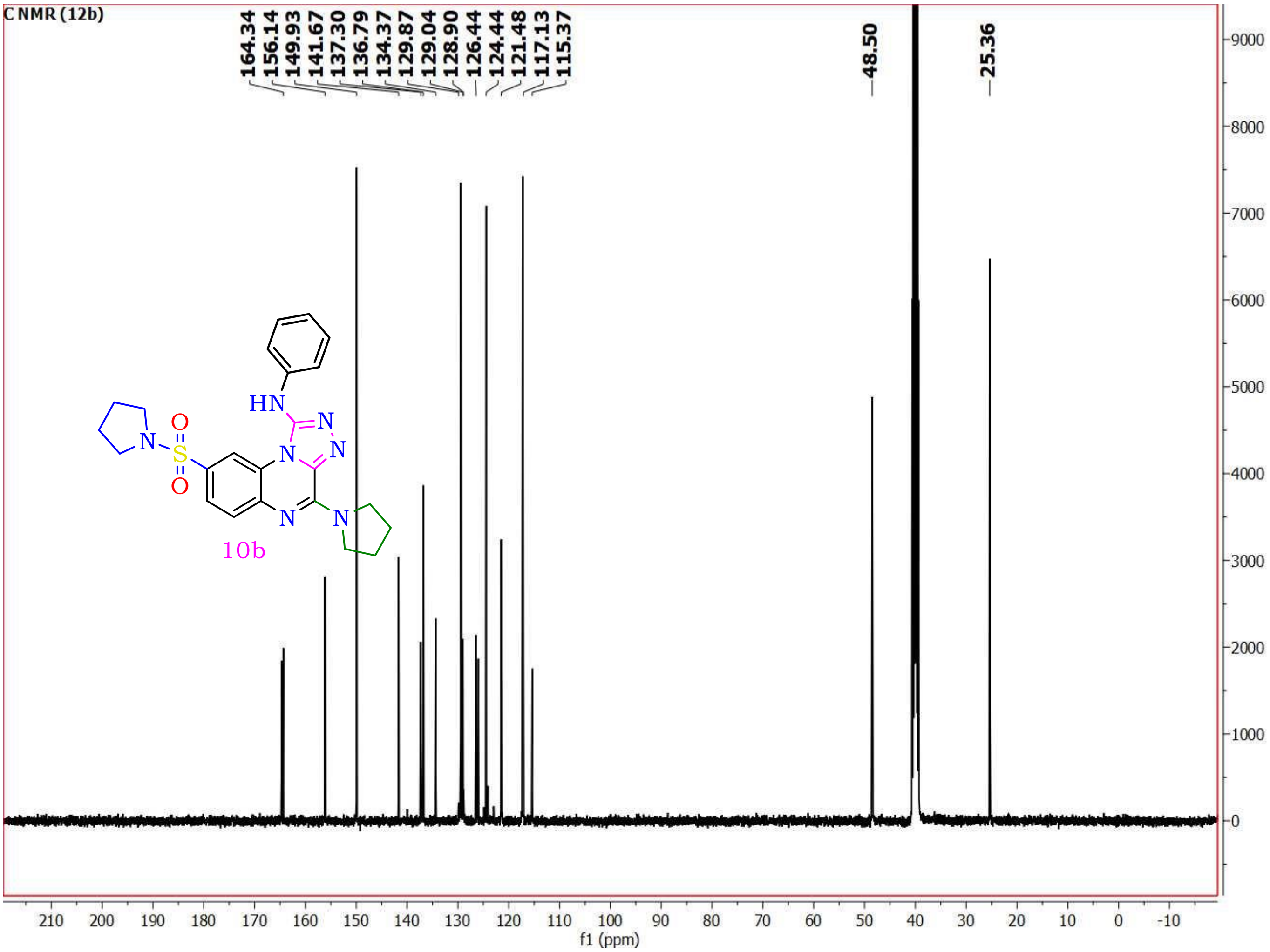

Figure SI20: ^{13}C NMR spectrum of compound **10b**

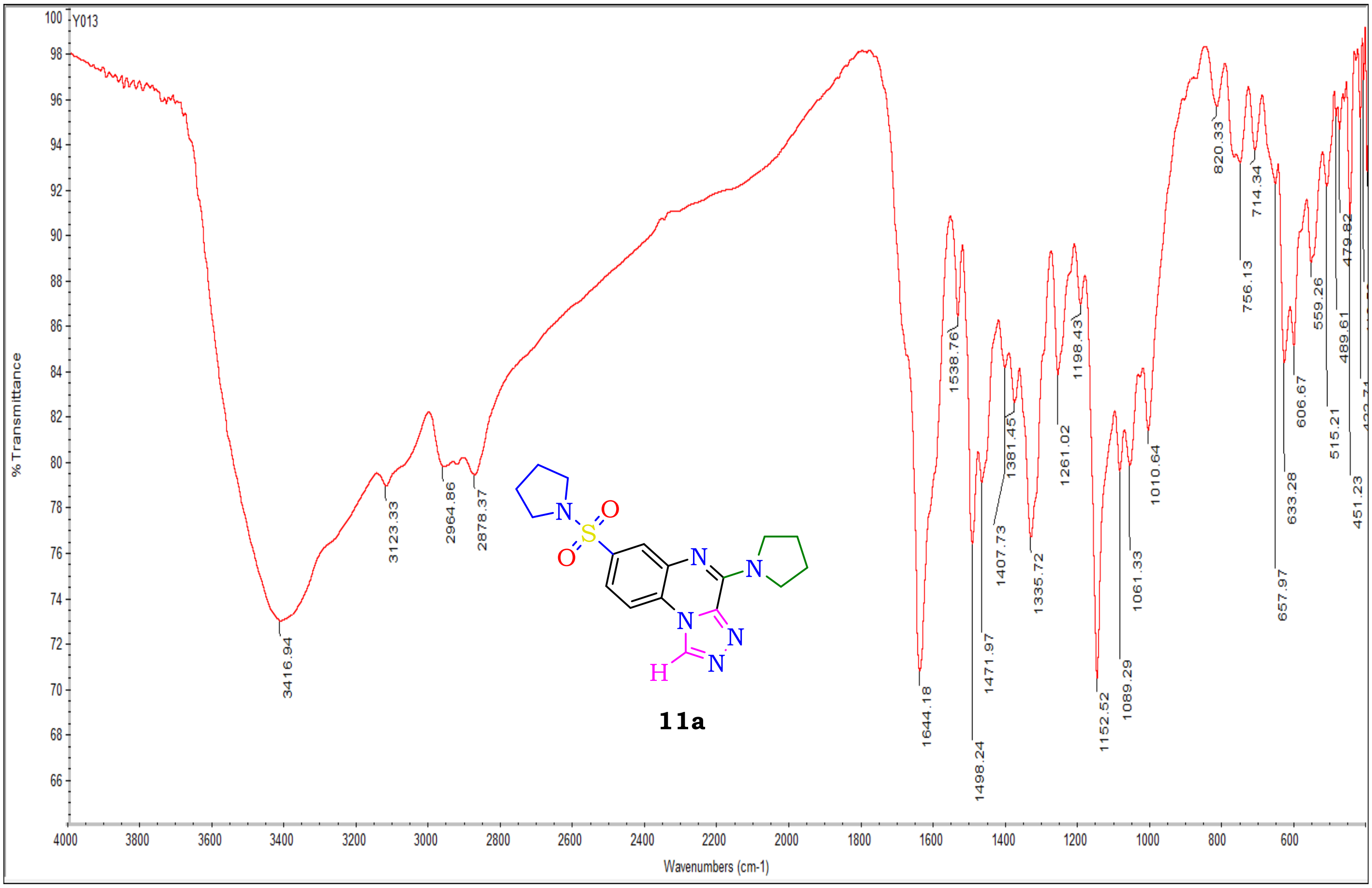

11a

Figure SI21: IR spectrum of compound **11a**

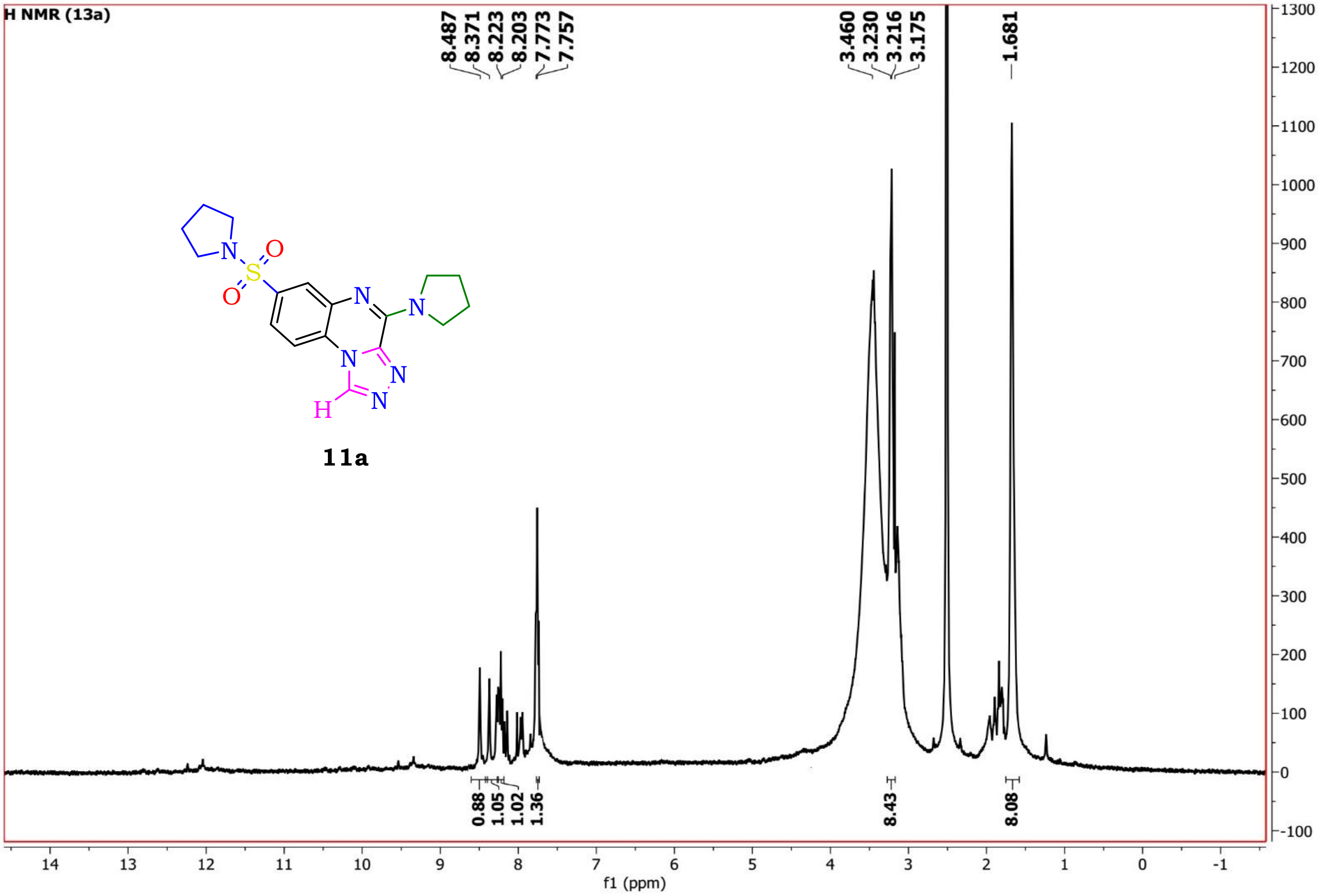

Figure SI22: ^{1}H NMR spectrum of compound **11a**

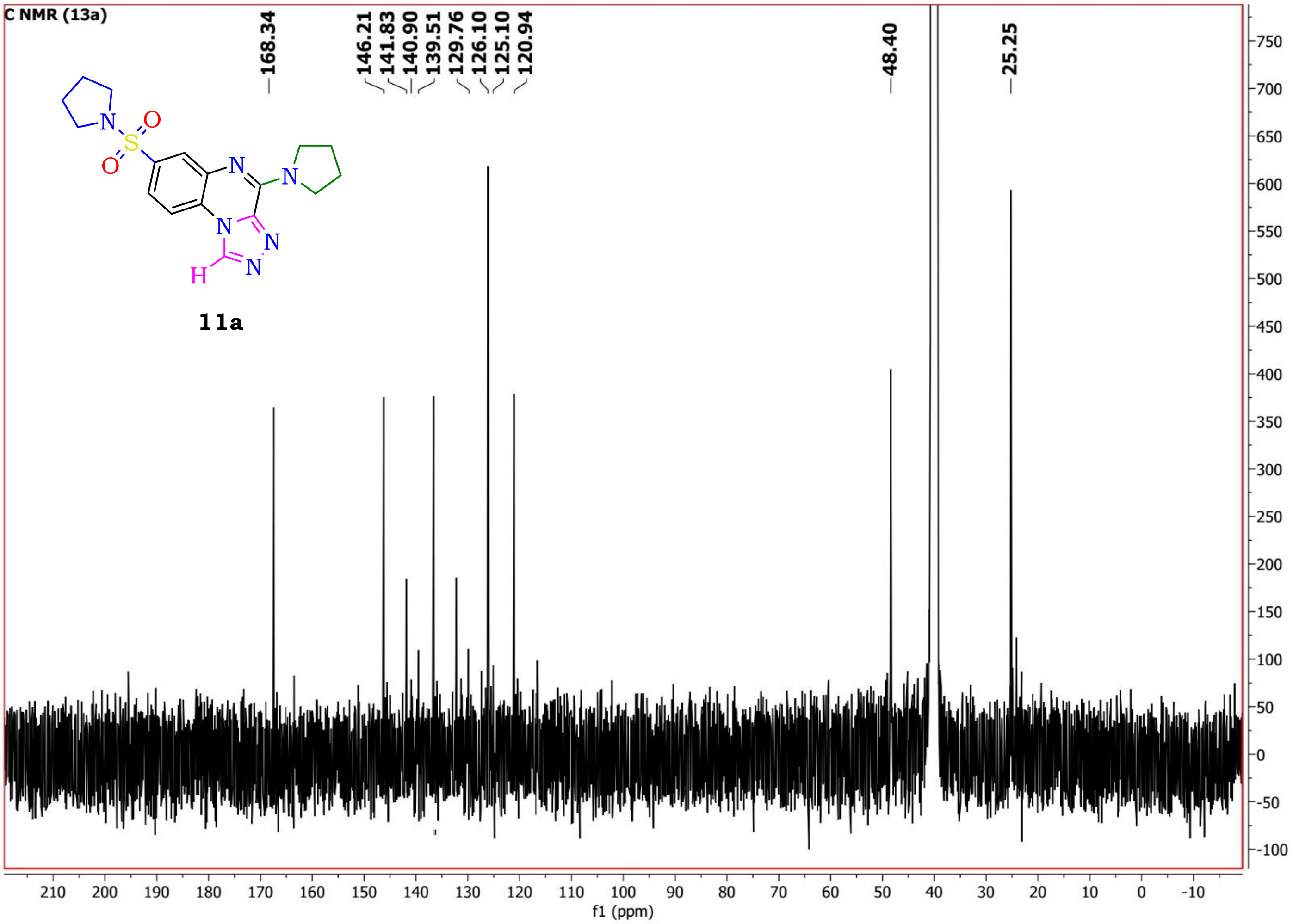

Figure SI23: ^{13}C NMR spectrum of compound **11a**

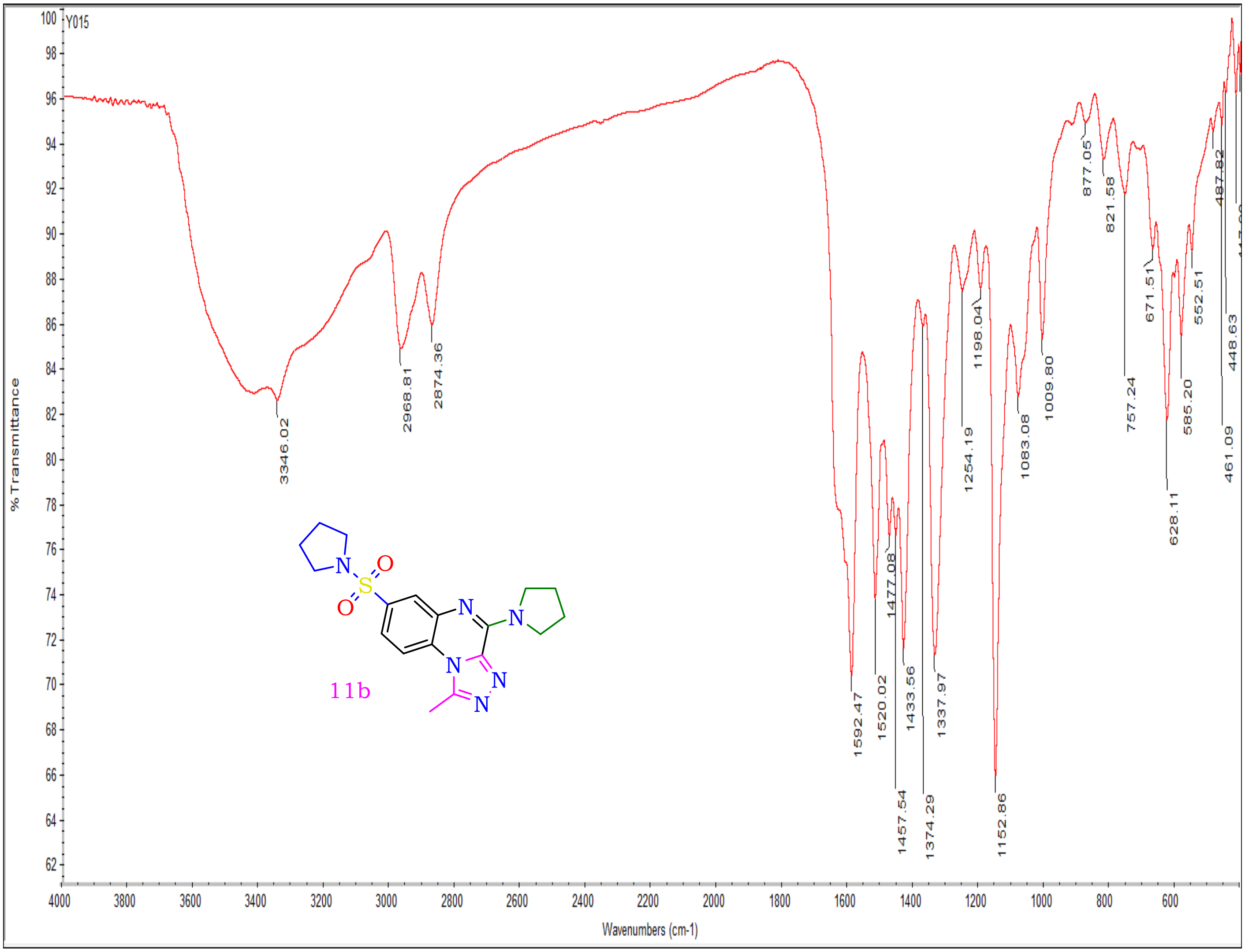

Figure SI24: IR spectrum of compound **11b**

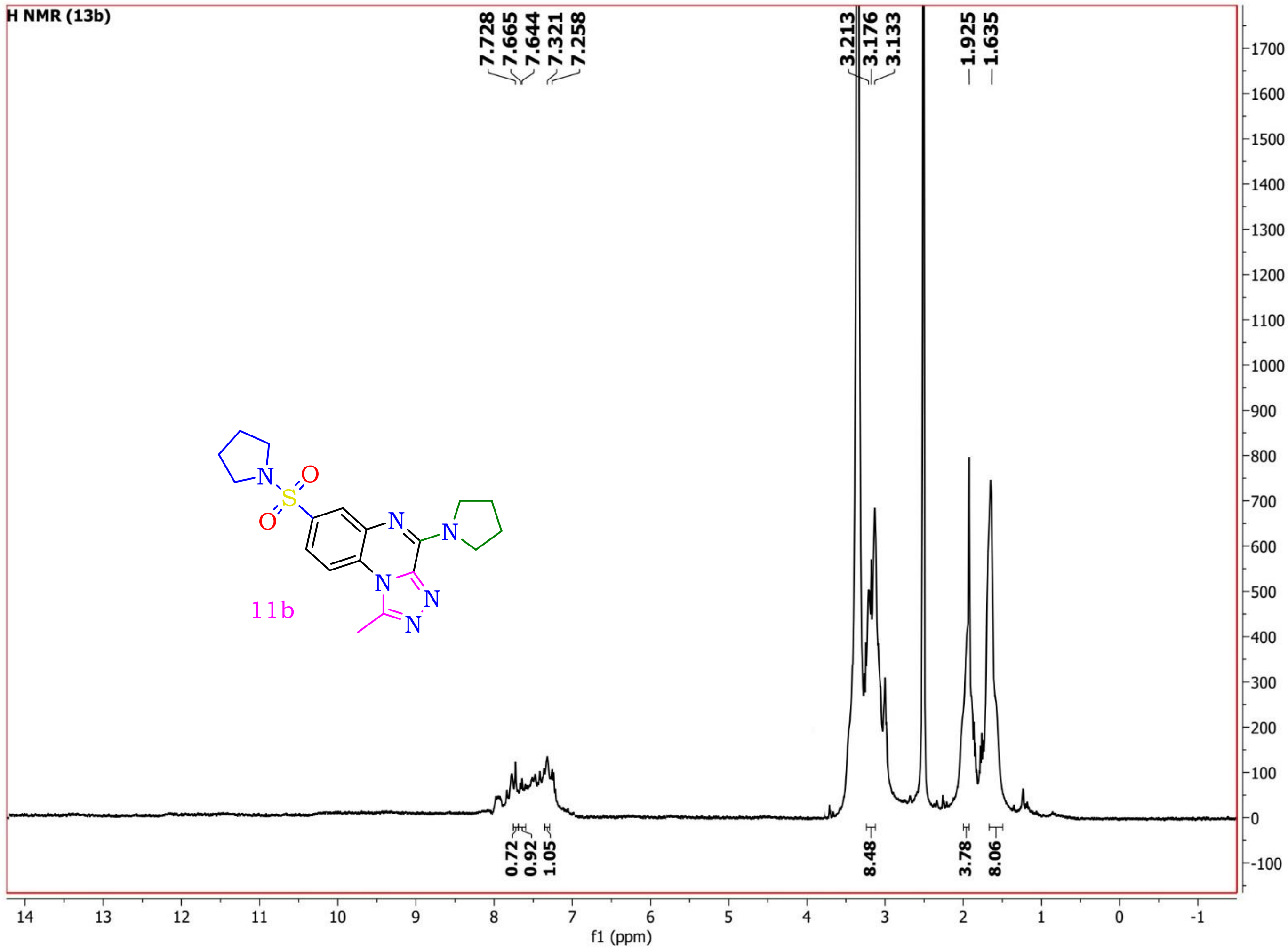

Figure SI25: ^{1}H NMR spectrum of compound **11b**

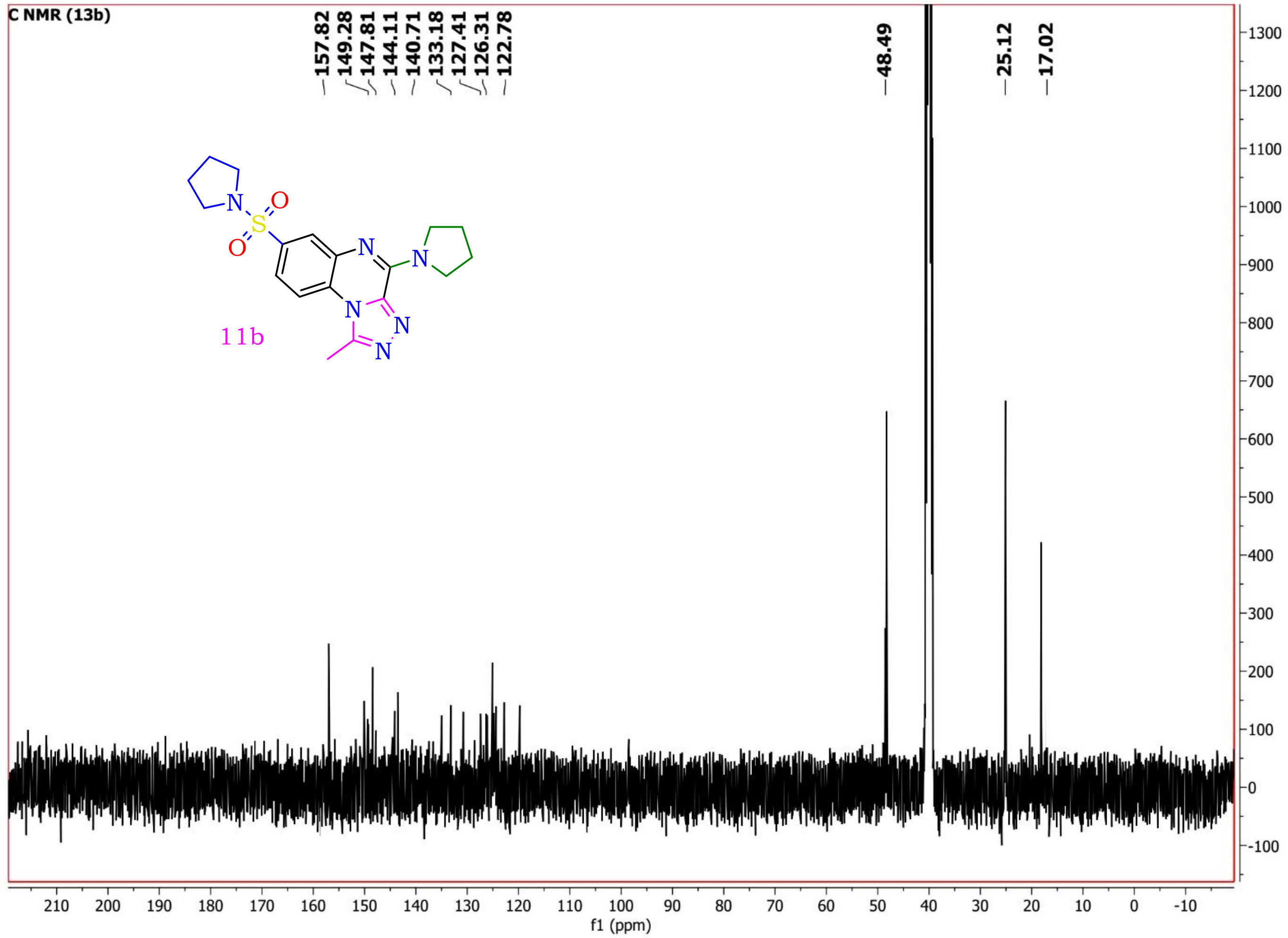

Figure SI26: ^{13}C NMR spectrum of compound **11b**

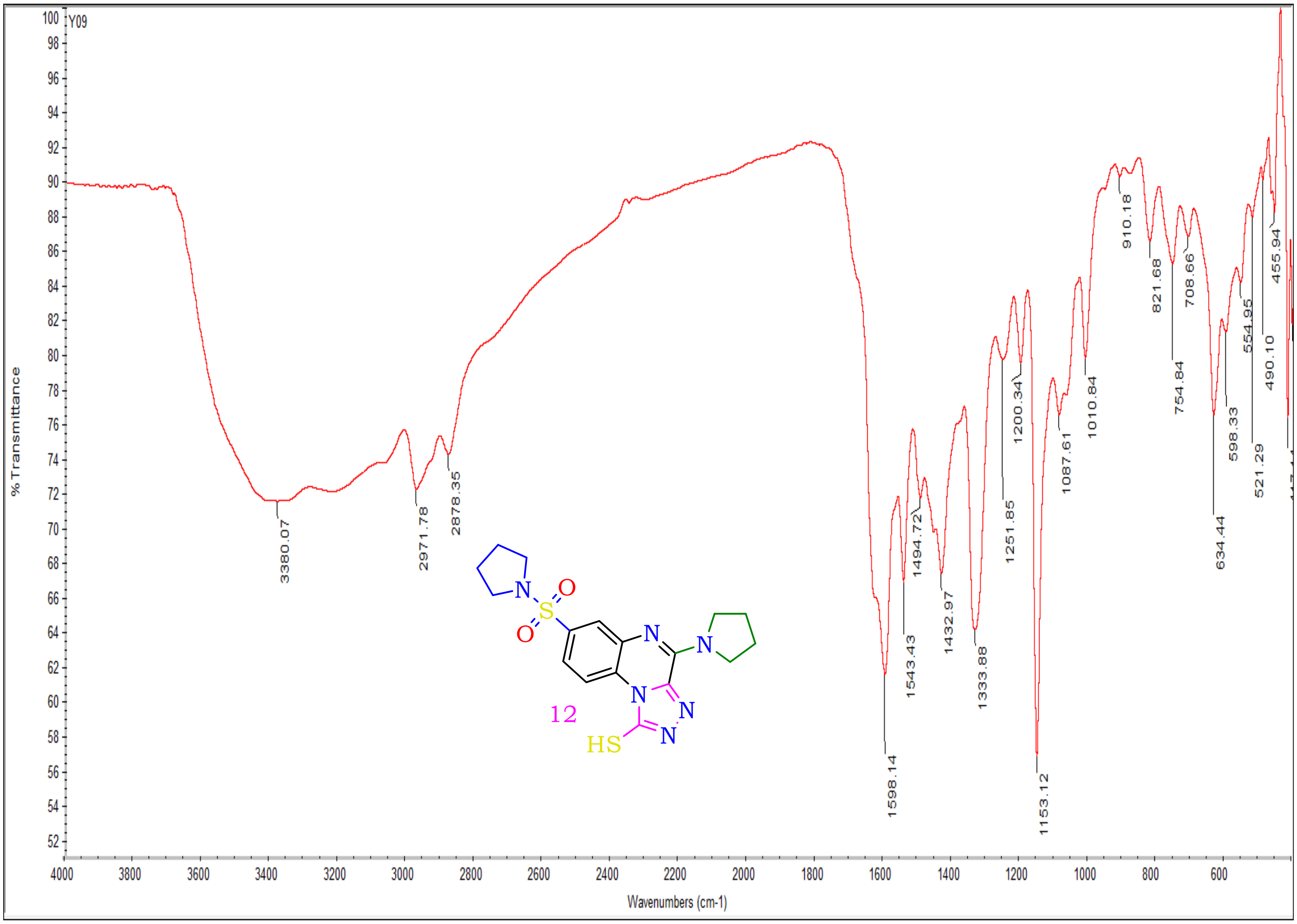

Figure SI27: IR spectrum of compound **12**

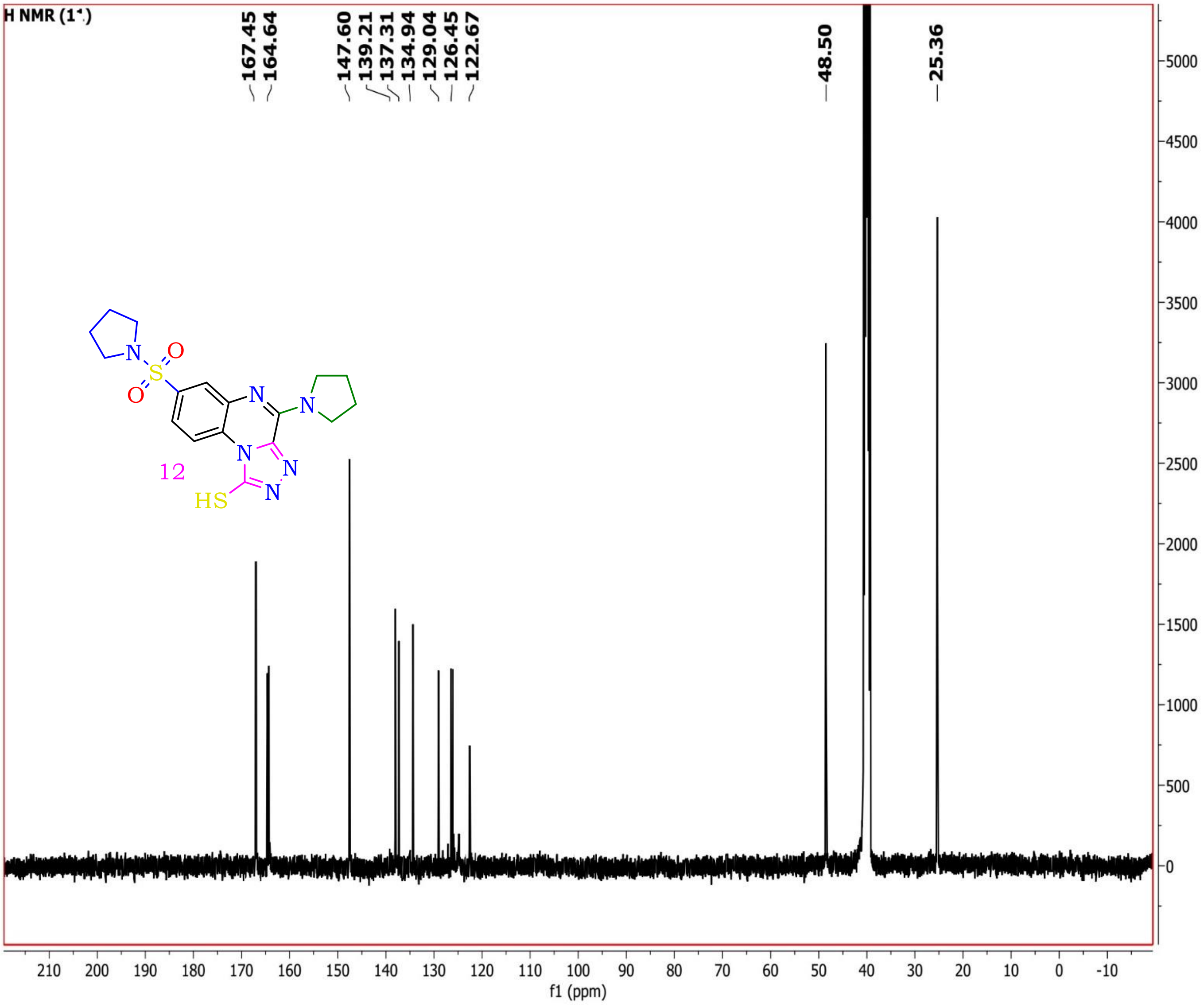

Figure SI28: ^{13}C NMR spectrum of compound **12**

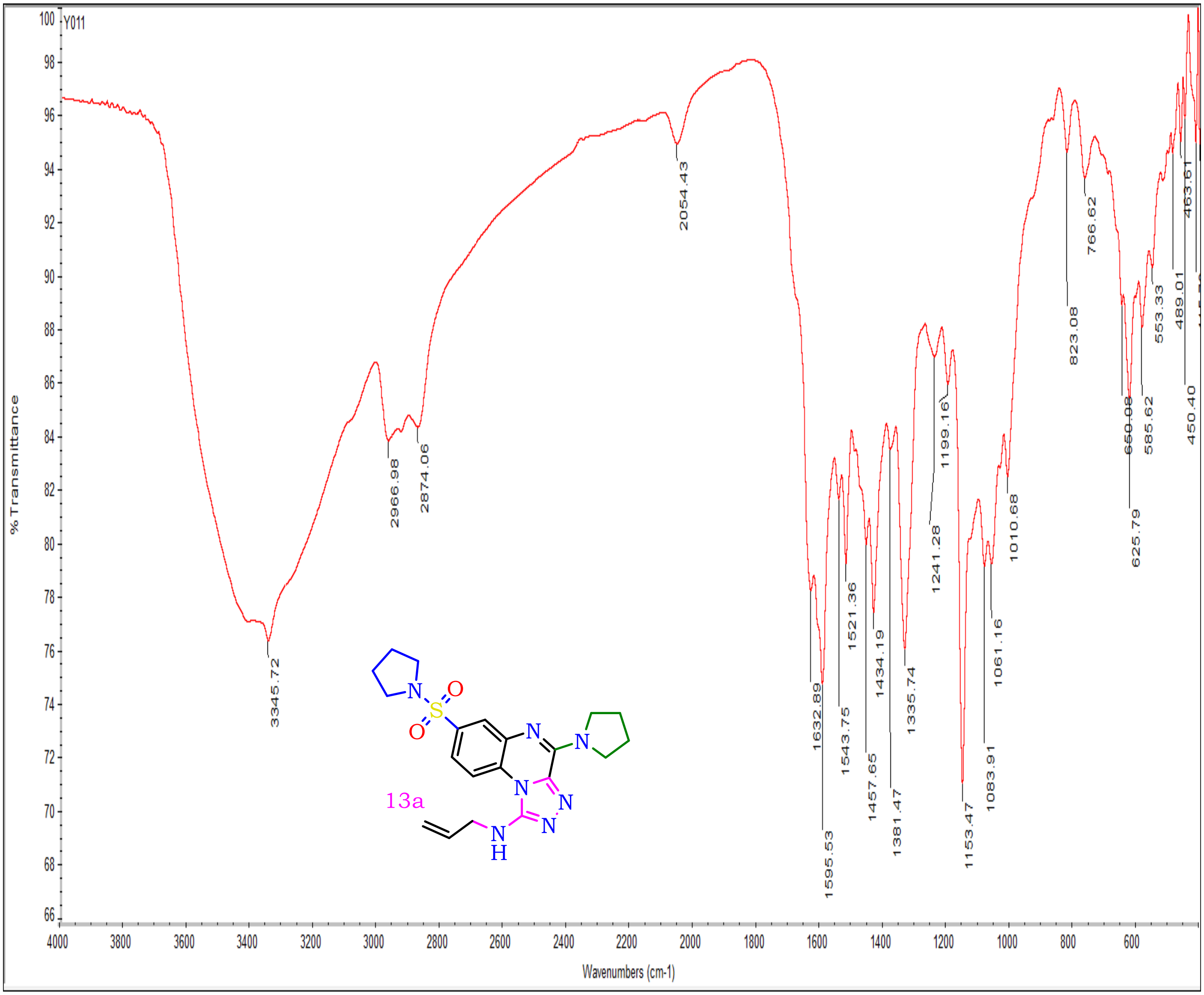

Figure SI29: IR spectrum of compound **13a**

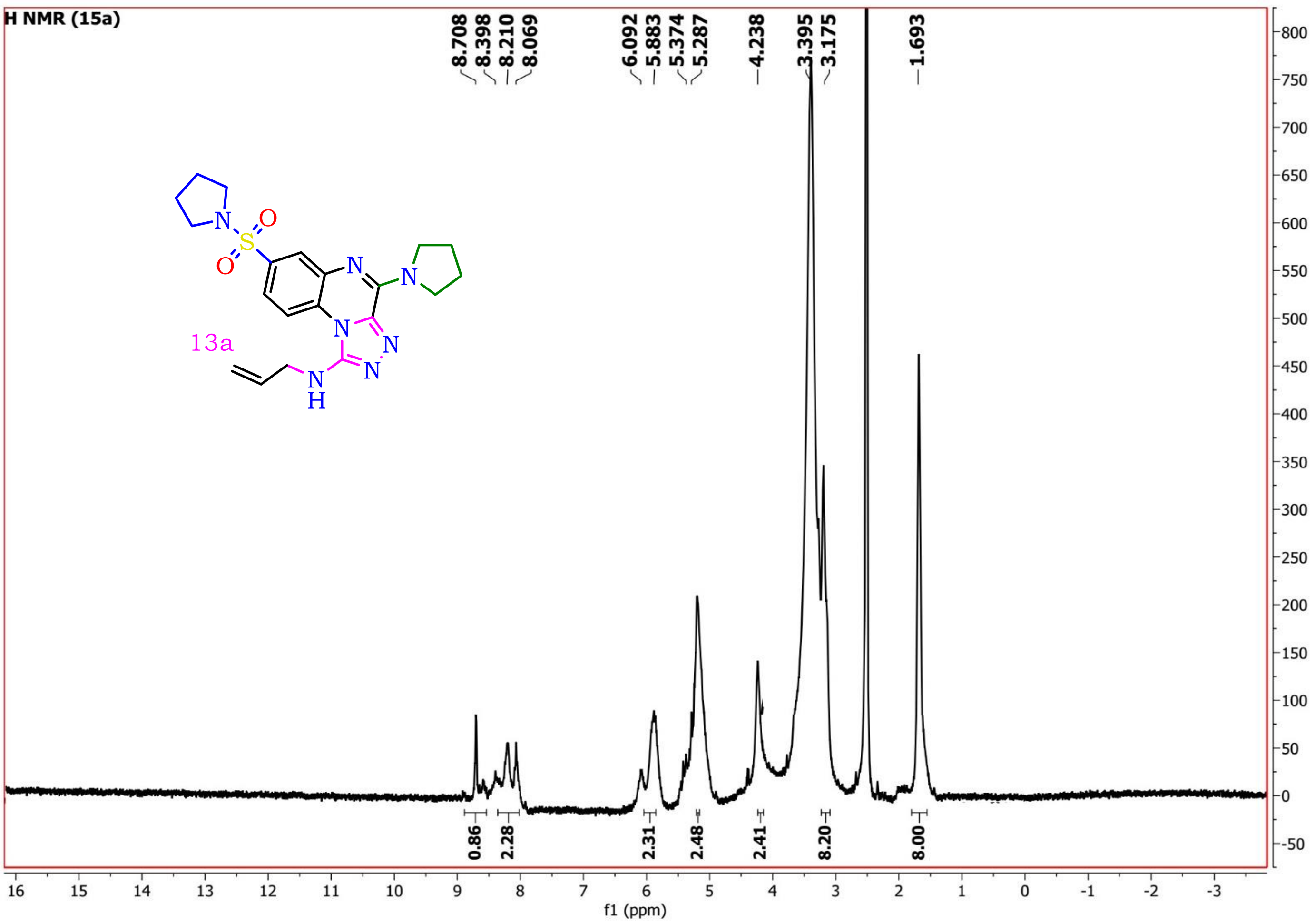

Figure SI30: ^{1}H NMR spectrum of compound 1**3a**

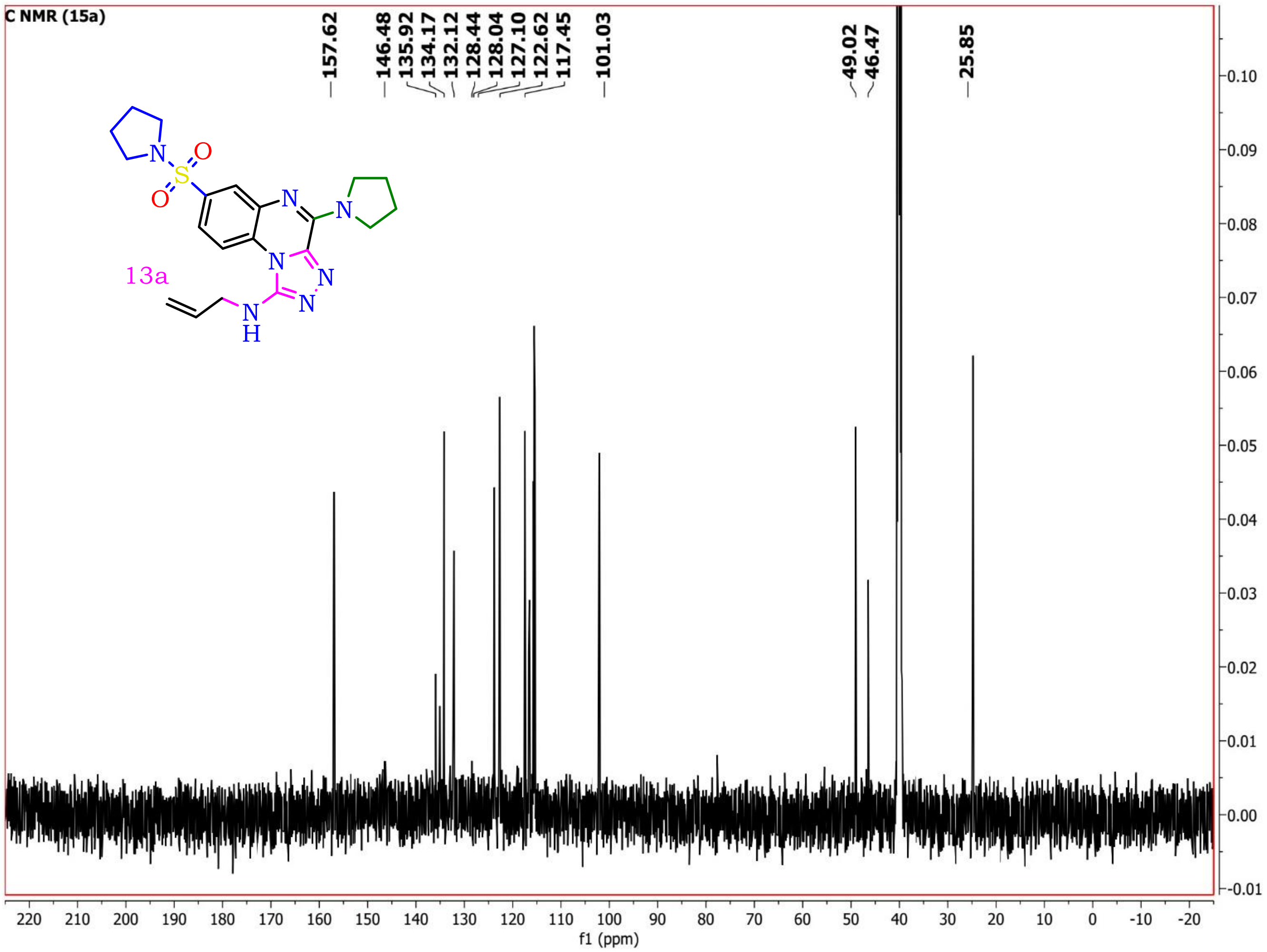

Figure SI31: ^{13}C NMR spectrum of compound 1**3a**

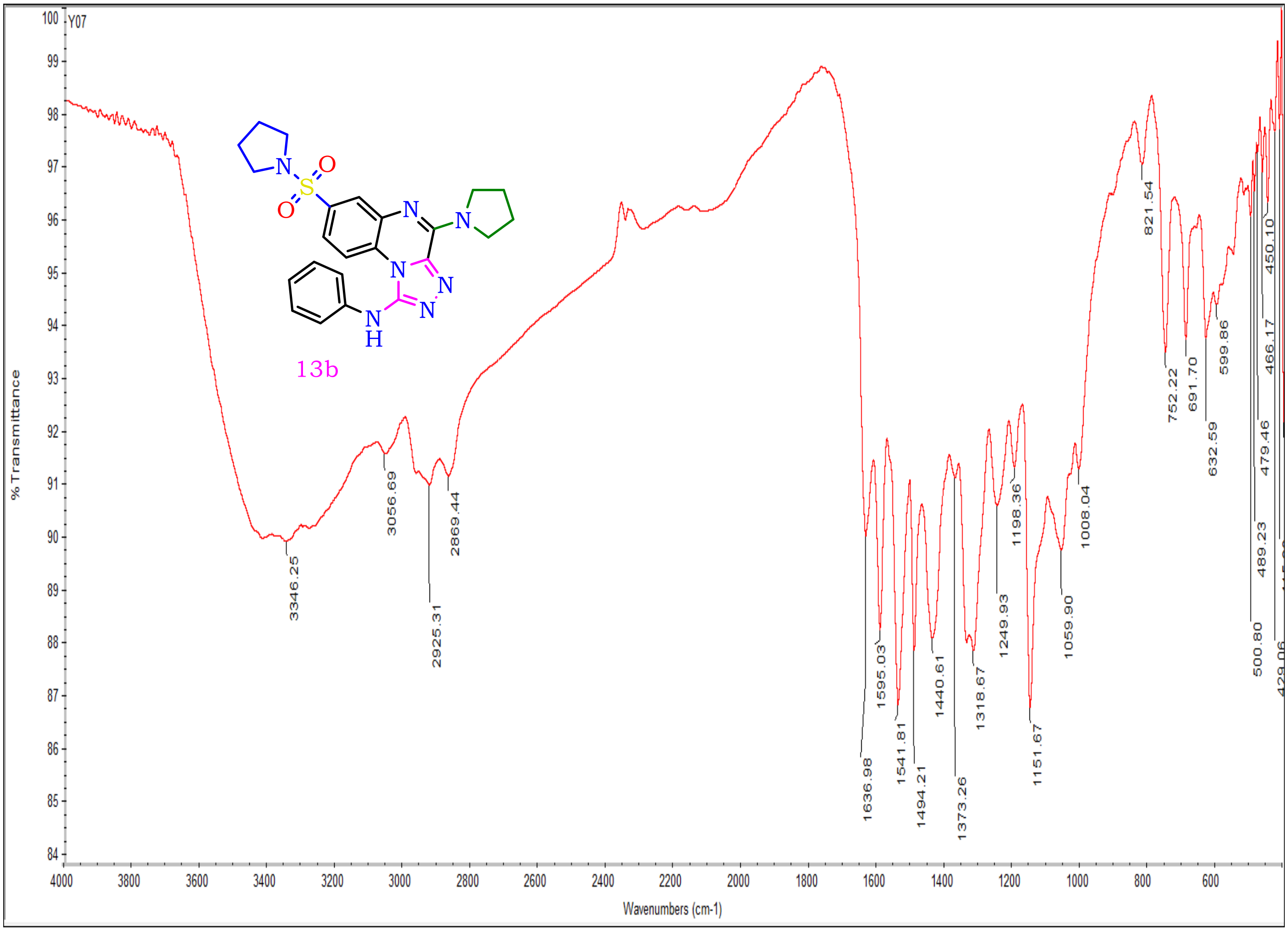

Figure SI32: IR spectrum of compound **13b**

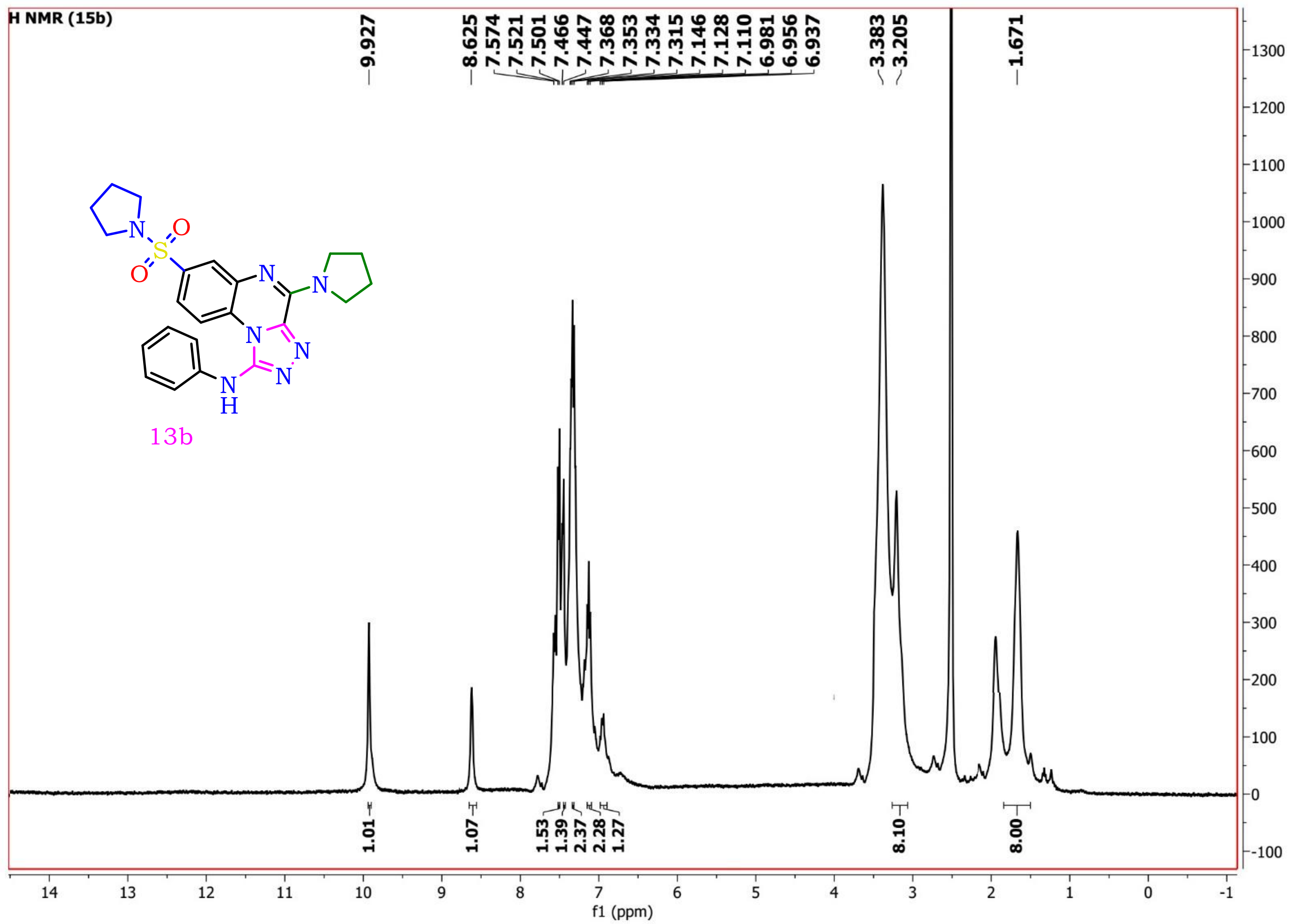

Figure SI33: ^{1}H NMR spectrum of compound **13b**

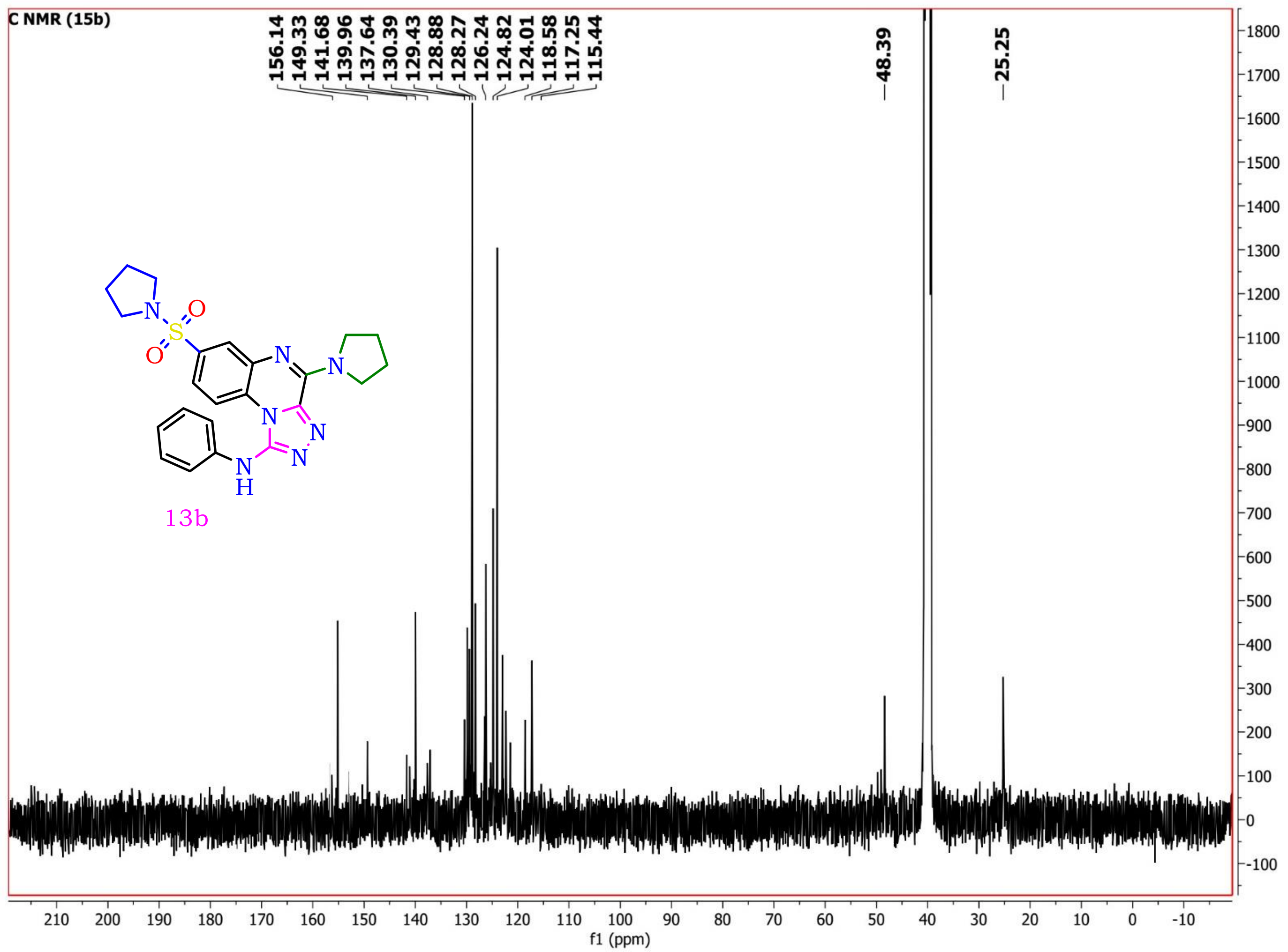

Figure SI34: ^{13}C NMR spectrum of compound **13b**

** Biological Evaluation

α-Amylase inhibitory assay (Wickramaratne et al., 2016)

The anti-diabetic activity will be assayed by α-amylase inhibitory assay according to the 3.5-dinitrosalicylic acid (DNSA) method for calculating the percentage of α-amylase inhibition (%) using Acarbose as a standard drug. In brief, 0.5 ml of test solution will be mixed with 0.5 ml of α-amylase solution (0.5 mg/ml) with buffer (Na_2HPO_4/NaH_2PO_4 (0.02 M), NaCl (0.006 M) at pH 6.9) to give concentrations ranging from 25 to 800 μg/mL. The mixture will be incubated at room temperature for 10 min and 200 μL of the starch solution (1% in water (w/v) buffer (Na_2HPO_4/NaH_2PO_4 (0.02 M), NaCl (0.006 M) at pH 6.9)) will be added. The reaction will be terminated by adding 200 μL DNSA (coloring) reagent (12 g of sodium potassium tartrate tetrahydrate in 8.0 mL of 2 M NaOH and 20 mL of 96 mM of DNSA solution). At this time, the test tubes will be placed in a boiling water bath (100 °C) for 10 min and the mixture will be cooled to ambient temperature and will be diluted with 5 mL of distilled water, and the absorbance will be measured at 540 nm using a UV-Visible spectrophotometer.

The blank with 100% enzyme activity will be prepared by replacing the plant extract with 200 μL of the buffer. A blank reaction will be similarly prepared using the plant extract at each concentration in the absence of the enzyme solution. A positive control sample will be prepared using acarbose and the reaction will be performed similarly to the reaction with organic compound as mentioned above. Absorbance of blank (buffer instead of analyte and amylase solution) and control (buffer instead of extract) samples will be also determined. Acarbose will be used as standard drug. Percentage of α-amylase inhibition will be calculated using the following equation: The α-amylase activity inhibition %:

$$[(Ab_{control} - Abc_{blank}) - (Ab_{sample} - Abs_{blank})/(Ab_{control} - Abc_{blank})] \times 100$$

Where, $\mathbf{Ab_{control}}$: absorbance of control; $\mathbf{Abc_{blank}}$: absorbance of control blank; $\mathbf{Ab_{sample}}$: absorbance of sample; and $\mathbf{Abs_{blank}}$: absorbance of sample blank.

α-Glucosidase inhibitory assay (Pistia-Brueggeman and Hollingsworth, 2001)

Five μl of the α-glucosidase solution (10units ml^{-1}, 0.1$moll^{-1}$ potassium phosphate buffer, pH 6.8) will be pre-mixed with 10μl of sample solution at different concentrations (in 10% DMSO) in 620 μl of 0.1$moll^{-1}$ potassium phosphate buffer (pH 6.8). Following incubation at 37.5°C for 20min, 10μl of *p*-nitro phenyl glucopyranoside (pNPG, 10$mmoll^{-1}$) as substrate will be added to the mixture to start the reaction. The reaction mixture will be incubated at 37.5°C for 30min, followed by addition of 650μl of 1$moll^{-1}$ Na_2CO_3 solution to terminate the reaction. The amount of released product (*p*-nitro phenol) will be measured at 410 nm using a UV spectrometer (UV-2550, Shimadzu, Japan) to estimate the enzymatic activity. For all tests, the inhibition assay will be performed in triplicate. Inhibitory activity will be calculated by the following equation:

$$\alpha\text{-glucosidase inhibitory activity } \mathbf{(\%) = [(A - B)/A] \times 100}$$

where: *A* will be the optical density of reaction blank, The reaction blank mixture contained the same volume of the buffer solution instead of the sample; *B* will be the optical density of the reaction in the presence of both α-glucosidase and peptide sample.

****

Anticholinesterase activity (anti-Alzheimer activity) (Ellman et al., 1961)

It will be assayed by quantifying activity of acetyl cholinesterase (AChE) enzyme using Ellman's method. The anti-Alzheimer's activity will be measured by calculating the percentage of acetylcholinesterase (AChE) enzyme inhibition (%) according to Ellman's method. The tested compounds will be dissolved in a 0.1 M phosphate buffer, pH 8. To a flat bottom 96-well plate, typical run consisted of, 5 µl of Acetylthiocholine (ATCh) (0.5 mM), 5 µl of 5, 5'-dithiobis-2-nitrobenzoic acid (DTNB) (0.03 mM) and 5 µl of the tested samples solution at the different concentrations evaluated, which will be mixed and incubated for 10 min at 30 °C. Then, 5 µl of AChE (0.3 U/ml) solution will be added to the initial mixture to start the reaction and then absorbance will be determined at 412 nm. A control run contained all the aforementioned constituents with the exception of the test extract. All experiments will be performed in triplicates. The concentration of the tested extract that will inhibit the hydrolysis of substrate ATCh will be determined by linear regression analysis.

References

M.N. Wickramaratne, J.C. Punchihewa, D.B.M. Wickramaratne, In-vitro alpha amylase inhibitory activity of the leaf extracts of adenanthera pavonina, BMC Complement. Altern. Med. 16 (2016) 466. https://doi.org/10.1186/s12906-016-1452-y.

G. Pistia-Brueggeman, R.I. Hollingsworth, A preparation and screening strategy for glycosidase inhibitors, Tetrahedron. 57 (2001) 8773–8778. https://doi.org/10.1016/S0040-4020(01)00877-8.

G.L. Ellman, K.D. Courtney, V. Andres, R.M. Featherstone, A new and rapid colorimetric determination of acetylcholinesterase activity, Biochem. Pharmacol. 7 (1961) 88–95. https://doi.org/10.1016/0006-2952(61)90145-9

Molecular docking simulation figures

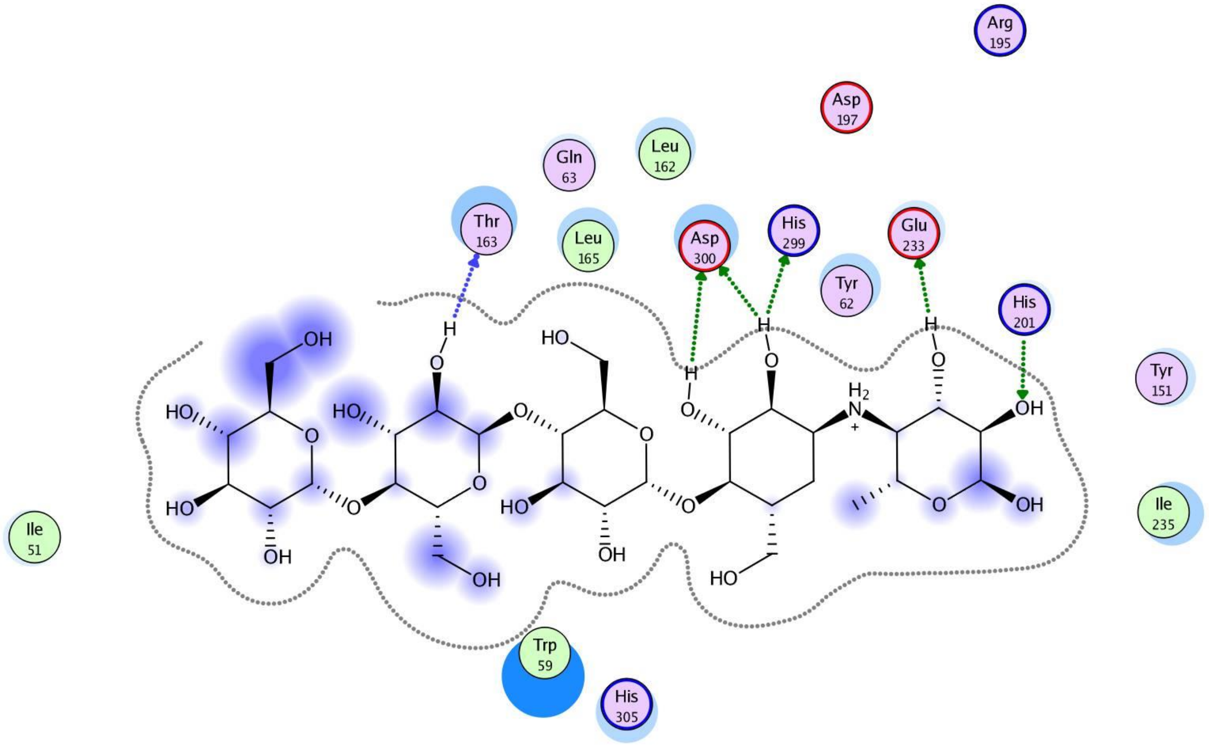

Figure SI35: 2D structure of **acarbose** inside the active site of α-amylase (PDB: 2QV4)

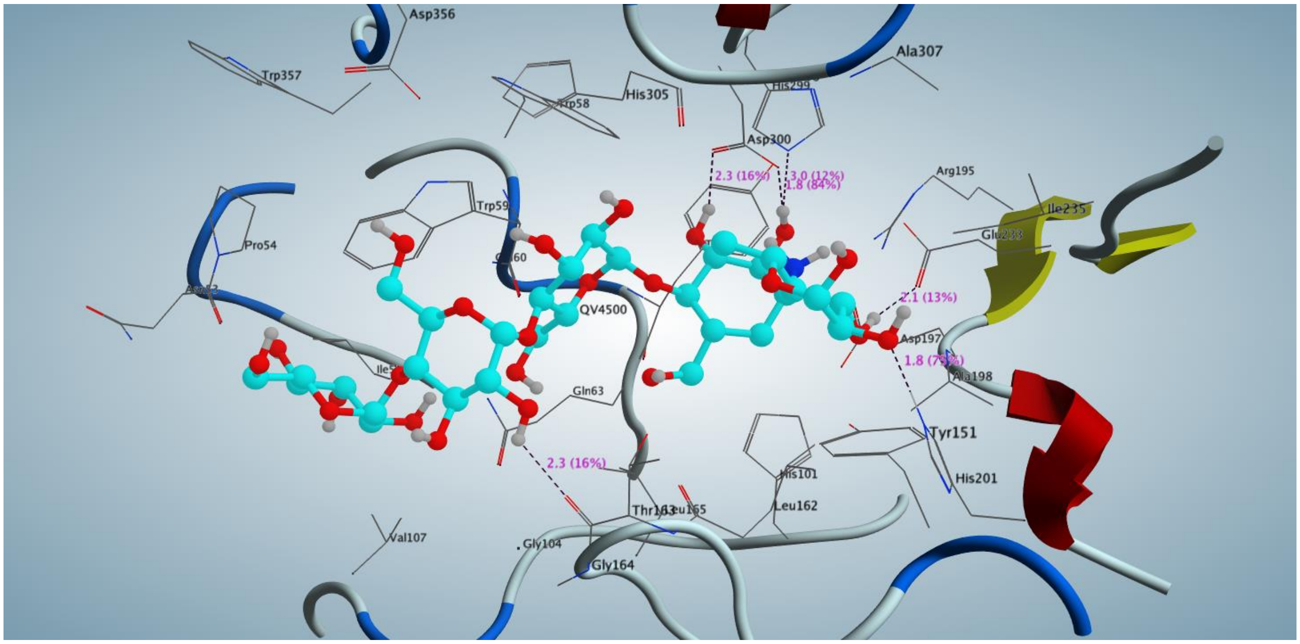

Figure SI36: 3D structure of **Acarbose** inside the active site of α-amylase (PDB: 2QV4)

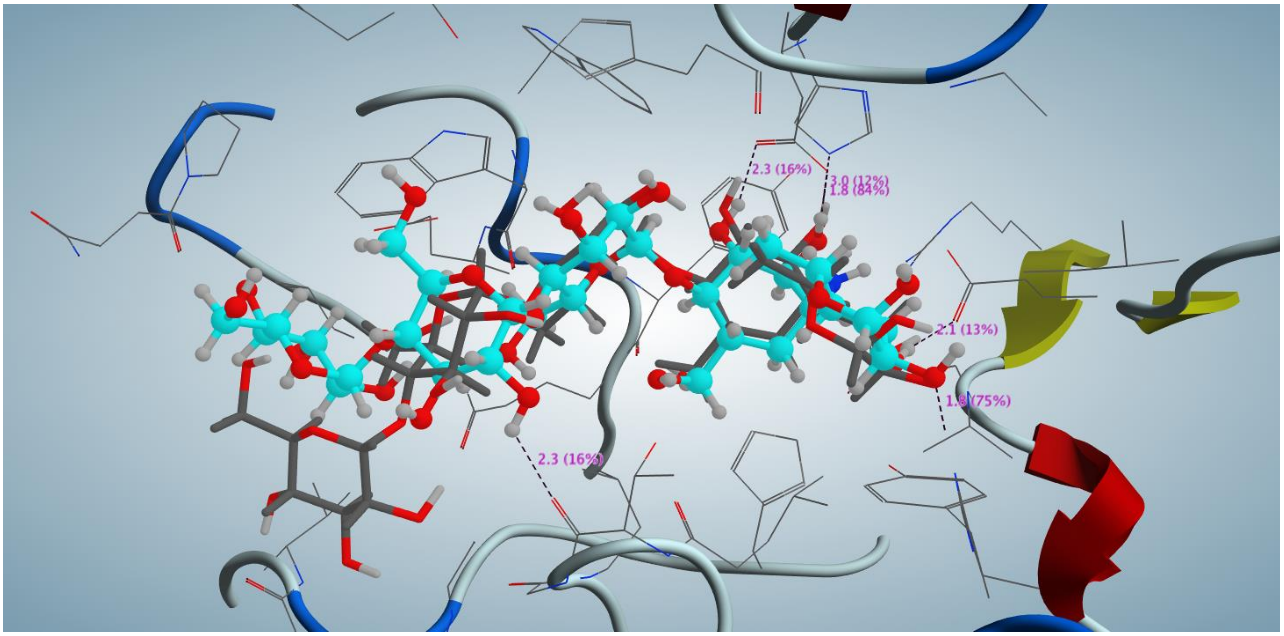

Figure SI37: 3D superimpose structure of **Acarbose** in the validation process inside the active site of α-amylase (PDB: 2QV4), where the validation process revealed binding affinity S =- 16.33 kcal/mol with RMSD = 1.36 Å

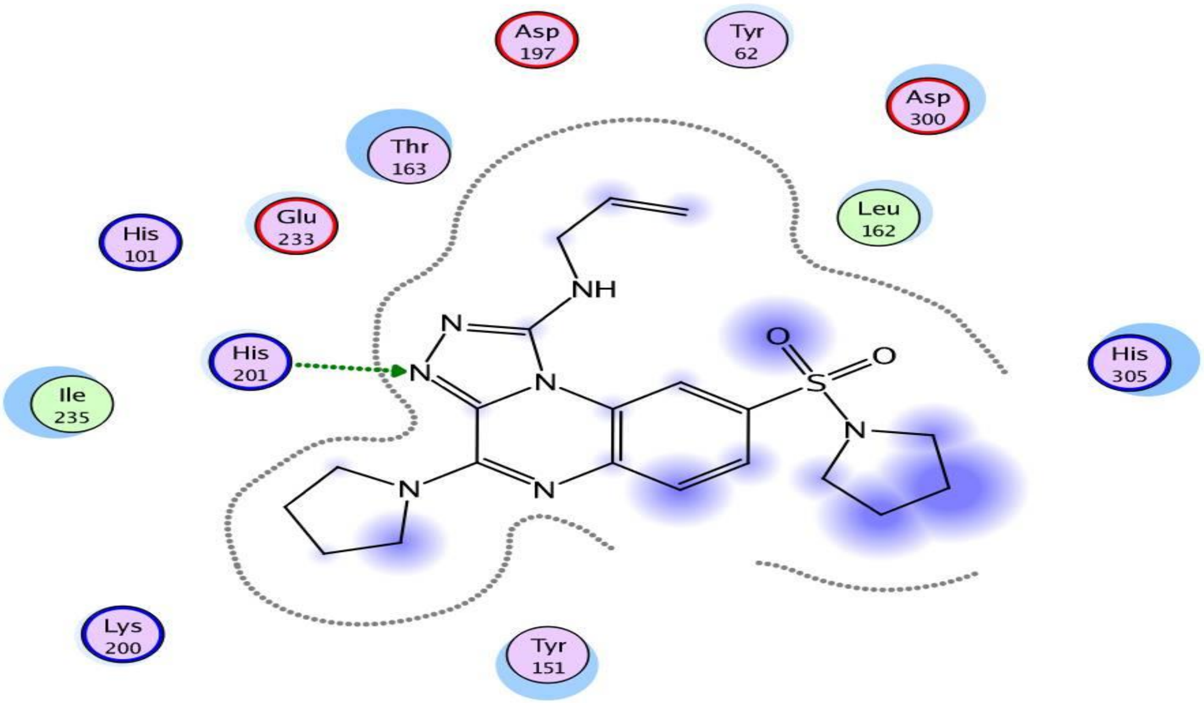

Figure SI38: 2D structure of the most active *N*-allyl-[1,2,4]triazolo[4,3-a]quinoxalin-1-aminederivative **10a** inside the active site of α-amylase (PDB: 2QV4)

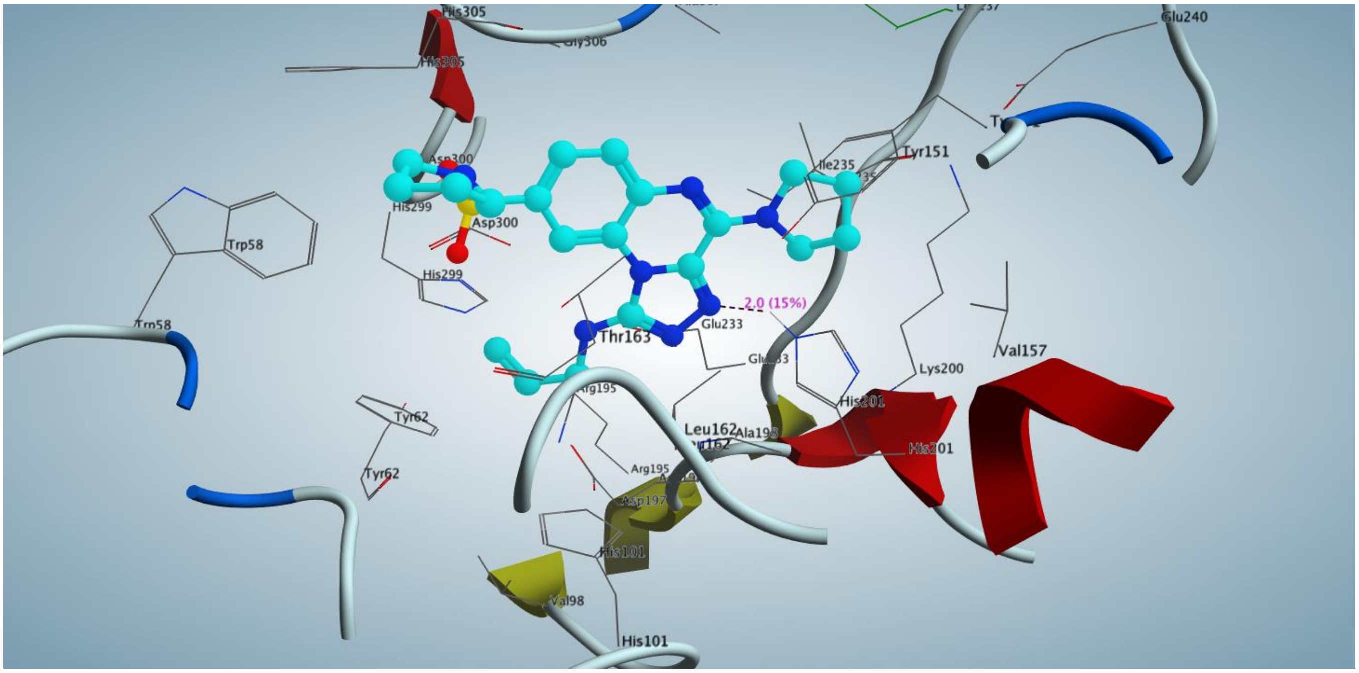

Figure SI39: 3D structure of the most active *N*-allyl-[1,2,4]triazolo[4,3-a]quinoxalin-1-aminederivative **10a** inside the active site of α-amylase (PDB: 2QV4)

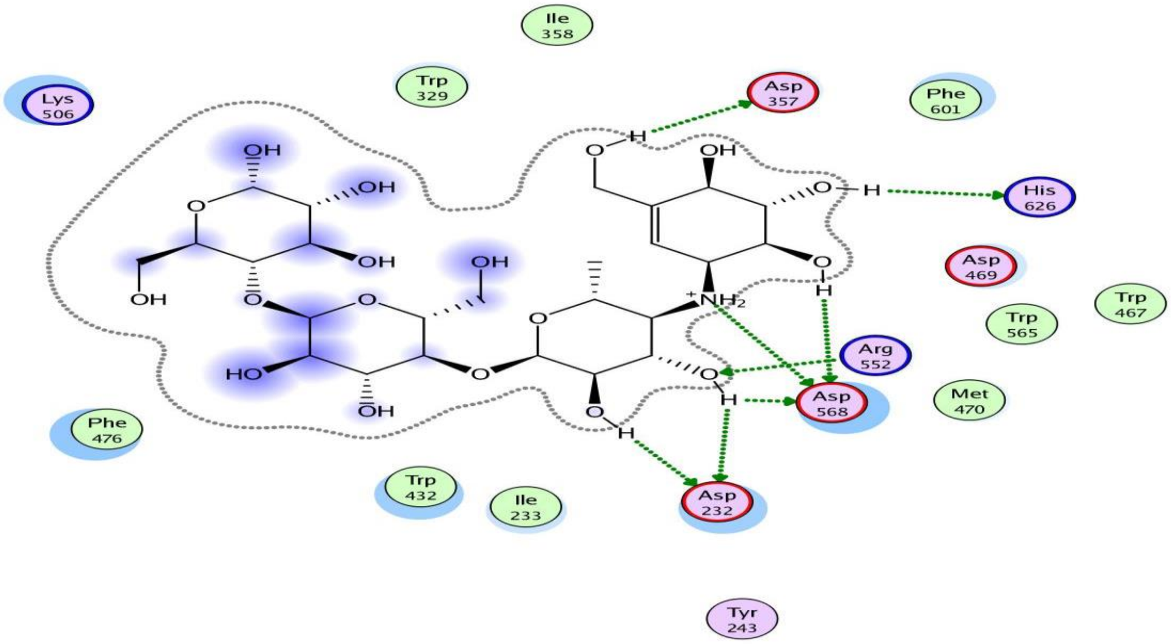

Figure SI40: 2D structure of **acarbose** inside the active site of α-glucosidase (PDB: 3W37)

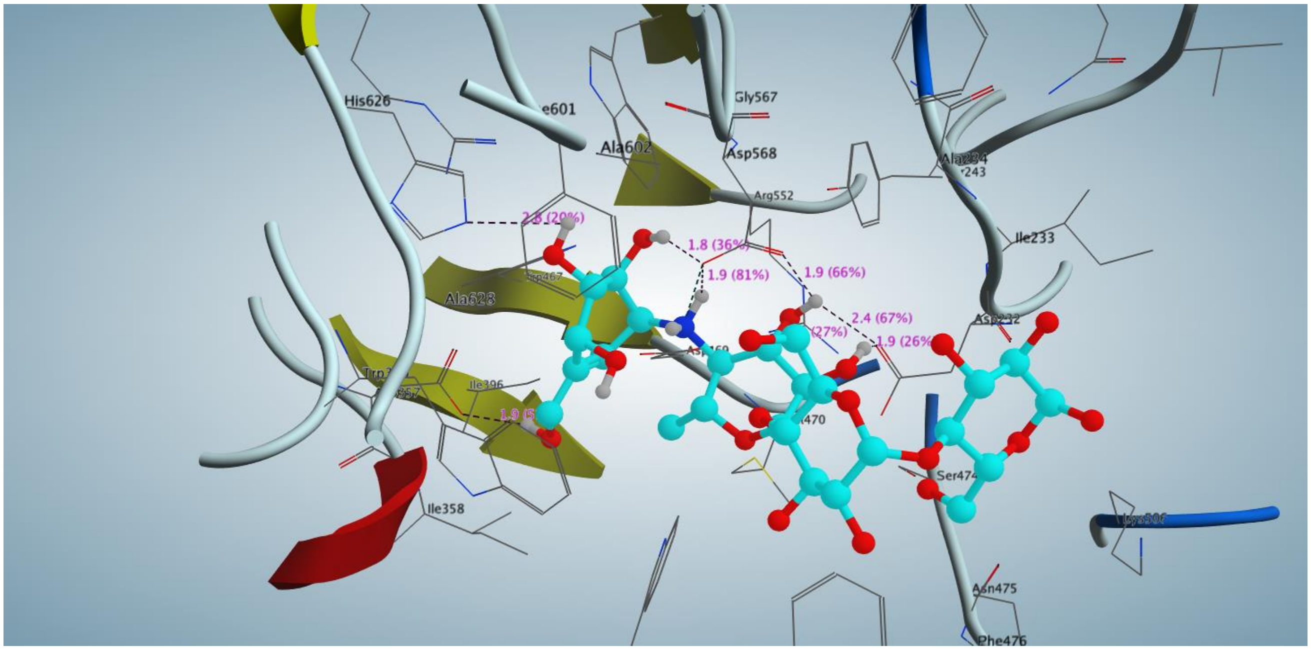

Figure SI41: 3D structure of **Acarbose** inside the active site of α-glucosidase (PDB: 3W37)

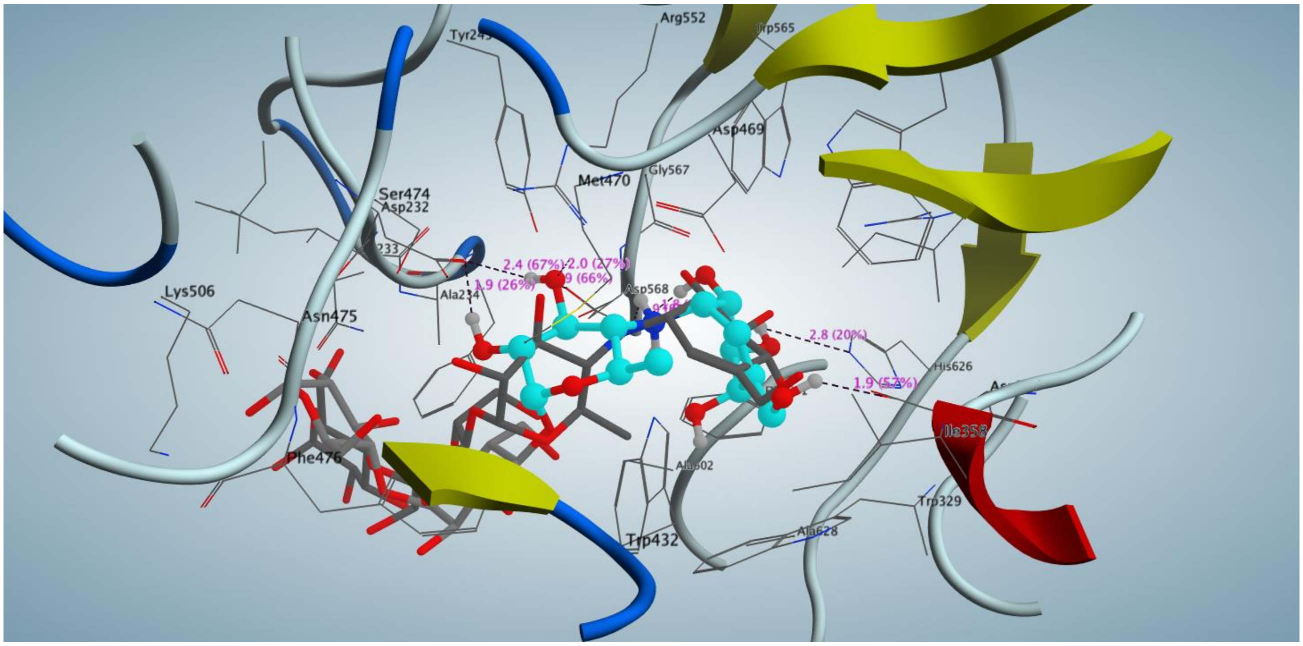

Figure SI42: 3D superimpose structure of **Acarbose** in the validation process inside the active site of α-glucosidase (PDB: 3W37), where the validation process revealed binding affinity S =-16.82 kcal/mol with RMSD = 2.357 Å

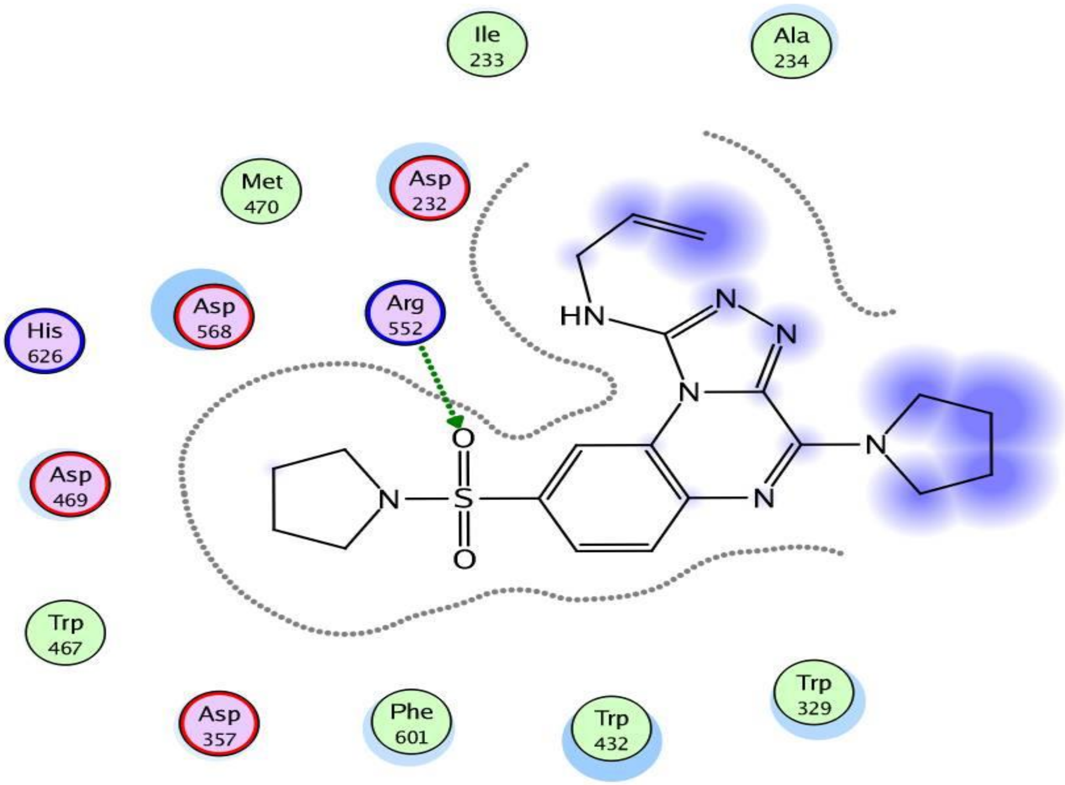

Figure SI43: 2D structure of the most active *N*-allyl-[1,2,4]triazolo[4,3-*a*]quinoxalin-1-aminederivative **10a** inside the active site of α-glucosidase (PDB: 3W37)

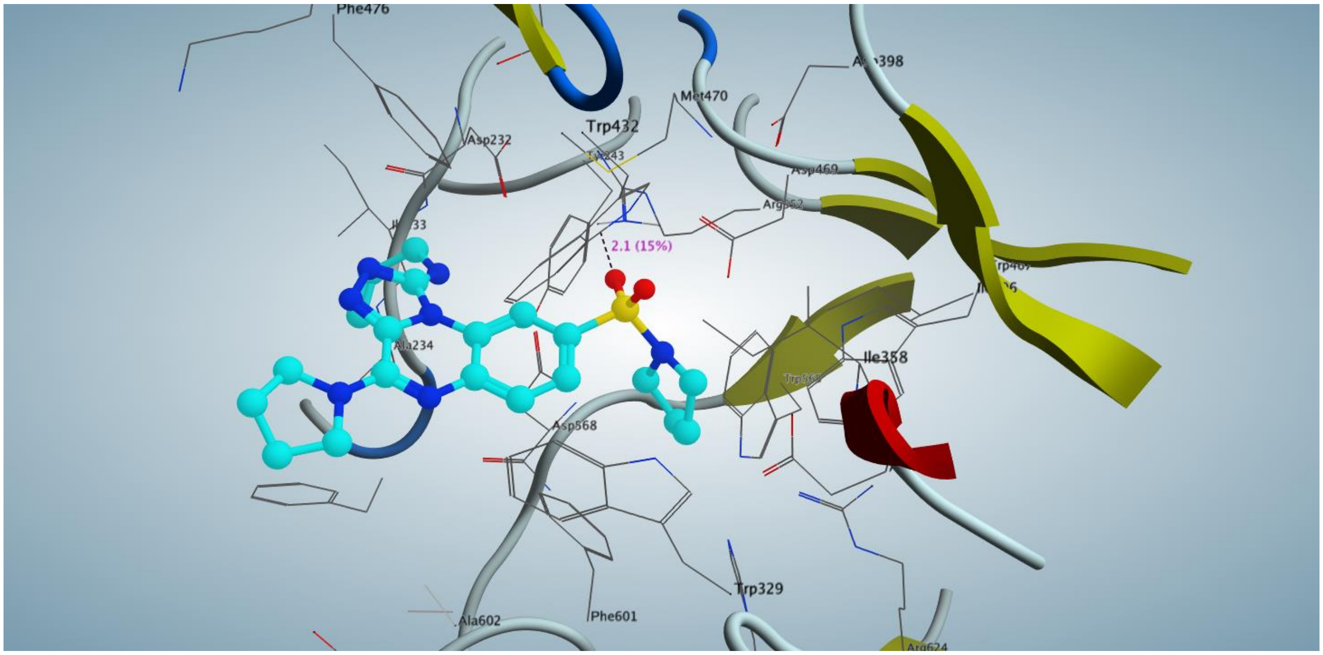

Figure SI44: 3D structure of the most active *N*-allyl-[1,2,4]triazolo[4,3-*a*]quinoxalin-1-aminederivative **10a** inside the active site of α-glucosidase (PDB: 3W37)

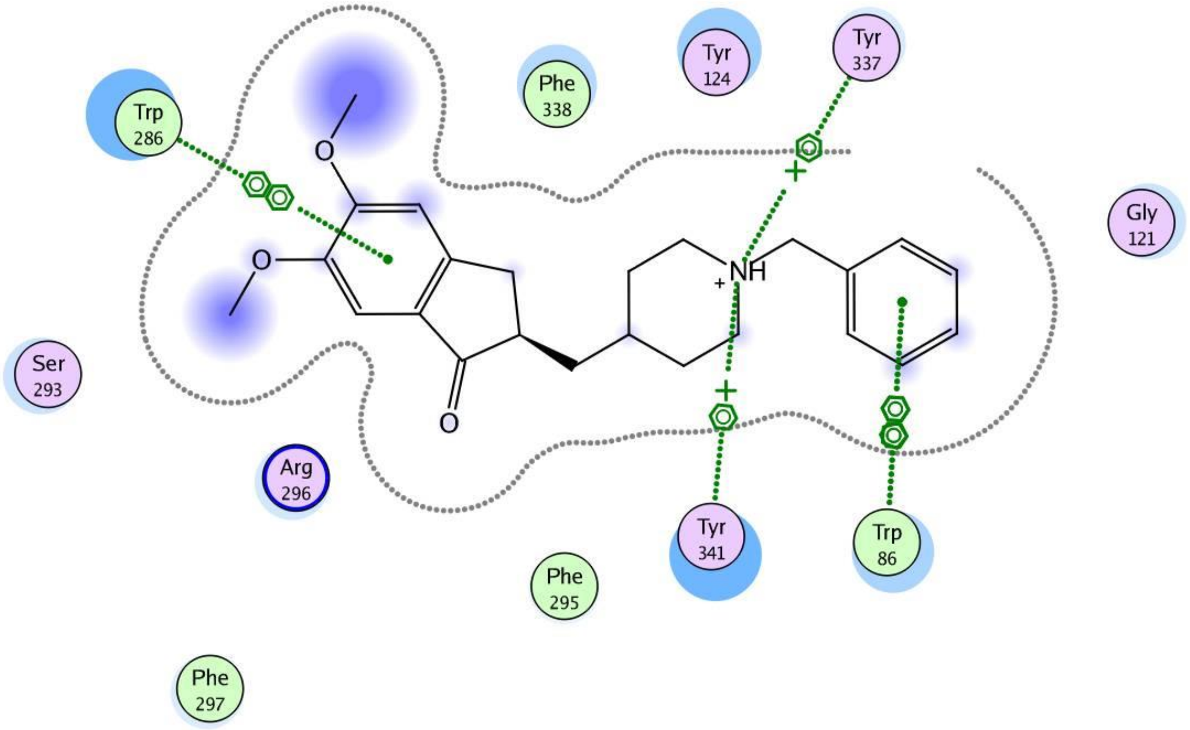

Figure SI45: 2D structure of **acarbose** inside the active site of acetylcholinesterase (AChE) (PDB: 4EY7)

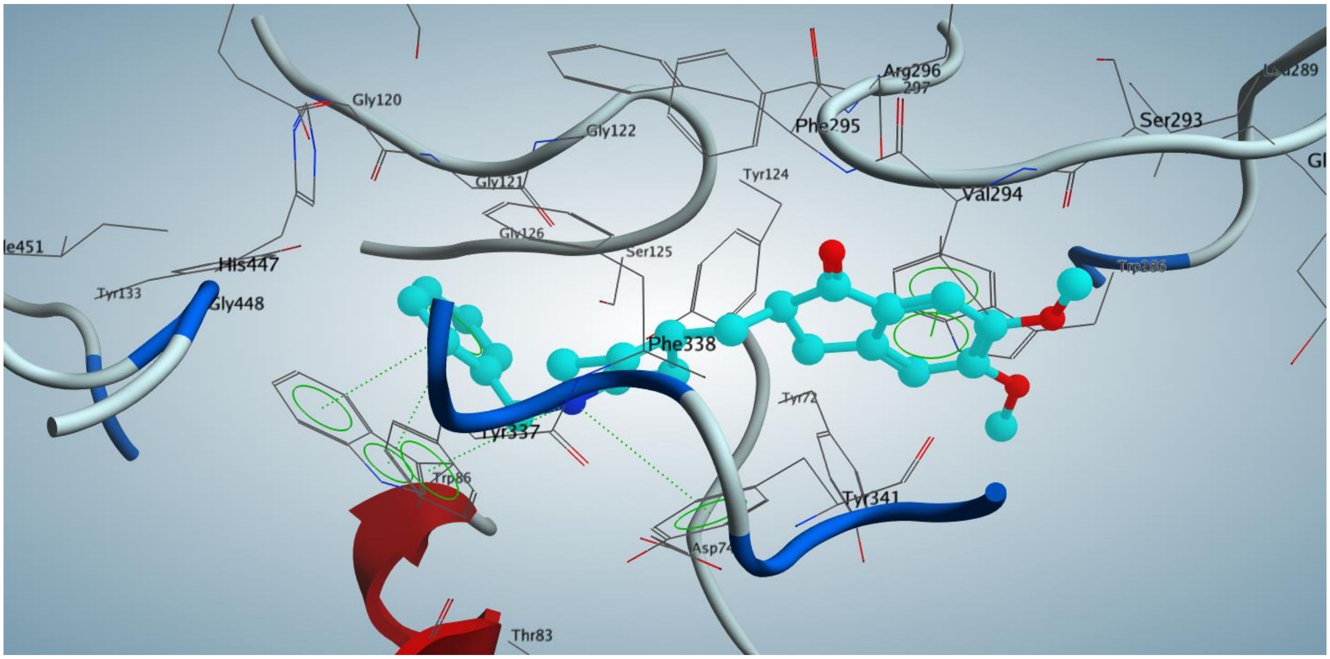

Figure SI46: 3D structure of **Acarbose** inside the active site of acetylcholinesterase (AChE) (PDB: 4EY7)

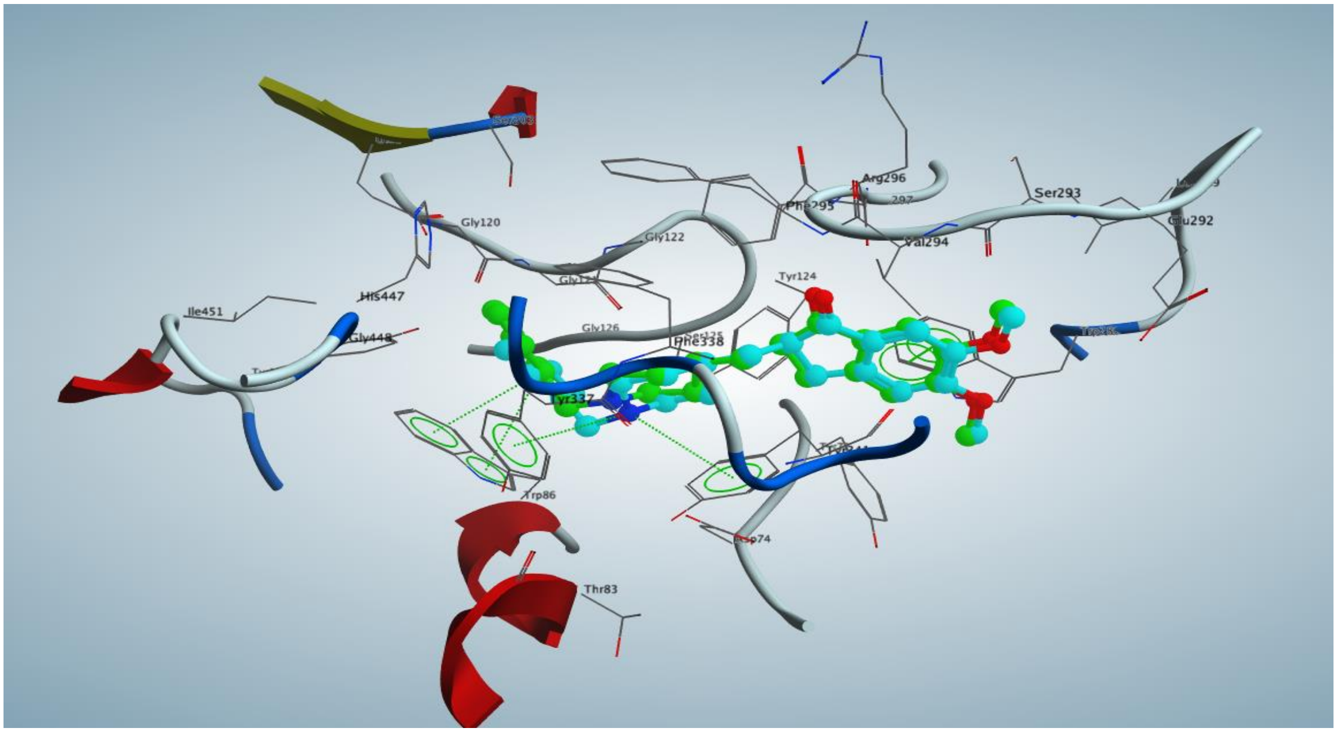

Figure SI147: 3D superimpose structure of **Acarbose** in the validation process inside the active site of acetylcholinesterase (AChE) (PDB: 4EY7) , where the validation process revealed binding affinity S = -11.027 kcal/mol with RMSD = 0.8064 Å

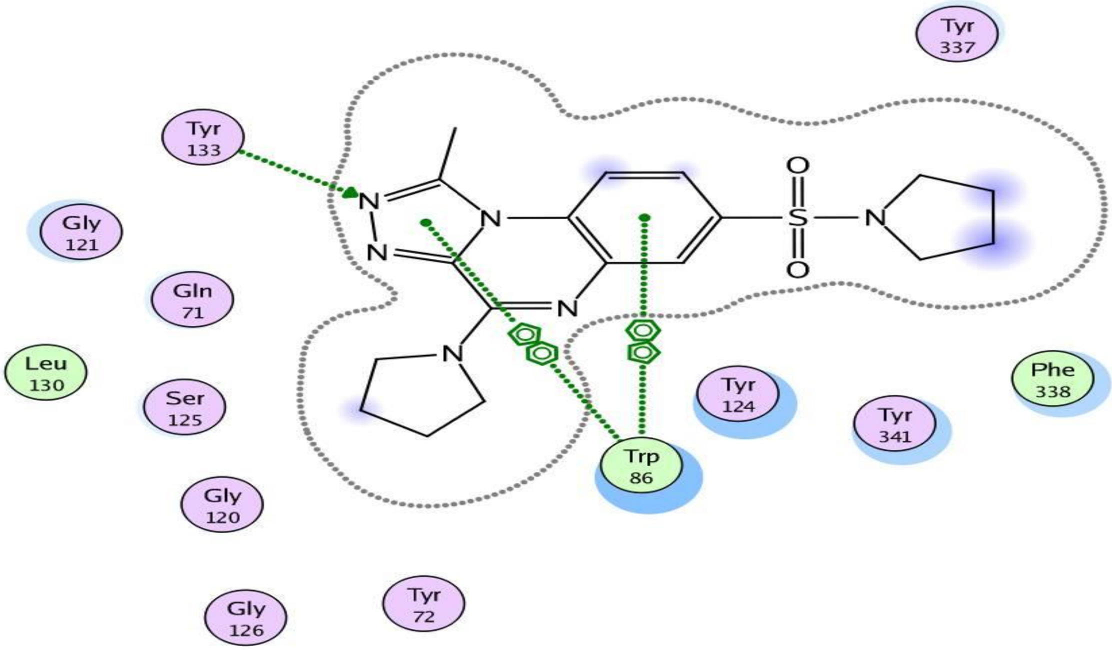

Figure SI148: 2D structure of the most active 1-methyl-4-(pyrrolidin-1-yl)-[1,2,4]triazolo[4,3-*a*]quinoxaline derivative **11b** inside the active site of acetylcholinesterase (AChE) (PDB: 4EY7)

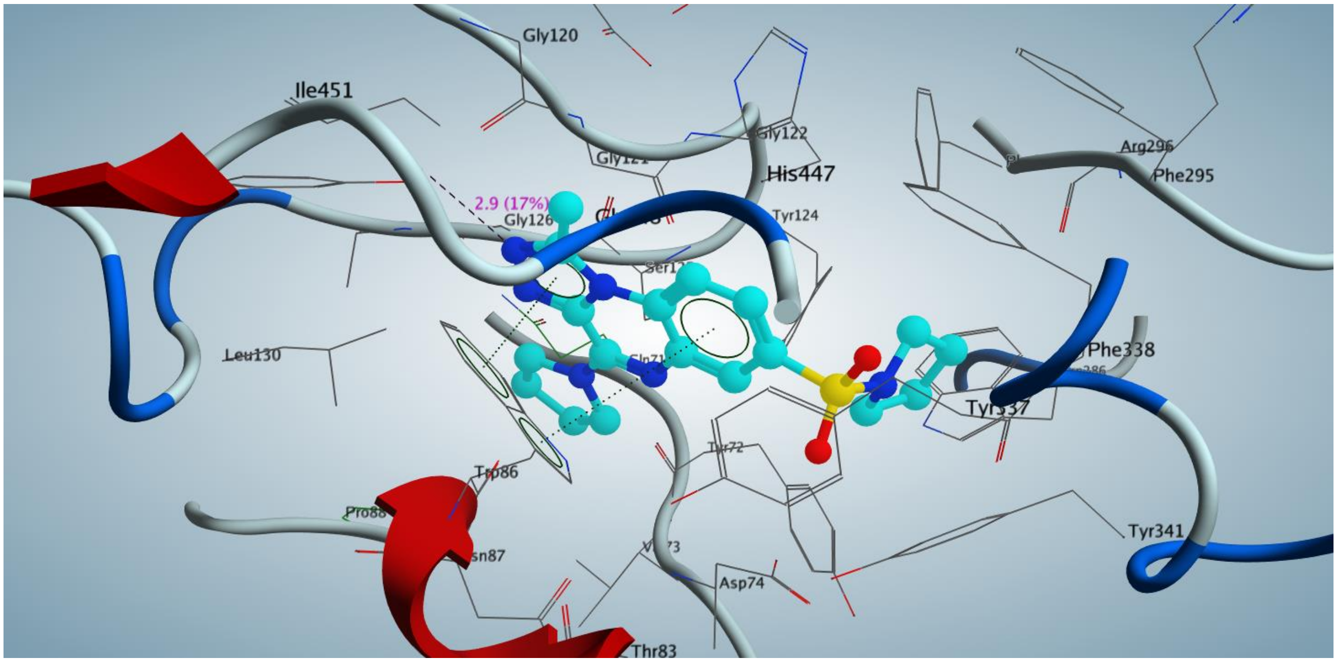

Figure SI149: 3D structure of the most active 1-methyl-4-(pyrrolidin-1-yl)-[1,2,4]triazolo[4,3-*a*]quinoxaline derivative **11b** inside the active site of acetylcholinesterase (AChE) (PDB: 4EY7)
